# BEAT CF pulmonary exacerbations core protocol for evaluating the management of pulmonary exacerbations in people with cystic fibrosis

**DOI:** 10.1186/s13063-023-07076-8

**Published:** 2023-03-22

**Authors:** Andre Schultz, Charlie McLeod, Scott Berry, Julie Marsh, Anne McKenzie, Mitch Messer, Jamie Wood, Ben Saville, Adam Jaffe, Sarath Ranganathan, Steve Stick, Peter Wark, Steve Webb, Tom Snelling

**Affiliations:** 1 Wal-yan Respiratory Research Centre, Telethon Kids Institute, University of Western Australia, Nedlands, Australia; 2 Department of Respiratory and Sleep Medicine, Perth Children’s Hospital, Nedlands, Australia; 3 Wesfarmers Centre of Vaccines and Infectious Diseases, Telethon Kids Institute, Nedlands, Australia; 4 Department of Infectious Diseases, Perth Children’s Hospital, Nedlands, Australia; 5 Berry Consultants, LLC, Austin, TX USA; 6 School of Population and Global Health, University of Western Australia, Nedlands, Australia; 7 Telethon Kids CONNECT, Telethon Kids Institute, Nedlands, Australia; 8 Department of Rehabilitation and Human Performance, Abilities Research Centre, Icahn School of Medicine, Mount Sinai, New York USA; 9 Discipline of Paediatrics and Child Health, Faculty of Medicine and Health, University of New South Wales, Kensington, Australia; 10 Sydney Children’s Hospital, Randwick, Australia; 11 Department of Paediatrics, University of Melbourne, Parkville, Australia; 12 Department of Respiratory and Sleep Medicine, Royal Children’s Hospital, Parkville, Australia; 13 Respiratory Diseases Research Group, Infection and Immunity, Murdoch Children’s Research Institute, Parkville, Australia; 14 Immune Health Program, Hunter Medical Research Institute, University of Newcastle, Callaghan, Australia; 15 Sleep Medicine Division, John Hunter Hospital, New Lambton Heights, Australia; 16 Australian and New Zealand Intensive Care Research Centre, Monash University, Clayton, Australia; 17 St John of God Hospital, Subiaco, Australia; 18 Sydney School of Public Health, The University of Sydney, Sydney, Australia

**Keywords:** Cystic fibrosis, Exacerbations, Lung function, FEV_1_, Clinical trial, Cohort, Platform, Management, Treatment

## Abstract

**Background:**

Cystic fibrosis (CF) is a rare, inherited, life-limiting condition predominantly affecting the lungs, for which there is no cure. The disease is characterized by recurrent pulmonary exacerbations (PEx), which are thought to drive progressive lung damage. Management of these episodes is complex and generally involves multiple interventions targeting different aspects of disease. The emergence of innovative trials and use of Bayesian statistical methods has created renewed opportunities for studying heterogeneous populations in rare diseases. Here, we present the protocol for the BEAT CF PEx cohort, a prospective, multi-site, perpetual, platform enrolling adults and children with CF. The BEAT CF PEx cohort will be used to evaluate the comparative effectiveness of interventions for the treatment of PEx requiring intensive therapy (PERITs), with a primary focus on short-term improvements in lung function. This will be achieved through the conduct of cohort-nested studies, including adaptive clinical trials, within the BEAT CF PEx cohort. This protocol will outline key features of the BEAT CF PEx cohort, including the design, implementation, data collection and management, governance and analysis, and dissemination of results.

**Methods:**

This platform will be conducted across multiple sites, commencing with CF treatment centers in Australia. People of all ages with a clinical diagnosis of CF will be eligible to participate, except those who have previously received a lung transplant. Data including demographic and clinical information, treatment details, and outcomes (including safety, microbiology, and patient-reported outcome measures including quality of life scores) will be systematically collected and securely stored via a digital centralized trial management system (CTMS). The primary endpoint is the absolute change in the percentage predicted forced expiratory volume in 1 s (ppFEV_1_) from the commencement of intensive therapy to 7 to 10 days afterwards.

**Discussion:**

The BEAT CF PEx cohort will report clinical, treatment, and outcome data for PEx among people with CF and is intended to serve as a core (master) protocol for future nested, interventional trials evaluating treatment(s) for these episodes. The protocols for nested sub-studies are beyond the scope of this document and will be reported separately.

**Trial registration:**

ANZCTR BEAT CF Platform – ACTRN12621000638831. Registration date: Sept. 26, 2022.

**Supplementary Information:**

The online version contains supplementary material available at 10.1186/s13063-023-07076-8.

## Administrative information

Note: the numbers in curly brackets in this protocol refer to SPIRIT checklist item numbers. The order of the items has been modified to group similar items (see http://www.equator-network.org/reporting-guidelines/spirit-2013-statement-defining-standard-protocol-items-for-clinical-trials/).

**Table:** 

Title {1}	BEAT CF pulmonary exacerbations core protocol for evaluating management of pulmonary exacerbations in people with cystic fibrosis.
Trial registration {2a and 2b}.	ANZCTR BEAT CF Platform – ACTRN12621000638831 accessible here.
Protocol version {3}	Version 7. 8^th^ November 2021.
Funding {4}	Funding is provided by a Medical Research Future Fund Lifting Clinical Trials and Registries Capacity Grant (GNT1152376).
Author details {5a}	A/Prof André Schultz Dr Charlie McLeod Dr Scott Berry Dr Julie Marsh Ms Anne McKenzie Mr Mitch Messer Jamie Wood Dr Ben Saville Prof Adam Jaffe Prof Sarath Ranganathan Prof Stephen Stick Prof Peter Wark Prof Steve Webb Prof Tom Snelling
Name and contact information for the trial sponsor {5b}	University of Sydney, New South Wales, Australia, 2006 P: +61 2 8627 9280
Role of sponsor {5c}	As Sponsor, the University of Sydney assumes ultimate responsibility for the initiation, conduct, management, quality and integrity of data and financing, including for any nested studies. Other activities, including but not limited to design, conduct, safety monitoring, and reporting, are delegated to the Steering Committee.

## Introduction

### Background and rationale {6a}

Cystic fibrosis (CF) is an inherited, life-limiting disease affecting multiple organs, predominantly the lungs. Pulmonary exacerbations (PEx) are a hallmark of disease and are thought to drive progressive lung damage; it is estimated that a quarter of people fail to fully recover lung function after these episodes [1]. While there is no consensus definition for PEx [1], episodes generally involve a deterioration in lung function and new or worsening respiratory and systemic symptoms. Management of PEx is complex, invasive, and often requires prolonged hospitalization [2]. While the use of intravenous antibiotic therapy is a cornerstone of management during hospitalization, the approach is generally multimodal and involves one or more of intensified airway clearance therapies via physiotherapy with or without muco-active agents, immune modulation, optimization of nutrition, and psychosocial support [2–6]. Important knowledge gaps remain regarding the optimal management of these episodes, and these gaps drive variations in care for people with CF both within and between centers. Management of PEx has been identified as a research priority among people with CF [7].

Randomized controlled trials are rightfully considered the gold standard for generating evidence to inform clinical practice and policy [8]. However, traditional trials can be burdensome, inefficient, and limited to addressing just one management question at a time. Trials that are burdensome (for participants or clinicians) might inadvertently select for a non-representative study population, meaning any results may not be generalizable. This presents challenges for evaluating complex and multi-component interventions in a highly heterogeneous patient population.

Newer approaches to clinical research offer an opportunity to generate high-quality evidence for guiding patient management, including in complex and rare diseases. These innovations, including nesting of studies within registries or large cohorts, pragmatism and healthcare embedding, and outcome or response-adaptation, focus on informing real world decision-making, may be more conducive to high levels of participation, and may offer greater flexibility and statistical efficiency than traditional clinical trials [9, 10]. Such approaches enable multiple treatment questions to be addressed concurrently or sequentially, according to research priorities and prespecified protocol adaptation rules [9].

Here, we present a core protocol for studying the management of PEx in the BEAT CF (Bayesian Evidence Adaptive Treatment for people with Cystic Fibrosis) platform. The PEx core protocol sets out the establishment of a prospective, multi-site cohort designed to perpetually enroll and follow-up people with CF and systematically capture data on the management and outcomes of any PEx requiring intensive therapy (PERIT). Here, a PERIT is pragmatically defined as an acute or subacute deterioration in lung function or symptoms resulting in (possibly multimodal) therapy comprising initiation of at least one intravenous antibiotic. It is anticipated that most cohort participants will become eligible to participate in cohort-nested sub-studies, including a PEx Treatment Platform in which selected aspects of PERIT management will be randomly assigned.

**Fig. 1:**
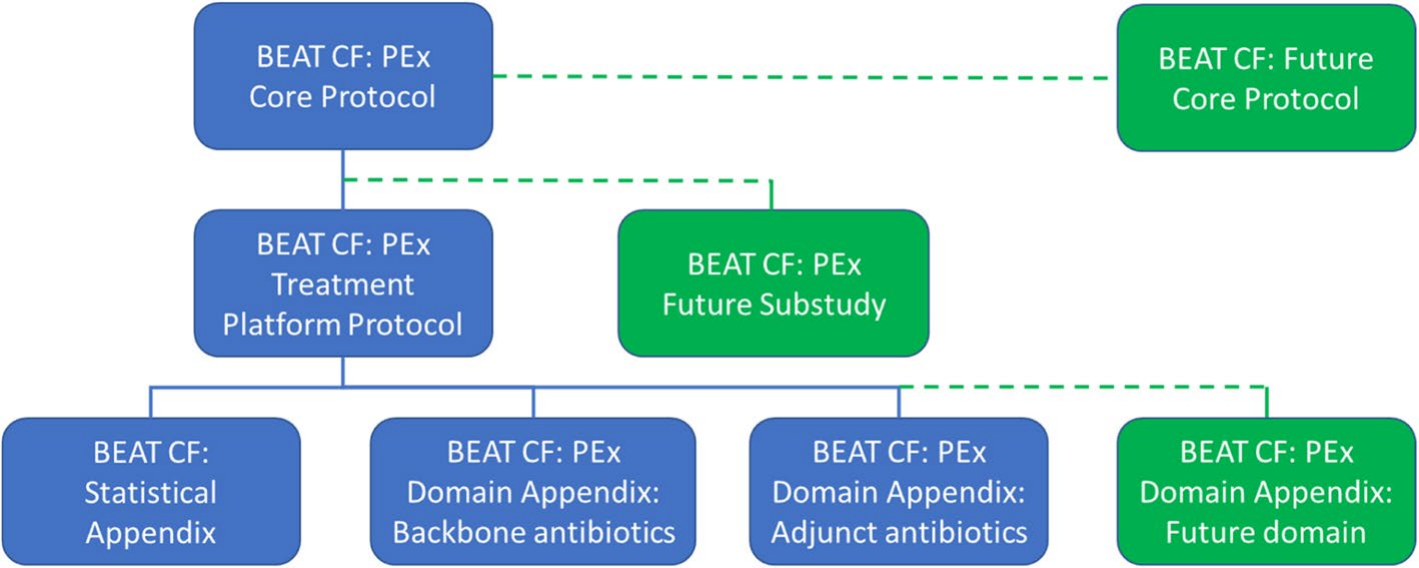
BEAT CF protocol documentation for the BEAT CF PEx cohort and related and nested sub-studies

The protocol documentation for BEAT CF is modular and hierarchical (Fig. 1) to facilitate nesting of the PEx Treatment Platform and other cohort-nested sub-studies, to minimize redundancy in the documentation, and to maximize consistency and standardization across the cohort and any nested projects. Here, we describe the core objectives, endpoints, data collection, and study procedures for the PEx cohort, along with plans for the analysis and reporting of their data. All current approved versions of the BEAT CF protocol documentation are available at https://www.beatcf.org.au/beat-cf/.

### Objectives {7}

The primary objective of the PEx cohort is to identify the effectiveness, or comparative effectiveness, of alternative interventions that are currently in routine use, or proposed for future use, in the management of PERITs in children and adults with CF, with respect to short-term improvements in lung function. For the purpose of the PEx cohort, “intensive therapy” means therapy comprising the initiation of at least one antibiotic by the intravenous route.

Secondary objectives are to identify the effectiveness, or comparative effectiveness, of alternative interventions on:

1. Long-term improvements in lung function
2. Short and long-term improvements in symptoms, quality of life (QoL) and life expectancy
3. Short and long-term healthcare utilization, including for management of PERITs
4. The toxicity and comparative toxicity of alternative interventions
5. The cost-effectiveness of alternative interventions
6. The impact of alternative interventions on the generation or selection of antibiotic resistant bacteria and the impact of this on lung function and QoL

### Trial design {8}

**Fig. 2:**
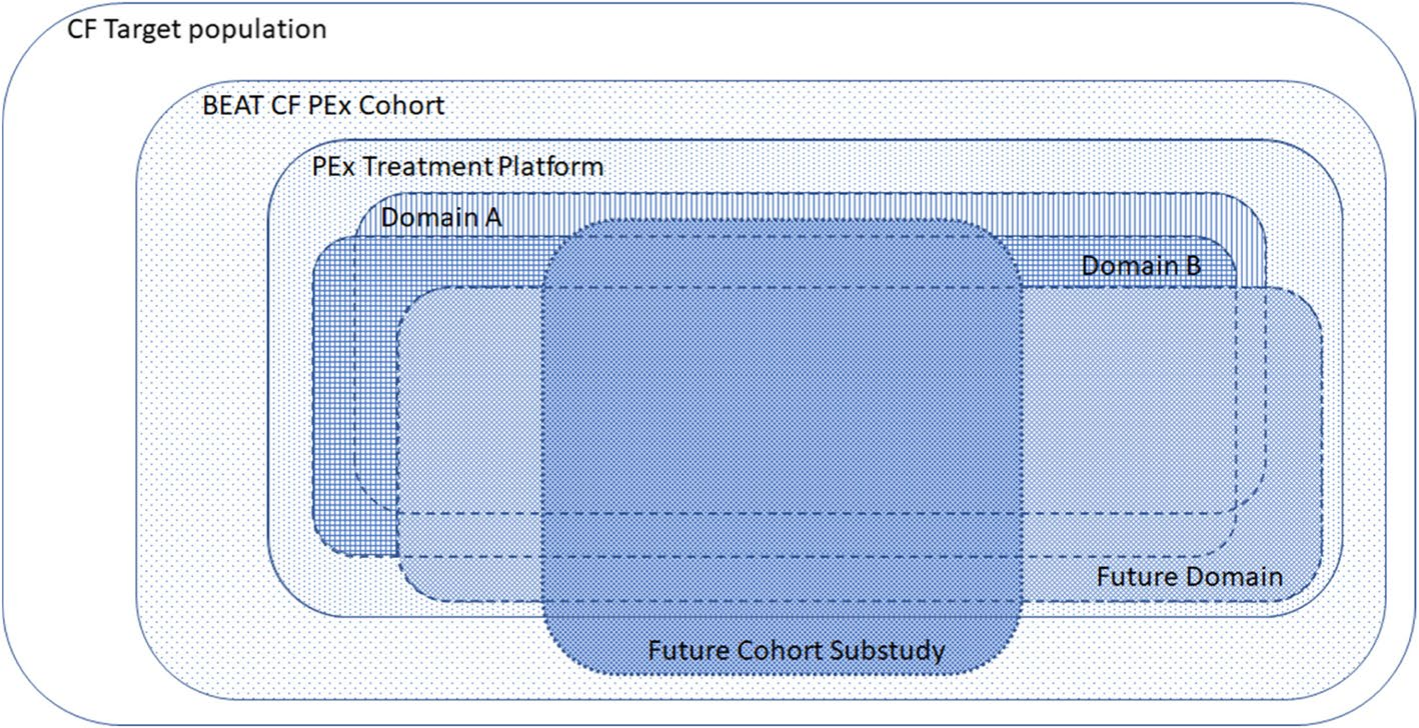
The relationship between the BEAT CF cohort and nested substudies

The PEx core protocol describes the establishment of a prospective, multi-site cohort that is intended to perpetually enroll children and adults with CF to serve as an adaptive platform for evaluating the (comparative) effectiveness of interventions for PEx, including through cohort-nested sub-studies. The PEx cohort is intended to be representative of the total CF population (Fig. 2). The PEx cohort will collect data regarding participant and disease characteristics, details of PERIT management, and clinical outcomes to identify, explain and quantify differences in treatment outcome(s) among people with CF treated for PEx.

## Methods: participants, interventions, and outcomes

### Study setting {9}

The PEx cohort enrolls participants from CF treatment centers, comprising hospitals, their regional outreach clinics, and ambulatory care facilities. While enrolment is currently limited to Australian centers, the protocol permits extension to international sites. The addition of new sites is contingent upon the successful completion of a site feasibility assessment. All project staff are required to undergo good clinical practice training and project-specific training prior to their involvement. As of August 2022, ten Australian sites are participating. An updated list of participating sites is available on the study website located at https://www.beatcf.org.au/beat-cf/.

### Eligibility criteria {10}

All children and adults with a confirmed diagnosis of CF are eligible for inclusion in the PEx cohort, excluding those with a history of lung transplantation. Additional eligibility criteria (typically exclusion criteria) may apply to cohort-nested sub-studies, and these will be detailed in their relevant subprotocols or appendices. Co-enrolment of participants in other studies is permitted, whether they are formally nested in the PEx cohort or not, including interventional trials, except when there is a clear threat to the validity of either study, or where co-enrolment would materially influence the risk to participants.

### Who will take informed consent? {26a}

Informed consent for participation in the PEx cohort is obtained by authorized project staff. Electronic and/or written versions of the participant information sheet and consent form will be presented to the participant and/or legal guardian detailing background information about the project, requirements of participants, potential risks, and the anticipated outcomes and constraints. Participation will be voluntary and will not impact on patient care. Participants will be free to terminate their involvement at any stage. Consent via written or electronic signature will be required from the participant and/or legal guardian prior to inclusion.

### Additional consent provisions for collection and use of participant data and biological specimens {26b}

As the PEx cohort is designed to be perpetual, participant consent will be regularly assessed to ensure it is appropriate and current. When a participant turns 18 years old, they are invited to provide informed consent as an adult. Children under 18 years old may provide their own consent, in consultation with their legally responsible caregiver. No additional investigations (including laboratory or radiological tests) outside of routine care are performed for the PEx cohort but may be required by participation in a nested sub-study. Participation in any nested studies is voluntary, and any additional procedures beyond routine care will require additional consent.

### Interventions

#### Explanation for the choice of comparators {6b}

The PEx cohort is observational; therefore, no specific interventions are defined for this cohort. For the purpose of reporting a sub-study from the PEx cohort, a PERIT intervention is considered to be any treatment, procedure, or strategy used in the acute management of a PERIT. PERIT interventions in the sub-studies are grouped into therapeutic domains comprising sets of mutually exclusive interventions, for example, sets of backbone antibiotics, sets of adjunctive antibiotics, sets of mucolytic therapies, sets of alternative physiotherapeutic regimens, and sets of immunomodulator treatments.

#### Intervention description {11a}

Participants in the PEx cohort receive usual care in each therapeutic domain as directed by their treating medical team, except as required by participation in a nested sub-study where certain interventions may be assigned. Any assignment of interventions and their relevant comparators will be specified in the relevant subprotocols or appendices for those sub-studies. All current approved versions of the BEAT CF protocol documentation are available at https://www.beatcf.org.au/beat-cf/.

#### Criteria for discontinuing or modifying allocated interventions {11b}

Participants in the PEx cohort may discontinue or modify one or more PERIT intervention(s) at any time in consultation with their treating clinician, except as required by participation in a nested sub-study. They may also discontinue from the PEx cohort entirely if (i) they and/or their legal guardian requests withdrawal or (ii) their treating medical team decides continued participation is not in their best interests. Consent to the use of study data, including data collected until the time of discontinuation and data to inform primary and secondary outcomes, will be requested specifically from participants or their legal representative. Following discontinuation, participants will receive all care as directed by their treating medical team. Any procedures for withdrawal from or modification of any assigned interventions will be specified in their relevant subprotocols.

#### Strategies to improve adherence to interventions {11c}

As the PEx cohort is observational, no specific strategies will be used to ensure adherence to prescribed therapies. Any strategies used to improve adherence to any assigned interventions in sub-studies will be detailed in the relevant subprotocols.

#### Relevant concomitant care permitted or prohibited during the trial {11d}

All PERIT management decisions are at the discretion of the treating clinician, except as required by participation in a nested sub-study. Any prohibited interventions will be specified for participants in the relevant subprotocols or appendices. Data regarding interventions, including concurrent therapies are recorded.

#### Provisions for post-trial care {30}

Participants in the PEx cohort will receive usual care for each PERIT and between PERIT episodes, except as required by participation in any nested sub-studies. Any procedures for post-intervention sub-study care will be specified in the relevant subprotocols.

#### Outcomes {12}

The primary estimand (inferential target for estimation) for each participant, for each PERIT, is defined by:

1. *Study population:* People with CF with a PERIT who can perform spirometry and for whom there is reasonable equipoise about the most appropriate first line treatment regimen
2. *Primary outcome and endpoint:* The change in lung function 7–10 days after commencing intensive therapy (therapy entailing use of at least one intravenous antibiotic), measured as the absolute change in percentage predicted forced expiratory volume in 1-s (ppFEV_1_) from initiation of intensive therapy (measured closest in time to the first dose on IV therapy, *and not* ≥ 14 days before, *and not* ≥ 72 hours afterwards) to the first measured ppFEV_1_ ≥ 7 days afterwards (measured closest in time to 7x24 hours afterwards *but not* ≥ 10 days (10x24 hours) afterwards)
3. *Primary effect measure:* The absolute difference in the mean of the primary endpoint between those in the intervention versus the comparator group, adjusting for any differences in baseline factors between intervention and comparator arms that might otherwise confound or reduce the certainty of the treatment effect
4. *Interventions:* Prescription of each intervention within a domain
5. *Comparators*: Prescription of each other Intervention within that domain
6. *Intercurrent events:* Participants will be included in the primary analysis irrespective of whether and how the intervention or comparator is received, any non-adherence, any subsequent change in therapy, or loss to follow-up, (i.e., the effect attributable to the prescription, or the treatment policy or de facto estimand)

For each PERIT, we will also report the following outcomes measured as the following endpoints:

1. The change in lung function up to 6 months after commencement of intensive therapy, *measured as* the absolute change in the ppFEV_1_ from initiation of intensive therapy until approximately 14 days (≥ 14 days to < 30 days), 30 days (≥ 30 days to < 60 days), 60 days (≥ 60 days to < 90 days), and 180 days (≥ 180 days to < 240 days) after commencement of intensive therapy
2. The change in lung function up to 6 months after commencement of intensive therapy, *measured as: (i)* the proportional change (expressed as a percentage) in the ppFEV_1_ from initiation of intensive therapy until 7 days (≥ 7 to < 10 days), 14 days (≥ 14 days to < 30 days), 30 days (≥ 30 days to < 60 days), 60 days (≥ 60 days to < 90 days), and 180 days (≥ 180 days to < 240 days) after commencement of intensive therapy; (ii) the return of measured lung function to close to its baseline (pre-exacerbation) best function, up to 6 months after commencement of intensive therapy, *measured as* a binary outcome of whether the ppFEV_1_ has returned to ≥ 90% of the ppFEV_1_ at baseline, at approximately 7 days (≥ 7 to < 10 days), 14 days (≥ 14 days to < 30 days), 30 days (≥ 30 days to < 60 days), 60 days (≥ 60 days to < 90 days), and 180 days (≥ 180 days to < 240 days) after commencement of intensive therapy, where the baseline ppFEV_1_ is the highest recorded ppFEV1 in the 180 days (or 365 days if no ppFEV_1_ < 180 days) preceding the commencement of intensive therapy
3. The change in CF-related symptoms up to 4 weeks after commencement of intensive therapy, *measured as* the absolute change in the Chronic Respiratory Infection Symptom Score (CRISS) score approximately 7 days (≥ 7 to < 10 days), 14 days (≥ 14 days to < 30 days), 30 days (≥ 30 days to < 60 days), after commencement of intensive therapy
4. The time to the next PERIT, *measured as* the time (in days) from commencement of intensive therapy to the next commencement of intensive therapy, after a period of no intensive therapy of ≥ 7 days
5. Adverse reactions resulting in treatment cessation, *measured as* any early termination of planned therapy before 7 days (7*24 hours) and before 14 days (14*24 hours) after commencement of intensive therapy, owing to one or more adverse events presumed by the responsible clinician to be attributable to the prescribed therapy
6. Treatment failure resulting in treatment cessation, *measured as* any early termination of planned therapy before 7 days (7*24 hours) and before 14 days (14*24 hours) after commencement of intensive therapy, owing to inadequate improvement in symptoms or lung function, presumed by the responsible clinician to be attributable to a poor response to the prescribed therapy

Note that additional outcomes (including safety outcomes) may apply to cohort-nested sub-studies, and these will be detailed in their relevant subprotocols.

For all participants, we will also report:

1. The Cystic Fibrosis Questionnaire-revised (CFQR) score approximately every 12 weeks for all participants ≥ 6 years old
2. Any new detection of any strain of gram-negative bacteria with in vitro resistance to any aminoglycoside, fluoroquinolone, antipseudomonal penicillin (including beta-lactam/beta-lactamase inhibitor combination), antipseudomonal cephalosporin, or carbapenem not previously detected since enrolment in the PEx cohort or in the 2 years prior
3. Any new onset of *Clostridium difficile* associated diarrhea not previously detected since enrolment in the PEx cohort or in the 2 years prior

#### Participant timeline {13}

**Fig. 3:**
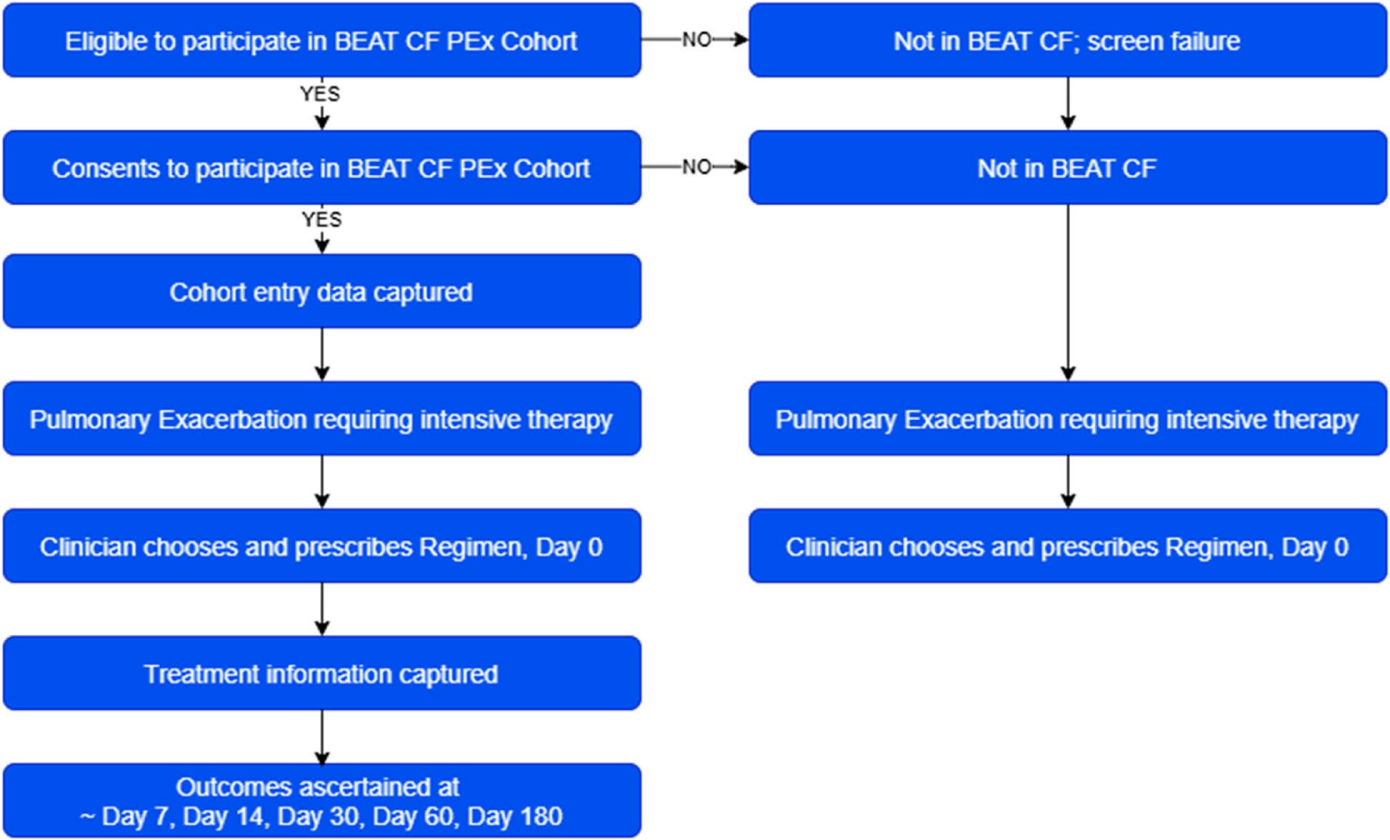
Enrolment and data collection timeline for PEx cohort participants

Once enrolled in the PEx Cohort, participants remain in the cohort until they withdraw, or until the project closes (see Criteria for Discontinuation above). The timing of individual PERITS varies between participants. Data collection occurs (1) at enrolment (cohort entry), (2) approximately every 3 months thereafter, and (3) with each PERIT (Fig. 3).

#### Sample size {14}

There is no maximum sample size for the PEx Cohort, although target or maximum sample sizes may apply to cohort-nested sub-studies, and these will be specified in their relevant subprotocols.

#### Recruitment {15}

A broad patient enrolment approach is taken with the aim of enrolling all willing and eligible people with CF. Potential screening avenues to identify eligible patients include:

1. During clinic appointments —potential participants may be directly approached during routine clinic appointments conducted by participating sites. Trained research staff will, after consulting with the clinical team, approach patients (or their parents) to discuss participation in the PEx cohort
2. Advertising material approved by the relevant Human Research Ethics Committee (HREC), including posters, brochures, and social media, may be used to promote and raise awareness of the project; these will appear in participating hospitals on notice boards or electronic notice boards. Advertisements may also be displayed in various community locations to assist enrolment
3. Letters—a HREC approved letter for distribution by participating sites to potential participants is supplied to all participating sites
4. BEAT CF website—a publicly accessible website where participants and their families can access the Participant Information Booklet, read updates about BEAT CF, and learn about the rationale and the team undertaking the research. This includes a section where interested people can receive further information or register their interest with contact details if they want to participate
5. Social media—the approved advertisements and other example promotional content is distributed through various social media avenues of the sponsor and supporting organizations, including Cystic Fibrosis Australia and its state branches

Research staff at participating sites are trained on project requirements prior to screening and enrolment of participants to ensure eligible participants are identified, educated about, and invited to participate in the PEx cohort. Enrolment and screening procedures occur according to the approved protocol and in line with the principles of Good Clinical Practice (GCP). Documented instructions for operational matters are provided to participating sites detailing enrolment, screening, and data collection procedures. Reasons for non-eligibility are recorded in a screening log.

### Assignment of interventions: allocation

#### Sequence generation {16a}

The PEx cohort does not entail any assignment of interventions, except as may be required by participation in any nested sub-studies. Methods for generating allocation sequences for those studies will be specified within the relevant subprotocols or their appendices.

#### Concealment mechanism {16b}

The PEx cohort does not entail any assignment of interventions, except as may be required by participation in any nested substudies. Methods for concealing the treatment assignment for those studies will be specified within the relevant subprotocols or their appendices.

#### Implementation {16c}

The PEx cohort does not entail any assignment of interventions, except as may be required by participation in any nested substudies. Methods for implementing the treatment assignment for those studies will be specified within the relevant subprotocols or their appendices.

### Assignment of interventions: blinding

#### Who will be blinded {17a}

The PEx cohort does not entail assignment of any interventions, except as may be required by participation in any nested sub-studies. Methods for implementing any blinding for those studies will be specified within the relevant sub-protocols or their appendices.

#### Procedure for unblinding if needed {17b}

Unblinding is not relevant for the PEx cohort. Where relevant, any procedures for unblinding for any nested sub-studies will be specified within the relevant subprotocols or their appendices.

### Data collection and management

#### Plans for assessment and collection of outcomes {18a}

PEx cohort data is collected on electronic and/or hard copy case report forms (eCRF/CRF) pertaining to (i) clinical and demographic data, (ii) disease characteristics (including pancreatic exocrine status, CF-related diabetes, genotype), (iii) anthropometric data (including height and weight), (iv) lung function, (v) PERITs including the treatment(s) received, serial lung function measurements at defined time points and laboratory and clinical outcomes, (vi) microbiological outcomes, and (vii) patient-reported outcomes. Tables 1 and 2 provide justifications for the collection of data in the PEx cohort, specific for individuals and PERITs, respectively. However, sub-studies may require the additional collection of these variables at different time points, which will be specified within the relevant subprotocols and appendices.

**Table 1:** Outcome data for BEAT-CF participants

Category	Variable	Timing		Justification
		At cohort entry	3-monthly	
**Demographic data**	Date of birth	Y		Age is independently associated with baseline lung function in CF. There may be “cohort” effects whereby those born in more recent periods have better outcomes because of improvements in care
	Sex	Y		Sex is independently associated with lung function in CF
**Clinical**	Genotype	Y		Validation of CF diagnosis
	Sweat test results	Y		Validation of CF diagnosis
	Pancreatic exocrine status	Y	Y	Pancreatic function is independently associated with lung function in CF
	CF-related diabetes	Y	Y	Diabetes is independently associated with lung function in CF
	Known drug allergies or contraindications	Y	Y	Drug allergies and contraindications constrain therapeutic options, and plausibly confounds or modifies treatment effects
	Concomitant medications	Y	Y	Use of concomitant medications plausibly confounds or modifies treatment effects, including toxicities
**Laboratory**	Total IgE	Y^a^	Y	Marker for allergic bronchopulmonary aspergillosis which is a cause of clinical deterioration and may confound PEx management
	*Aspergillus*-specific RAST	Y^a^	Y	Marker for allergic bronchopulmonary aspergillosis which is a cause of clinical deterioration and may confound PEx management
	Date and details of any new, documented infection, or airway colonization	Y^a^	Y	The presence of specific pathogens may influence therapeutic choices, plausibly confounds or modifies treatment effects. Selection of resistant pathogens may be an untoward outcome of antibiotic therapy
	Date and details of any new, documented infection with *Clostridium difficile*	Y^a^	Y	*Clostridium difficile*-related diarrhea is an important untoward outcome of antibiotic therapy
**Patient-reported outcomes**:	Cystic Fibrosis Questionnaire-revised (CFQ-R) for all participants ≥6 years old	Y	Y	Treatment of PEx plausibly impacts on quality-of-life (QoL) over time
**Anthropometric**	Height	Y	Y	Height is associated with FEV_1_ and is essential for estimating the ppFEV_1_
	Weight	Y	Y	Treatment of PEx plausibly impacts on weight over time
**Spirometry**	FEV_1_	Y^b^	Y	Treatment of PEx plausibly impacts on lung function. The ppFEV_1_, is strongly associated with morbidity and mortality in CF

^a^Including results in the 24 months preceding enrolment into the cohort

^b^Including results in the 12 months (365 days) preceding enrolment into the cohort

**Table 2:** Additional outcome data for each PERIT

	Variable	Timing	Reason
**PEx therapy**	Date and time of commencing IV therapy	Day 0	As a time reference point for analyses
**PEx therapy**	Location (hospital or HITH)	Daily	For description of the cohort. Location may constrain therapeutic options and plausibly confound or modify treatment effects
**Spirometry**	FEV_1_	Days 0 (at admission), 7, 14, 30, 60, and 180	Treatment of PEx plausibly impacts on lung function, measured as ppFEV_1_; this is strongly associated with morbidity and mortality in CF
**Clinical assessment**	Presence and quality of cough	Daily	Reduction in cough and/or improvement in cough quality are measures of successful treatment of PEx
	Presence of crepitations	Daily	Reduction in crepitations is a measure of successful treatment of PEx
**Patient-reported outcomes**	CRISS score (if > 12 years at admission)	Day 0: as close as practicable to the initiation of IV antibiotics, and not > 72 h afterwards, then days 7, 14, and 30	CRISS is a validated measure of CF-related symptoms in the preceding 24 h. It is expected that successful treatment will result in an improved CRISS score. It will be used to assess clinical response to treatment for PEx

Electronic patient-reported outcome (ePRO) tools are used to collect symptom and quality of life (QoL) data. These tools include:

1. Cystic Fibrosis Respiratory Symptoms Diary-Chronic Respiratory Infection Symptom Score (CFRSD-CRISS): a validated instrument in adults and children ≥ 12 years old which captures symptoms in the preceding 24 h [11]
2. CFQ-R: available in four versions (adults, adolescents, young children, and parents) in 34 languages and is a validated quality of life (QoL) instrument for use in adults and children > 6 years. This tool quantifies the impact of disease and treatment on overall health status and perceived well-being and includes an assessment of symptoms, treatment burden, and the integrity of relationships within the preceding 2 weeks [12]

#### Plans to promote participant retention and complete follow-up {18b}

The PEx cohort is designed to be pragmatic and embedded within routine care to foster high participation and retention and minimize the burden on participants and clinicians. Data quality and adherence to the protocol is promoted by (i) conducting a commencement meeting for all project coordinators and investigators at each site prior to their involvement; (ii) performing site inductions which include specific training about the protocol and the provision of reference documents for project staff; (iii) provision of a data dictionary to assist with data entry on the CRF/eCRF; and (iv) the data management center performs timely validation of data and addresses queries and corrections if errors are found during quality control checks.

#### Data management {19}

Streamlined data collection instruments and procedures are used to optimize efficiency and minimize the burden on participating sites. All data are entered into an eCRF by trained project staff. The eCRF is accessed via a web-based Clinical Trial Management System (CTMS). An Australian based company, Research Path, have been contracted to supply a web-based CTMS. The CTMS is a secure application comprising a web-based user interface and associated hosted services (backend) such as the web and database servers. All communication between the user’s browser and database servers is encrypted end-to-end. The core hosted servers (web, database, files) are all located in Australia, conform to the International Organisation for Standardisation requirements (ISO 27001), the Health Insurance Portability and Accountability Act (HIPAA), and Service Organisation Control standards (SOC 2), and employ strict infrastructure security policies. Access to all infrastructure systems and services is controlled through strict security controls and two-factor authentication. All users have permissions defined by their user role type as designated by the sponsor.

The project manager, or delegate, performs regular and timely validation of data, queries, and corrections. Any common patterns of errors found during quality control checks are fed back to participating sites. Missing data are minimized through regular data monitoring and a clear and comprehensive data dictionary with online data entry including logic checks.

As the PEx cohort is intended to be perpetual, data will be retained indefinitely. In the case of closure of the project, the sponsor will retain an identical replica of the platform database for at least 25 years or longer as is required by the approving regulatory authorities.

#### Confidentiality {27}

Participant data are identified by a unique study identification number on all study documentation, including the database, to ensure confidentiality. All documents are stored securely on the CTMS and are only accessible by project staff and authorized personnel. The project complies with all laws pertaining to data protection.

In order to administer the ePRO while ensuring participant anonymity, participant contact details are stored encrypted in a separate database segregated from the ePRO results. Minimum contact data is collected to ensure follow-up can be completed, including name, email address, and mobile phone. Electronic consent data, which are identifiable, are captured and stored separately to all other platform data in a secure database which is hosted by the sponsor.

#### Plans for collection, laboratory evaluation, and storage of biological specimens for genetic or molecular analysis in this trial/future use {33}

The PEx cohort entails no collection or storage of biological specimens, but collection or salvage may be required by participation in nested sub-studies. Where relevant, any procedures for collection or salvage of biological specimens for any nested studies will be specified within their relevant subprotocols or appendices.

### Statistical methods

#### Statistical methods for primary and secondary outcomes {20a}

Individual demographics, baseline clinical characteristics (at enrolment and at the commencement of each PERIT), outcomes, and loss to follow-up are periodically reported for all PEx cohort participants. The proportion of participants with the primary endpoint measured, and any loss to follow-up, is assessed and reported. Categorical variables are summarized at each level as a frequency and proportion. Cell frequencies below five members are reported as “< 5” to ensure individual confidentiality. Continuous variables are summarized as mean and standard deviation for symmetric distributions and median and interquartile range (IQR) for asymmetric distributions.

Continuous, categorical, and time-to-event endpoints may be assessed using standard statistical analytical approaches (chi squared, Fisher’s exact, Student *t* test, and Mann-Whitney tests as appropriate). Predictors of treatment outcomes will be assessed using Bayesian regression methods for binary, continuous, and time-to-event endpoints as appropriate. All summary statistics, analyses, and data visualizations will be generated in R version 3.5.3 or a later version, which is provided open source by the R Foundation for Statistical Computing. Analyses are performed and reported by the therapy received and the prespecified patient strata defined below; however, outcomes may not be reported for any Interventions assigned in the PEx Treatment Platform or other sub-studies if this information would inform or impact ongoing comparative effectiveness evaluations. All other subgroup analyses will be reported as post hoc.

#### Interim analyses {21b}

Descriptive summaries of the PEx cohort and analyses will be periodically updated and reported. Any interim or scheduled analyses for any nested sub-studies will be specified a priori in their relevant subprotocols and appendices.

#### Methods for additional analyses (e.g., subgroup analyses) {20b}

The pre-specified strata for primary and secondary analyses for the PEx cohort are:

1. ppFEV_1_ ≥ 70% in the preceding 12 months (365 days) or unable to perform spirometry (e.g., due to young age) and no known *Pseudomonas aeruginosa* colonization in the preceding 2 years (2*365 days)
2. ppFEV_1_ ≥ 70% in the preceding 12 months (365 days) or unable to perform spirometry (e.g., due to young age) and known *Pseudomonas aeruginosa* colonization in the preceding 2 years (2*365 days).
3. ppFEV_1_ < 70% in the preceding 12 months (365 days) and no known *Pseudomonas aeruginosa* colonization in the preceding 2 years (2*365 days)
4. ppFEV_1_ < 70% in the preceding 12 months (365 days) and known *Pseudomonas aeruginosa* colonization in the preceding 2 years (2*365 days)

The number of strata within PEx cohort may be prospectively updated, depending on the impact of such decisions on precision. Other subgroup analyses may be performed and will be reported as post hoc.

#### Methods in analysis to handle protocol non-adherence and any statistical methods to handle missing data {20c}

In the primary analyses, participants will be assessed according to the initially prescribed treatment, irrespective of intercurrent events, including whether and how treatments are received, non-adherence, changes to therapy, or loss to follow-up, i.e., the analysis will determine the effect attributable to the initial treatment plan, reflecting the de facto or “treatment policy” estimand.

#### Plans to give access to the full protocol, participant level-data and statistical code {31c}

The current version of the full PEx Core Protocol will be accessible on the project website beatcf.org.au. Decisions regarding the sharing of de-identified data and/or statistical code will be assessed by the project Steering Committee and will be conditional upon any necessary institutional and ethics approvals and data sharing agreements.

### Oversight and monitoring

#### Composition of the coordinating center and trial steering committee {5d}

Please refer to *S1* for an overview of the BEAT CF administrative structure. The scope of responsibility for individual groups that operate within this framework (e.g., Steering Committee, Executive Committee and Operations Group) are outlined in their terms of reference (S1).

As sponsor, the University of Sydney assumes overall responsibility for the PEx cohort, including future nested sub-studies, but will largely delegate study-specific duties to the Steering Committee, which will comprise the coordinating principal investigator (CPI), representatives of the investigator and community reference groups, and invited representatives of key clinical stakeholders. There are two separate community reference groups: one for adults and young people with CF, and one for parents, partners, and carers of people with CF.

#### Composition of the data monitoring committee, its role and reporting structure {21a}

There will be no independent safety monitoring for the PEx cohort, except where required for any nested sub-studies. The composition and role of the BEAT CF Data Safety Monitoring Committee will be detailed in the relevant subprotocol (s) or their appendices.

#### Adverse event reporting and harms {22}

Adverse events will not be ascertained or reported for the PEx Cohort, only the solicited safety outcomes detailed above. Where relevant, any procedures for ascertaining adverse events for any nested sub-studies will be specified within the relevant subprotocols or their appendices.

#### Frequency and plans for auditing trial conduct {23}

Monitoring will be performed according to the International Committee for Harmonisation (ICH) guidelines, in particular E6 (also known as Good Clinical Practice (GCP). Data will be evaluated for compliance with the protocol and accuracy in relation to source documents. Following written operating procedures, monitors will verify that for the PEx Cohort (including any nested sub-studies), data are generated, documented, and reported in compliance with the protocol, GCP, applicable regulatory requirements.

A risk-based approach has been used to develop a monitoring plan. Risk-based monitoring differs from conventional monitoring as it prioritizes specific risks for this project as opposed to conducting routine site visits with complete source data verification. This has identified and defined the data and processes critical to data quality and the processes required to minimize them. Quality indicators and thresholds that would trigger an investigation and/or corrective action have been set and documented in a Data Monitoring Plan.

Obligations expected of sites to assist the sponsor in monitoring the study may include hosting site visits, providing information for remote monitoring, or putting procedures in place to monitor internally. Any additional processes for defining, assessing, reporting, and monitoring nested sub-studies will be outlined in their relevant subprotocols.

#### Plans for communicating important protocol amendments to relevant parties (e.g., trial participants, ethical committees) {25}

Any substantial amendment to the original approved PEx core protocol or its subprotocols and appendices will require prior approval by all relevant ethical and regulatory review bodies. Amendments that are not substantial will not be notified to such review bodies but will be recorded and filed by the sponsor.

#### Dissemination plans {31a}

The results will be communicated by presentation and publication. The two CRGs will provide guidance on the best methods of dissemination of information to participants and the wider CF community. The Steering Committee will coordinate dissemination of data (including from any nested sub-studies), endeavoring to make any conclusions broadly available to the CF community as soon as possible. The Steering Committee will, as far as possible, make the protocol(s), statistical analysis plans, and participant-level data available in order to allow independent scientific scrutiny and validation of any published results. All investigators will have the opportunity to review publications (e.g., manuscripts, abstracts, oral/slide presentations, book chapters) prior to submission. Authorship will be determined in line with the uniform requirements for manuscripts submitted to biomedical journals published by the International Committee of Medical Journal Editors. Any additional processes or requirements for reporting the results of nested sub-studies will be outlined in their relevant subprotocols.

## Discussion

The BEAT CF PEx core protocol sets out a prospective cohort designed to perpetually enroll people with CF to evaluate the comparative effectiveness of interventions used for managing PEx. It is intended that sub-studies will be nested within this cohort in order to generate high-quality evidence to optimize the management of PEx.

The primary endpoint is the absolute change in ppFEV_1_ approximately 7 days after initiation of intensive therapy. FEV1 is a practical and objective endpoint for PEx which has been shown to correlate with quality of life, mortality and with structural lung changes, making it the most widely used clinical trial endpoint in CF [13]. Increasingly spirometry can be performed in the home using calibrated personal spirometry devices, meaning it may not be necessary for assessments to be performed in hospital. We have selected a short-term primary endpoint to inform guidance on treatment decisions in the first week of intensive therapy, even though other trials have typically assessed responses to treatment after several weeks. CF physicians often change therapy if there is unsatisfactory clinical response after 7–10 days [14], so measuring the primary outcome at a later time point will introduce an unacceptable amount of treatment crossover that will complicate interpretation of the results. Published data indicate that most of the improvement in FEV_1_ following treatment for exacerbations occurs within the first week of intensive therapy [15], and the short-term change in FEV_1_ is strongly predictive of the longer term change in FEV_1_ [16]. From a practical perspective, most CF centers re-check the FEV_1_ after 7–10 days or have opportunity to do so, and a recent trial found evidence that it is acceptable to curtail antibiotic treatment to just 10 days of intravenous therapy in those with an acceptable FEV_1_ and symptom response after 7 days of therapy [17]. In summary, measurement of ppFEV_1_ as the primary endpoint at a timepoint where CF physicians typically measure FEV_1_ and make decisions about future treatment allows a robust outcome measure that will be clinically informative.

The decision was made for the primary outcome to be absolute rather than relative change in the ppFEV_1_ for two reasons. Firstly, mean absolute change in FEV_1_ has been shown to have lower variability than relative change [18]. Secondly, relative changes in FEV1 are non-symmetric for increasing versus decreasing changes. For example, if half of participants experience a change in ppFEV_1_ from 50 to 100 (+100% increase), and the other half experience a change from 100 to 50 (− 50% change) the net change in ppFEV_1_ will be + 25% (i.e., (− 50% + 100%)/2 = 25%), rather than zero.

Importantly, additional endpoints will be captured which include safety, effects on microbiology, and more patient-centered outcomes such as the CRISS (symptom score) which provides information that is complementary to lung function measurements and which captures different aspects of clinical response [18].

There is no consensus regarding the optimal endpoints for children with CF who are too young to perform spirometry [13]. Lung clearance index (LCI) [19] and structural lung disease captured by radiological imaging have been proposed as alternative outcome measures in this age group [20]. LCI in children < 6 years old has been shown to correlate with abnormal lung function and structural lung disease at school age entry [21]. Computerized tomography (CT) scores have been found to correlate with clinical status, lung function and disease progression and are predictive of mortality [22]. However, use of LCI and radiological imaging such as CT or magnetic resonance imaging (MRI) are currently restricted to the research context, because these investigations are not routinely performed, and interpretation of their results lacks standardization [22]. While these outcome measures are not included as part of the proposed data capture for BEAT CF, they may be considered for inclusion in the future.

No clear consensus exists around the definition of PEx of CF and definitions that might fit adult patients do not necessarily fit children [23]. However, a clear association exists between the decision to prescribe intravenous antibiotics for treatment of pulmonary symptoms or signs and health outcomes. Hence, a clinician’s decision to treat with intravenous antibiotics is often used in clinical trials to define exacerbations [1, 23]. BEAT CF will therefore focus on pulmonary exacerbations requiring intensive therapy (PERIT) defined pragmatically as therapy comprising the initiation of at least one antibiotic by the intravenous route. This definition may need to be re-visited if evidence emerges for use of oral or inhaled antibiotic therapies without intravenous antibiotics.

We have followed the latest ICH statistical guidance (E9 (R1)) to specify analyses in the estimand framework [24]. In the estimand framework, each analysis needs to be explicit about the target of (causal) inference. At a minimum, this requires specification of (1) the analysis population (who is included and excluded?), (2) the outcome (what part of the patient experience are we trying to capture?), (3) the endpoint (what are we using as a valid measure of that outcome?), (4) the intervention (e.g., what treatment, range of doses, frequencies, durations can be considered to constitute the treatment in question?), (5) the comparator (e.g., what treatment or range of treatments, their doses, frequencies), (6) the summary of the treatment effect (how are we framing the contrast between investigational intervention and its comparator? e.g., relative risk, odds ratio, etc.), (7) the approach to handling intercurrent events (how will treatment non-completion and crossovers, loss-to-follow-up, missing endpoints, deaths, etc., be managed?). Here, we have prioritized the de facto estimand over the de jure estimand, meaning that we are implicitly prioritizing measurement of the effect of prescribing an intervention (or the “intention-to-treat”), over the effect of adhering to the prescribed intervention. By focusing on short-term endpoints after largely supervised therapy, we anticipate that the de facto estimand will approximate the de jure estimand, although we expect to observe some early treatment discontinuations and crossovers due to adverse reactions.

As well as establishing a prospective observational cohort, the PEx core protocol will serve as the highest tier of a “master protocol” for the nesting of clinical trials and other sub-studies within the cohort. The concept of the master protocol has evolved over the past decade, alongside growing interest in complex study designs like basket, umbrella, and platform trials [25]. The United States Food and Drug Administration use master protocol to mean a protocol for one of these complex designs comprising either the evaluation of multiple therapies in a single patient group (umbrella trial), a single therapy in multiple patient groups (basket trial), or multiple therapies in multiple patient groups or subgroups (adaptive platform trial). They have recently released a guidance document for industry for master protocols for late-stage evaluation of therapeutics in oncology, which has seen by far the greatest implementation of these designs [26]. The value of master protocol trial designs is that they foster sharing of resources across what would traditionally be disparate trials and enforce standardization of data collection, trial endpoints, and analyses.

We have employed a modular and hierarchical approach to the protocol documentation, borrowing from the approach and nomenclature of REMAP-CAP, which is primarily an adaptive platform trial with a core protocol, with a series of appendices for each treatment domain [27]; for REMAP-CAP, the core protocol sets out the primary objectives and outcomes as well as all processes which are generic to the platform trial including randomization, while the domain-specific appendices specify the exact interventions in each domain, and any other domain or treatment specific processes. We deviate from the REMAP-CAP design in that the PEx core protocol primarily describes an observational cohort (rather than a trial), defining its core objectives and primary outcomes and endpoints which will be generic to all nested sub-studies; our intention is that any secondary objectives or outcomes which are specific to nested sub-studies, along with any sub-study specific procedures (including randomization and safety reporting) will be detailed in their specific subprotocols and/or appendices. In this way, our approach follows the concept of nesting trials within cohorts or registries, which has been advocated and occasionally implemented [28, 29].

As well as creating a pool for identifying study participants, the value of nesting studies within an existing large cohort or registry is that it reduces the incremental cost and burden of data capture and allows investigators to assess how representative study participants are of the patient population and therefore how generalizable any findings are likely to be. It is intended that most PEx cohort participants will become eligible to participate in a PEx Treatment Platform in which selected aspects of PERIT management will be randomly assigned, as well as other cohort-nested sub-studies to address secondary research aims. The details for the PEx Treatment Platform and any nested sub-studies will be defined separately in subprotocols.

We have presented the PEx core protocol here using the SPIRIT checklist for clinical trials [30]. Although this checklist is not designed for observational cohorts, we expect that adhering to this reporting standard will facilitate the nesting of subprotocols, including those for cohort-nested clinical trials, which will supplement this protocol with additional information under the same headings.

The PEx core protocol (and any nested sub-studies) is designed to support embedding of study procedures in routine clinical care. Embedding not only reduces the costs and burdens of research, but it is also intended to capture the real-world use of therapy, arguably making any findings more relevant for decision-making. Our vision is a ‘learning health system’ approach to CF management, collecting data from pulmonary exacerbations to generate and rapidly translate insights from analysis into patient care. To this end, the project is overseen by a central steering committee which comprises representatives from the project investigators, the community reference groups, and stakeholders from the clinical craft groups.

## Trial status

Current Protocol version 7.0 (8 November 2021). Recruitment commencement date: 14 October 2020

## Supplementary Information

**Additional file 1: S1.** BEAT CF administration structure.

**Additional file 2: ** Beatcf data dictionary.

## Data Availability

Access to data will be granted to authorized representatives from the steering committee, sponsor, host institution, and the regulatory authorities to permit trial-related monitoring, audits, and inspections. BEAT CF will comply with all relevant jurisdictional and academic requirements relating to access to data, as apply at the time that the data are generated. Ownership and access to data where a commercial organization is involved (e.g., by provision of goods or services that are tested within a domain) will be set out in a contract between sponsor and that commercial organization.

## Abbreviations

CAHS: Child and Adolescent Health Service
CF: Cystic fibrosis
CFQR: Cystic Fibrosis Questionnaire Revised
CI: Chief investigator
CPI: Coordinating principal investigator
CRF: Case report form
CRG: Consumer reference group
CRISS™: Chronic Respiratory Infections Symptom Score
CTMS: Clinical Trial Management System
DSA: Domain-Specific Appendix
DSMB: Data Safety and Monitoring Board
eCRF: Electronic case report form
ePRO: Electronic patient-reported outcomes
FEV_1_: Forced expiratory volume in one second
GCP: Good Clinical Practice
GP: General practitioner
HREC: Human Research Ethics Committee
IQR: Interquartile range
IT: Information technology
ITT: Intention to treat
NHMRC: National Health and Medical Research Council
PEx: Pulmonary exacerbation
PERIT: Pulmonary exacerbation requiring intensive therapy
QoL: Quality of life
RAR: Response adaptive randomization
REMAP: Randomised Embedded Multifactorial Adaptive Platform
SOP: Standard operating procedures
TFA: Two-factor authentication
TGA: Therapeutic Goods Administration
TKI: Telethon Kids Institute
WHO: World Health Organization
