# Supplementary figures and images for: BEAT CF pulmonary exacerbations core protocol for evaluating the management of pulmonary exacerbations in people with cystic fibrosis
Source: Trials. 2023 Mar 22;24:211. doi: 10.1186/s13063-023-07076-8 (PMC10031862; doi:10.1186/s13063-023-07076-8)

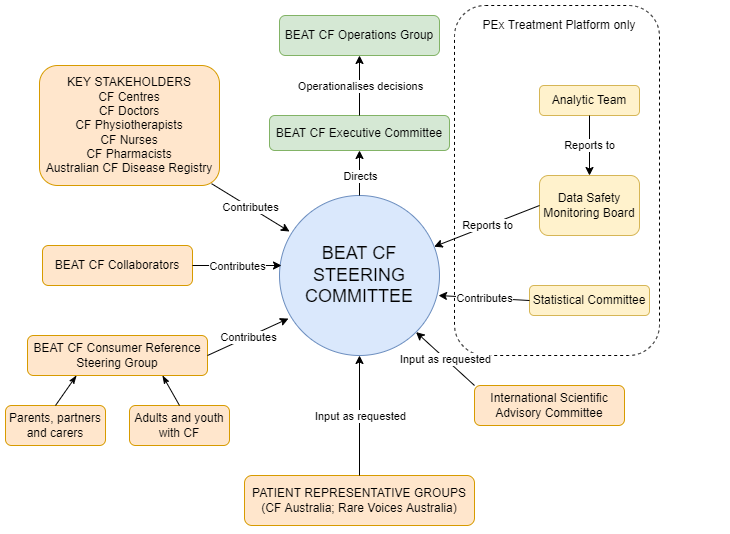

Supplement: Supplementary file 1 — Additional file 1: S1. BEAT CF administration structure. [file 13063_2023_7076_MOESM1_ESM.tif]
