# Supplementary material for: BEAT CF pulmonary exacerbations core protocol for evaluating the management of pulmonary exacerbations in people with cystic fibrosis
Source: Trials. 2023 Mar 22;24:211. doi: 10.1186/s13063-023-07076-8 (PMC10031862; doi:10.1186/s13063-023-07076-8)
Supplement: Supplementary file 2 — Additional file 2: Beatcf data dictionary. [file 13063_2023_7076_MOESM2_ESM.pdf]

form_number	form_name	number	name	prompt	description	type	units	default	values
1	Baseline	1.1	genotype1	Genotype (Mutation 1)		option			1 = F508del--- p.Phe508del--- c.1521_1523delCTT<br>2 = G542X ---p.Gly542X ---- c.1624G>T<br>3 = G551D--- p.Gly551Asp --- c.1652G>A<br>4 = N1303K--- p.Asn1303Lys --- c.3909C>G<br>5 = W1282X--- p.Trp1282X --- c.3846G>A---<br>6 = R117H;5T--- p.Arg117His;None(5T) --- c.[350G>A<br>7 = R117H;7T--- p.Arg117His;None(7T) --- c.[350G>A<br>8 = R117H--- p.Arg117His --- c.350G>A<br>9 = R553X ---p.Arg553X --- c.1657C>T<br>10 = 1717-1G->A--- None --- c.1585-1G>A<br>11 = 621+1G->T--- None ---c.489+1G>T<br>12 = 2789+5G->A--- None --- c.2657+5G>A<br>13 = 3849+10kbC->T--- None--- c.3717+12191C>T<br>14 = R1162X--- p.Arg1162X --- c.3484C>T<br>15 = 2183AA->G or 2183delAA->G--- p.Lys684Serfs)<br>16 = CFTRdele2 3 ---p.Ser18ArgfsX16 ---c.54-5940_<br>17 = G85E--- p.Gly85Glu ---- c.254G>A<br>18 = 3120+1G->A--- None--- c.2988+1G>A<br>19 = I507del ---p.Ile507del --- c.1519_1521delATC<br>20 = 1898+1G->A ---None--- c.1766+1G>A<br>21 = 3659delC--- p.Lys1177SerfsX15 --- c.3528delC<br>22 = R347P ---p.Arg347Pro --- c.1040G>C<br>23 = D1152H ---p.Asp1152His --- c.3454G>C<br>24 = R560T ---p.Arg560Thr --- c.1679G>C<br>25 = 3272-26A->G ---None ---c.3140-26A>G<br>26 = Q493X ---p.Gln493X --- c.1477C>T<br>27 = E60X--- p.Glu60X --- c.178G>T<br>28 = R334W ---p.Arg334Trp ---c.1000C>T<br>29 = 394delTT--- p.Leu88IlefsX22 --- c.262_263delTT<br>30 = 2184insA--- p.Gln685ThrfsX4 --- c.2052_2053in<br>31 = 5T;TG11--- None--- c.[1210-12[5];1210-34TG[1<br>32 = 5T;TG12--- None ---c.[1210-12[5];1210-34TG[1<br>33 = 5T;TG13 ---None--- c.[1210-12[5];1210-34TG[1<br>34 = 5T;TG not specified--- None--- c.1210-12[5]<br>35 = 3905insT--- p.Leu1258PhefsX7 --- c.3773_3774<br>36 = Y1092X--- p.Tyr1092X --- c.3276C>A<br>37 = Y1092X ---p.Tyr1092X --- c.3276C>G<br>38 = Y1092X--- p.Tyr1092X --- cDNA variant not spec<br>39 = A455E--- p.Ala455Glu --- c.1364C>A<br>40 = 2184delA--- p.Lys684AsnfsX38 --- c.2052delA<br>41 = R1066C--- p.Arg1066Cys --- c.3196C>T<br>42 = 1078delT--- p.Phe316LeufsX12 --- c.948delT<br>43 = 1154insTC--- p.Phe342HisfsX28 --- c.1022_102<br>44 = R1158X--- p.Arg1158X ---c.3472C>T<br>45 = R347H --- p.Arg347His --- c.1040G>A<br>46 = S1251N --- p.Ser1251Asn --- c.3752G>A<br>47 = L206W--- p.Leu206Trp --- c.617T>G<br>48 = S549N--- p.Ser549Asn --- c.1646G>A<br>49 = M1101K--- p.Met1101Lys --- c.3302T>A<br>50 = 711+1G->T--- None---- c.579+1G>T<br>51 = Y122X--- p.Tyr122X --- c.366T>A<br>52 = 2143delT--- p.Leu671X--- c.2012delT<br>53 = S945L--- p.Ser945Leu --- c.2834C>T<br>54 = I148T--- p.Ile148Thr --- c.443T>C<br>55 = R117C--- p.Arg117Cys --- c.349C>T<br>56 = V520F ---p.Val520Phe --- c.1558G>T<br>57 = S1235R --- p.Ser1235Arg --- c.3705T>G<br>58 = T338I--- p.Thr338Ile --- c.1013C>T<br>59 = P67L--- p.Pro67Leu --- c.200C>T<br>60 = G1244E ---p.Gly1244Glu ---c.3731G>A<br>61 = G178R--- p.Gly178Arg --- c.532G>A<br>62 = 1677delTA --- p.Tyr515X --- c.1545_1546delTA<br>63 = R352Q --- p.Arg352Gln --- c.1055G>A<br>64 = 711+5G->A --- None --- c.579+5G>A<br>65 = R668C --- p.Arg668Cys --- c.2002C>T<br>66 = S549R --- p.Ser549Arg --- c.1645A>C<br>67 = S549R ---p.Ser549Arg --- c.1647T>G<br>68 = S549R ---p.Ser549Arg ---cDNA variant not spe<br>69 = A559T--- p.Ala559Thr --- c.1675G>A<br>70 = L1077P--- p.Leu1077Pro ---c.3230T>C<br>71 = W1089X--- p.Trp1089X --- c.3266G>A<br>72 = I1027T--- p.Ile1027Thr --- c.3080T>C<br>73 = G576A ---p.Gly576Ala --- c.1727G>C<br>74 = M470V ---p.Met470Val --- c.1408A>G<br>75 = 3120G->A--- None --- c.2988G>A<br>76 = R75X --- p.Arg75X --- c.223C>T<br>77 = W846X --- p.Trp846X --- c.2537G>A<br>78 = E585X --- p.Glu585X --- c.1753G>T<br>79 = 1811+1.6kbA->G--- None --- c.1679+1.6kbA>G<br>80 = 3876delA--- p.Lys1250ArgfsX9 --- c.3744delA<br>81 = D1270N --- p.Asp1270Asn ---c.3808G>A<br>82 = Q220X ---p.Gln220X ---c.658C>T<br>83 = 2307insA--- p.Glu726ArgfsX4 --- c.2175_2176in<br>84 = D110H ---p.Asp110His --- c.328G>C<br>85 = 4016insT--- p.Ser1297PhefsX5 --- c.3884_3885i<br>86 = 4382delA--- p.Glu1418ArgfsX14 --- c.4251delA<br>87 = I336K ---p.Ile336Lys ---c.1007T>A<br>88 = R1066H--- p.Arg1066His --- c.3197G>A<br>89 = 2347delG--- p.Val739TyrfsX16 --- c.2215delG<br>90 = L997F ---p.Leu997Phe --- c.2991G>C<br>91 = K710X--- p.Lys710X --- c.2128A>T<br>92 = E822X ---p.Glu822X --- c.2464G>T<br>93 = L1065P ---p.Leu1065Pro --- c.3194T>C<br>94 = Q552X ---p.Gln552X ---c.1654C>T<br>95 = R74W--- p.Arg74Trp --- c.220C>T<br>96 = 2622+1G->A--- None --- c.2490+1G>A<br>97 = 2789+2insA--- None--- c.2657+2_2657+3insA<br>98 = E92X--- p.Glu92X --- c.274G>T<br>99 = Q39X--- p.Gln39X --- c.115C>T<br>100 = R75Q--- p.Arg75Gln --- c.224G>A<br>101 = D579G--- p.Asp579Gly --- c.1736A>G<br>102 = E831X--- p.Glu831X --- c.2491G>T<br>103 = 3007delG --- p.Ala959HisfsX9 --- c.2875delG<br>104 = 405+1G->A --- None ---c.273+1G>A<br>105 = 406-1G->A--- None ---c.274-1G>A<br>106 = 711+3A->G--- None--- c.579+3A>G<br>107 = Q1313X--- p.Gln1313X --- c.3937C>T<br>108 = R709X--- p.Arg709X --- c.2125C>T<br>109 = 2711delT--- p.Phe861LeufsX3 --- c.2583delT<br>110 = 4005+1G->A--- None --- c.3873+1G>A<br>111 = 574delA--- p.Ile148LeufsX5 --- c.442delA<br>112 = 1525-1G->A--- None--- c.1393-1G>A<br>113 = 1812-1G->A--- None--- c.1680-1G>A<br>114 = I1234V ---p.Ile1234Val --- c.3700A>G<br>115 = R1070Q--- p.Arg1070Gln--- c.3209G>A<br>116 = S466X ---p.Ser466X --- c.1397C>A<br>117 = S466X--- p.Ser466X --- c.1397C>G<br>118 = S466X--- p.Ser466X --- cDNA variant not spec<br>119 = S489X--- p.Ser489X --- c.1466C>A<br>120 = L467P--- p.Leu467Pro --- c.1400T>C<br>121 = S492F--- p.Ser492Phe --- c.1475C>T<br>122 = 3791delC--- p.Thr1220LysfsX8 --- c.3659delC<br>123 = L927P--- p.Leu927Pro --- c.2780T>C<br>124 = 712-1G->T--- None--- c.580-1G>T<br>125 = E92K--- p.Glu92Lys --- c.274G>A<br>126 = Q359K/T360K--- p.[Gln359Lys;Thr360Lys] --- c<br>127 = Q890X ---p.Gln890X --- c.2668C>T<br>128 = R764X ---p.Arg764X --- c.2290C>T<br>129 = S1196X ---p.Ser1196X --- c.3587C>G<br>130 = W401X--- p.Trp401X --- c.1202G>A<br>131 = W401X ---p.Trp401X --- c.1203G>A<br>132 = W401X ---p.Trp401X --- cDNA variant not spec<br>133 = 1248+1G->A--- None--- c.1116+1G>A<br>134 = L732X--- p.Leu732X ---c.2195T>G<br>135 = Q98X--- p.Gln98X --- c.292C>T<br>136 = R1070W--- p.Arg1070Trp--- c.3208C>T<br>137 = R31C--- p.Arg31Cys --- c.91C>T<br>138 = R851X ---p.Arg851X ---c.2551C>T<br>139 = W1204X--- p.Trp1204X --- c.3611G>A<br>140 = W1204X--- p.Trp1204X --- c.3612G>A<br>141 = W1204X--- p.Trp1204X --- cDNA variant not sp<br>142 = 663delT--- p.Ile177MetfsX12 --- c.531delT<br>143 = F1052V--- p.Phe1052Val--- c.3154T>G<br>144 = G330X ---p.Gly330X --- c.988G>T<br>145 = P205S--- p.Pro205Ser --- c.613C>T<br>146 = 1259insA--- p.Gln378AlafsX4 --- c.1127_1128i<br>147 = 2585delT--- p.Leu818TrpfsX3 --- c.2453delT<br>148 = 852del22--- p.Gly241GlufsX13 --- c.720_741de<br>149 = E1104X ---p.Glu1104X --- c.3310G>T<br>150 = H199Y ---p.His199Tyr --- c.595C>T<br>151 = Q525X ---p.Gln525X --- c.1573C>T<br>152 = 1461ins4--- p.Ile444ArgfsX3 --- c.1329_1330in<br>153 = 1898+3A->G--- None--- c.1766+3A>G<br>154 = 7T--- None--- c.1210-12[7]<br>155 = CFTRdele22 23 ---None ---c.3964-78_4242+5<br>156 = D614G--- p.Asp614Gly --- c.1841A>G<br>157 = L227R--- p.Leu227Arg ---c.680T>G<br>158 = L558S--- p.Leu558Ser --- c.1673T>C<br>159 = 1213delT--- p.Trp361GlyfsX8 --- c.1081delT<br>160 = 1341+1G->A--- None--- c.1209+1G>A<br>161 = 1548delG--- p.Gly473GlufsX54 --- c.1418delG<br>162 = 1717-8G->A--- None--- c.1585-8G>A<br>163 = 3121-1G->A--- None--- c.2989-1G>A<br>164 = 4209TGTT->AA--- None--- c.4077_4080delTG<br>165 = 457TAT->G--- p.Tyr109GlyfsX4 --- c.325_327de<br>166 = G1069R ---p.Gly1069Arg --- c.3205G>A<br>167 = G970R--- p.Gly970Arg--- c.2908G>C<br>168 = M1V--- p.Met1Val --- c.1A>G<br>169 = R1162L--- p.Arg1162Leu --- c.3485G>T<br>170 = R560K--- p.Arg560Lys --- c.1679G>A<br>171 = S341P--- p.Ser341Pro --- c.1021T>C<br>172 = S977F--- p.Ser977Phe --- c.2930C>T<br>173 = V754M ---p.Val754Met--- c.2260G>A<br>174 = Y569D ---p.Tyr569Asp ---c.1705T>G<br>175 = 2594delGT ---p.Ser821ArgfsX4 --- c.2462_246<br>176 = 4374+1G->T--- None--- c.4242+1G>T<br>177 = R785X--- p.Arg785X --- c.2353C>T<br>178 = 2055del9->A--- p.Ser641ArgfsX5--- c.1923_1<br>179 = 2105-2117del13insAGAAA--- p.Arg658LysfsX4<br>180 = G551S--- p.Gly551Ser --- c.1651G>A<br>181 = A561E--- p.Ala561Glu --- c.1682C>A<br>182 = H1054D--- p.His1054Asp --- c.3160C>G<br>183 = 1288insTA--- p.Asn386IlefsX3 --- c.1153_1154<br>184 = 1471delA--- p.Lys447ArgfsX2 --- c.1340delA<br>185 = 444delA--- p.Ile105SerfsX2--- c.313delA<br>186 = 3667ins4--- p.Thr1179IlefsX17 --- c.3535_3536<br>187 = 3821delT--- p.Ser1231ProfsX4 --- c.3691delT<br>188 = 4326delTC--- p.Cys1400X --- c.4196_4197del<br>189 = R792X--- p.Arg792X --- c.2374C>T<br>190 = S912X--- p.Ser912X --- c.2735C>A<br>191 = C276X ---p.Cys276X --- c.828C>A<br>192 = 1811+1G->C--- None--- c.1679+1G>C<br>193 = 3850-1G->A ---None ---c.3718-1G>A<br>194 = 306insA ---p.Arg59LysfsX10 --- c.174_175insA<br>195 = A46D ---p.Ala46Asp --- c.137C>A<br>196 = 1782delA--- p.Gly551ValfsX8 --- c.1650delA<br>197 = 2118del4--- p.Thr663ArgfsX8 --- c.1986_1989<br>198 = 2869insG--- p.Tyr913X --- c.2737_2738insG<br>199 = 2896insAG--- p.Val922GlufsX2 --- c.2764_2765<br>200 = 3132delTG--- p.Val1001AspfsX45 --- c.3002_3<br>201 = 3737delA--- p.Asp1202AlafsX9 --- c.3605delA<br>202 = Q414X--- p.Gln414X --- c.1240C>T<br>203 = S1255X--- p.Ser1255X --- c.3764C>A<br>204 = E56K--- p.Glu56Lys ---c.166G>A<br>205 = G1061R--- p.Gly1061Arg--- c.3181G>C<br>206 = F1074L ---p.Phe1074Leu --- c.3222T>A<br>207 = G1349D--- p.Gly1349Asp --- c.4046G>A<br>208 = 4428insGA--- p.Ser1435GlyfsX14 --- c.4296_4<br>209 = Y849X--- p.Tyr849X --- c.2547C>A<br>210 = E1371X ---p.Glu1371X--- c.4111G>T<br>211 = 1898+1G->C--- None ---c.1766+1G>C<br>212 = 2790-1G->C--- None--- c.2658-1G>C<br>213 = 297-1G->A--- None--- c.165-1G>A<br>214 = 1824delA--- p.Asp565MetfsX7 --- c.1692delA<br>215 = 2556insAT--- p.Ser809IlefsX13 --- c.2424_2425<br>216 = 3121-977_3499+248del2515--- None--- c.2989<br>217 = 4015delA--- p.Ile1295PhefsX33--- c.3883delA<br>218 = 675del4--- p.Leu183PhefsX5 --- c.543_546del<br>219 = Y913X--- p.Tyr913X --- c.2739T>A<br>220 = Q1412X--- p.Gln1412X --- c.4234C>T<br>221 = S1255P--- p.Ser1255Pro --- c.3763T>C<br>222 = CFTRdele17a-18--- None--- c.(2988+1_2989-1<br>223 = CFTRdele2--- None--- c.(53+1_54-1)_(164+1_<br>224 = CFTRdele22-24 ---None--- c.(3963+1_3964-1)<br>225 = L1254X ---p.Leu1254X--- c.3761T>G<br>226 = 1138insG--- p.Ile336SerfsX28--- c.1006_1007i<br>227 = 935delA--- p.Asn268IlefsX17--- c.803delA<br>228 = 1161delC--- p.Cys343X--- c.1029delC<br>229 = 1609delCA ---p.Gln493ValfsX10--- c.1477_147<br>230 = CFTRdele14b-17b--- None ---c.(2619+1_2620<br>231 = CFTRdele17a 17b--- None--- c.(2988+1_2989-<br>232 = E193X ---p.Glu193X--- c.577G>T<br>[illegible]

1	Baseline	1.1.1	genospec1	Specify other Genotype		string			

1	Baseline	1.2	genotype2	Genotype (Mutation 2)		option			1 = F508del--- p.Phe508del--- c.1521_1523delCTT<br>2 = G542X ---p.Gly542X ---- c.1624G>T<br>3 = G551D--- p.Gly551Asp --- c.1652G>A<br>4 = N1303K--- p.Asn1303Lys --- c.3909C>G<br>5 = W1282X--- p.Trp1282X --- c.3846G>A---<br>6 = R117H;5T--- p.Arg117His;None(5T) --- c.[350G>A<br>7 = R117H;7T--- p.Arg117His;None(7T) --- c.[350G>A<br>8 = R117H--- p.Arg117His --- c.350G>A<br>9 = R553X ---p.Arg553X --- c.1657C>T<br>10 = 1717-1G->A--- None --- c.1585-1G>A<br>11 = 621+1G->T--- None ---c.489+1G>T<br>12 = 2789+5G->A--- None --- c.2657+5G>A<br>13 = 3849+10kbC->T--- None--- c.3717+12191C>T<br>14 = R1162X--- p.Arg1162X --- c.3484C>T<br>15 = 2183AA->G or 2183delAA->G--- p.Lys684Serfs<br>16 = CFTRdele2 3 ---p.Ser18ArgfsX16 ---c.54-5940_<br>17 = G85E--- p.Gly85Glu ---- c.254G>A<br>18 = 3120+1G->A--- None--- c.2988+1G>A<br>19 = I507del ---p.Ile507del --- c.1519_1521delATC<br>20 = 1898+1G->A ---None--- c.1766+1G>A<br>21 = 3659delC--- p.Lys1177SerfsX15 --- c.3528delC<br>22 = R347P ---p.Arg347Pro --- c.1040G>C<br>23 = D1152H ---p.Asp1152His --- c.3454G>C<br>24 = R560T ---p.Arg560Thr --- c.1679G>C<br>25 = 3272-26A->G ---None ---c.3140-26A>G<br>26 = Q493X ---p.Gln493X --- c.1477C>T<br>27 = E60X--- p.Glu60X --- c.178G>T<br>28 = R334W ---p.Arg334Trp ---c.1000C>T<br>29 = 394delTT--- p.Leu88IlefsX22 --- c.262_263delTT<br>30 = 2184insA--- p.Gln685ThrfsX4 --- c.2052_2053in<br>31 = 5T;TG11--- None--- c.[1210-12[5];1210-34TG[1<br>32 = 5T;TG12--- None ---c.[1210-12[5];1210-34TG[1<br>33 = 5T;TG13 ---None--- c.[1210-12[5];1210-34TG[1<br>34 = 5T;TG not specified--- None--- c.1210-12[5]<br>35 = 3905insT--- p.Leu1258PhefsX7 --- c.3773_3774<br>36 = Y1092X--- p.Tyr1092X --- c.3276C>A<br>37 = Y1092X ---p.Tyr1092X --- c.3276C>G<br>38 = Y1092X--- p.Tyr1092X --- cDNA variant not spe<br>39 = A455E--- p.Ala455Glu --- c.1364C>A<br>40 = 2184delA--- p.Lys684AsnfsX38 --- c.2052delA<br>41 = R1066C--- p.Arg1066Cys --- c.3196C>T<br>42 = 1078delT--- p.Phe316LeufsX12 --- c.948delT<br>43 = 1154insTC--- p.Phe342HisfsX28 --- c.1022_102<br>44 = R1158X--- p.Arg1158X ---c.3472C>T<br>45 = R347H --- p.Arg347His --- c.1040G>A<br>46 = S1251N --- p.Ser1251Asn --- c.3752G>A<br>47 = L206W--- p.Leu206Trp --- c.617T>G<br>48 = S549N--- p.Ser549Asn --- c.1646G>A<br>49 = M1101K--- p.Met1101Lys --- c.3302T>A<br>50 = 711+1G->T--- None---- c.579+1G>T<br>51 = Y122X--- p.Tyr122X --- c.366T>A<br>52 = 2143delT--- p.Leu671X--- c.2012delT<br>53 = S945L--- p.Ser945Leu --- c.2834C>T<br>54 = I148T--- p.Ile148Thr --- c.443T>C<br>55 = R117C--- p.Arg117Cys --- c.349C>T<br>56 = V520F ---p.Val520Phe --- c.1558G>T<br>57 = S1235R --- p.Ser1235Arg --- c.3705T>G<br>58 = T338I--- p.Thr338Ile --- c.1013C>T<br>59 = P67L--- p.Pro67Leu --- c.200C>T<br>60 = G1244E ---p.Gly1244Glu ---c.3731G>A<br>61 = G178R--- p.Gly178Arg --- c.532G>A<br>62 = 1677delTA --- p.Tyr515X --- c.1545_1546delTA<br>63 = R352Q --- p.Arg352Gln --- c.1055G>A<br>64 = 711+5G->A --- None --- c.579+5G>A<br>65 = R668C --- p.Arg668Cys --- c.2002C>T<br>66 = S549R --- p.Ser549Arg --- c.1645A>C<br>67 = S549R ---p.Ser549Arg --- c.1647T>G<br>68 = S549R ---p.Ser549Arg ---cDNA variant not spe<br>69 = A559T--- p.Ala559Thr --- c.1675G>A<br>70 = L1077P--- p.Leu1077Pro ---c.3230T>C<br>71 = W1089X--- p.Trp1089X --- c.3266G>A<br>72 = I1027T--- p.Ile1027Thr --- c.3080T>C<br>73 = G576A ---p.Gly576Ala --- c.1727G>C<br>74 = M470V ---p.Met470Val --- c.1408A>G<br>75 = 3120G->A--- None --- c.2988G>A<br>76 = R75X --- p.Arg75X --- c.223C>T<br>77 = W846X --- p.Trp846X --- c.2537G>A<br>78 = E585X --- p.Glu585X --- c.1753G>T<br>79 = 1811+1.6kbA->G--- None --- c.1679+1.6kbA>G<br>80 = 3876delA--- p.Lys1250ArgfsX9 --- c.3744delA<br>81 = D1270N --- p.Asp1270Asn ---c.3808G>A<br>82 = Q220X ---p.Gln220X ---c.658C>T<br>83 = 2307insA--- p.Glu726ArgfsX4 --- c.2175_2176in<br>84 = D110H ---p.Asp110His --- c.328G>C<br>85 = 4016insT--- p.Ser1297PhefsX5 --- c.3884_3885i<br>86 = 4382delA--- p.Glu1418ArgfsX14 --- c.4251delA<br>87 = I336K ---p.Ile336Lys ---c.1007T>A<br>88 = R1066H--- p.Arg1066His --- c.3197G>A<br>89 = 2347delG--- p.Val739TyrfsX16 --- c.2215delG<br>90 = L997F ---p.Leu997Phe --- c.2991G>C<br>91 = K710X--- p.Lys710X --- c.2128A>T<br>92 = E822X ---p.Glu822X --- c.2464G>T<br>93 = L1065P ---p.Leu1065Pro --- c.3194T>C<br>94 = Q552X ---p.Gln552X ---c.1654C>T<br>95 = R74W--- p.Arg74Trp --- c.220C>T<br>96 = 2622+1G->A--- None --- c.2490+1G>A<br>97 = 2789+2insA--- None--- c.2657+2_2657+3insA<br>98 = E92X--- p.Glu92X --- c.274G>T<br>99 = Q39X--- p.Gln39X --- c.115C>T<br>100 = R75Q--- p.Arg75Gln --- c.224G>A<br>101 = D579G--- p.Asp579Gly --- c.1736A>G<br>102 = E831X--- p.Glu831X --- c.2491G>T<br>103 = 3007delG --- p.Ala959HisfsX9 --- c.2875delG<br>104 = 405+1G->A --- None ---c.273+1G>A<br>105 = 406-1G->A--- None ---c.274-1G>A<br>106 = 711+3A->G--- None--- c.579+3A>G<br>107 = Q1313X--- p.Gln1313X --- c.3937C>T<br>108 = R709X--- p.Arg709X --- c.2125C>T<br>109 = 2711delT--- p.Phe861LeufsX3 --- c.2583delT<br>110 = 4005+1G->A--- None --- c.3873+1G>A<br>111 = 574delA--- p.Ile148LeufsX5 --- c.442delA<br>112 = 1525-1G->A--- None--- c.1393-1G>A<br>113 = 1812-1G->A--- None--- c.1680-1G>A<br>114 = I1234V ---p.Ile1234Val --- c.3700A>G<br>115 = R1070Q--- p.Arg1070Gln--- c.3209G>A<br>116 = S466X ---p.Ser466X --- c.1397C>A<br>117 = S466X--- p.Ser466X --- c.1397C>G<br>118 = S466X--- p.Ser466X --- cDNA variant not spec<br>119 = S489X--- p.Ser489X --- c.1466C>A<br>120 = L467P--- p.Leu467Pro --- c.1400T>C<br>121 = S492F--- p.Ser492Phe --- c.1475C>T<br>122 = 3791delC--- p.Thr1220LysfsX8 --- c.3659delC<br>123 = L927P--- p.Leu927Pro --- c.2780T>C<br>124 = 712-1G->T--- None--- c.580-1G>T<br>125 = E92K--- p.Glu92Lys --- c.274G>A<br>126 = Q359K/T360K--- p.[Gln359Lys;Thr360Lys] ---<br>127 = Q890X ---p.Gln890X --- c.2668C>T<br>128 = R764X ---p.Arg764X --- c.2290C>T<br>129 = S1196X ---p.Ser1196X --- c.3587C>G<br>130 = W401X--- p.Trp401X --- c.1202G>A<br>131 = W401X ---p.Trp401X --- c.1203G>A<br>132 = W401X ---p.Trp401X --- cDNA variant not spec<br>133 = 1248+1G->A--- None--- c.1116+1G>A<br>134 = L732X--- p.Leu732X ---c.2195T>G<br>135 = Q98X--- p.Gln98X --- c.292C>T<br>136 = R1070W--- p.Arg1070Trp--- c.3208C>T<br>137 = R31C--- p.Arg31Cys --- c.91C>T<br>138 = R851X ---p.Arg851X ---c.2551C>T<br>139 = W1204X--- p.Trp1204X --- c.3611G>A<br>140 = W1204X--- p.Trp1204X --- c.3612G>A<br>141 = W1204X--- p.Trp1204X --- cDNA variant not sp<br>142 = 663delT--- p.Ile177MetfsX12 --- c.531delT<br>143 = F1052V--- p.Phe1052Val--- c.3154T>G<br>144 = G330X ---p.Gly330X --- c.988G>T<br>145 = P205S--- p.Pro205Ser --- c.613C>T<br>146 = 1259insA--- p.Gln378AlafsX4 --- c.1127_1128i<br>147 = 2585delT--- p.Leu818TrpfsX3 --- c.2453delT<br>148 = 852del22--- p.Gly241GlufsX13 --- c.720_741d<br>149 = E1104X ---p.Glu1104X --- c.3310G>T<br>150 = H199Y ---p.His199Tyr --- c.595C>T<br>151 = Q525X ---p.Gln525X --- c.1573C>T<br>152 = 1461ins4--- p.Ile444ArgfsX3 --- c.1329_1330in<br>153 = 1898+3A->G--- None--- c.1766+3A>G<br>154 = 7T--- None--- c.1210-12[7]<br>155 = CFTRdele22 23 ---None ---c.3964-78_4242+5<br>156 = D614G--- p.Asp614Gly --- c.1841A>G<br>157 = L227R--- p.Leu227Arg ---c.680T>G<br>158 = L558S--- p.Leu558Ser --- c.1673T>C<br>159 = 1213delT--- p.Trp361GlyfsX8 --- c.1081delT<br>160 = 1341+1G->A--- None--- c.1209+1G>A<br>161 = 1548delG--- p.Gly473GlufsX54 --- c.1418delG<br>162 = 1717-8G->A--- None--- c.1585-8G>A<br>163 = 3121-1G->A--- None--- c.2989-1G>A<br>164 = 4209TGTT->AA--- None--- c.4077_4080delTG<br>165 = 457TAT->G--- p.Tyr109GlyfsX4 --- c.325_327d<br>166 = G1069R ---p.Gly1069Arg --- c.3205G>A<br>167 = G970R--- p.Gly970Arg--- c.2908G>C<br>168 = M1V--- p.Met1Val --- c.1A>G<br>169 = R1162L--- p.Arg1162Leu --- c.3485G>T<br>170 = R560K--- p.Arg560Lys --- c.1679G>A<br>171 = S341P--- p.Ser341Pro --- c.1021T>C<br>172 = S977F--- p.Ser977Phe --- c.2930C>T<br>173 = V754M ---p.Val754Met--- c.2260G>A<br>174 = Y569D ---p.Tyr569Asp ---c.1705T>G<br>175 = 2594delGT ---p.Ser821ArgfsX4 --- c.2462_246<br>176 = 4374+1G->T--- None--- c.4242+1G>T<br>177 = R785X--- p.Arg785X --- c.2353C>T<br>178 = 2055del9->A--- p.Ser641ArgfsX5--- c.1923_1<br>179 = 2105-2117del13insAGAAA--- p.Arg658LysfsX4<br>180 = G551S--- p.Gly551Ser --- c.1651G>A<br>181 = A561E--- p.Ala561Glu --- c.1682C>A<br>182 = H1054D--- p.His1054Asp --- c.3160C>G<br>183 = 1288insTA--- p.Asn386IlefsX3 --- c.1153_1154<br>184 = 1471delA--- p.Lys447ArgfsX2 --- c.1340delA<br>185 = 444delA--- p.Ile105SerfsX2--- c.313delA<br>186 = 3667ins4--- p.Thr1179IlefsX17 --- c.3535_353<br>187 = 3821delT--- p.Ser1231ProfsX4 --- c.3691delT<br>188 = 4326delTC--- p.Cys1400X --- c.4196_4197del<br>189 = R792X--- p.Arg792X --- c.2374C>T<br>190 = S912X--- p.Ser912X --- c.2735C>A<br>191 = C276X ---p.Cys276X --- c.828C>A<br>192 = 1811+1G->C--- None--- c.1679+1G>C<br>193 = 3850-1G->A ---None ---c.3718-1G>A<br>194 = 306insA ---p.Arg59LysfsX10 --- c.174_175insA<br>195 = A46D ---p.Ala46Asp --- c.137C>A<br>196 = 1782delA--- p.Gly551ValfsX8 --- c.1650delA<br>197 = 2118del4--- p.Thr663ArgfsX8 --- c.1986_1989<br>198 = 2869insG--- p.Tyr913X --- c.2737_2738insG<br>199 = 2896insAG--- p.Val922GlufsX2 --- c.2764_276<br>200 = 3132delTG--- p.Val1001AspfsX45 --- c.3002_3<br>201 = 3737delA--- p.Asp1202AlafsX9 --- c.3605delA<br>202 = Q414X--- p.Gln414X --- c.1240C>T<br>203 = S1255X--- p.Ser1255X --- c.3764C>A<br>204 = E56K--- p.Glu56Lys ---c.166G>A<br>205 = G1061R--- p.Gly1061Arg--- c.3181G>C<br>206 = F1074L ---p.Phe1074Leu --- c.3222T>A<br>207 = G1349D--- p.Gly1349Asp --- c.4046G>A<br>208 = 4428insGA--- p.Ser1435GlyfsX14 --- c.4296_4<br>209 = Y849X--- p.Tyr849X --- c.2547C>A<br>210 = E1371X ---p.Glu1371X--- c.4111G>T<br>211 = 1898+1G->C--- None ---c.1766+1G>C<br>212 = 2790-1G->C--- None--- c.2658-1G>C<br>213 = 297-1G->A--- None--- c.165-1G>A<br>214 = 1824delA--- p.Asp565MetfsX7 --- c.1692delA<br>215 = 2556insAT--- p.Ser809IlefsX13 --- c.2424_2425<br>216 = 3121-977_3499+248del2515--- None--- c.298<br>217 = 4015delA--- p.Ile1295PhefsX33--- c.3883delA<br>218 = 675del4--- p.Leu183PhefsX5 --- c.543_546del<br>219 = Y913X--- p.Tyr913X --- c.2739T>A<br>220 = Q1412X--- p.Gln1412X --- c.4234C>T<br>221 = S1255P--- p.Ser1255Pro --- c.3763T>C<br>222 = CFTRdele17a-18--- None--- c.(2988+1_2989-1<br>223 = CFTRdele2--- None--- c.(53+1_54-1)_(164+1_<br>224 = CFTRdele22-24 ---None--- c.(3963+1_3964-1)<br>225 = L1254X ---p.Leu1254X--- c.3761T>G<br>226 = 1138insG--- p.Ile336SerfsX28--- c.1006_1007i<br>227 = 935delA--- p.Asn268IlefsX17--- c.803delA<br>228 = 1161delC--- p.Cys343X--- c.1029delC<br>229 = 1609delCA ---p.Gln493ValfsX10--- c.1477_147<br>230 = CFTRdele14b-17b--- None ---c.(2619+1_2620<br>231 = CFTRdele17a 17b--- None--- c.(2988+1_2989-<br>232 = E193X ---p.Glu193X--- c.577G>T<br>[illegible]

1	Baseline	1.2.1	genospec2	Specify other Genotype		string				

1	Baseline	1.3	genotype3	Genotype (Mutation 3)		option			1 = F508del--- p.Phe508del--- c.1521_1523delCTT<br>2 = G542X ---p.Gly542X ---- c.1624G>T<br>3 = G551D--- p.Gly551Asp --- c.1652G>A<br>4 = N1303K--- p.Asn1303Lys --- c.3909C>G<br>5 = W1282X--- p.Trp1282X --- c.3846G>A---<br>6 = R117H;5T--- p.Arg117His;None(5T) --- c.[350G>A<br>7 = R117H;7T--- p.Arg117His;None(7T) --- c.[350G>A<br>8 = R117H--- p.Arg117His --- c.350G>A<br>9 = R553X ---p.Arg553X --- c.1657C>T<br>10 = 1717-1G->A--- None --- c.1585-1G>A<br>11 = 621+1G->T--- None ---c.489+1G>T<br>12 = 2789+5G->A--- None --- c.2657+5G>A<br>13 = 3849+10kbC->T--- None--- c.3717+12191C>T<br>14 = R1162X--- p.Arg1162X --- c.3484C>T<br>15 = 2183AA->G or 2183delAA->G--- p.Lys684Serfs<br>16 = CFTRdele2 3 ---p.Ser18ArgfsX16 ---c.54-5940_<br>17 = G85E--- p.Gly85Glu ---- c.254G>A<br>18 = 3120+1G->A--- None--- c.2988+1G>A<br>19 = I507del ---p.Ile507del --- c.1519_1521delATC<br>20 = 1898+1G->A ---None--- c.1766+1G>A<br>21 = 3659delC--- p.Lys1177SerfsX15 --- c.3528delC<br>22 = R347P ---p.Arg347Pro --- c.1040G>C<br>23 = D1152H ---p.Asp1152His --- c.3454G>C<br>24 = R560T ---p.Arg560Thr --- c.1679G>C<br>25 = 3272-26A->G ---None ---c.3140-26A>G<br>26 = Q493X ---p.Gln493X --- c.1477C>T<br>27 = E60X--- p.Glu60X --- c.178G>T<br>28 = R334W ---p.Arg334Trp ---c.1000C>T<br>29 = 394delTT--- p.Leu88IlefsX22 --- c.262_263delTT<br>30 = 2184insA--- p.Gln685ThrfsX4 --- c.2052_2053in<br>31 = 5T;TG11--- None--- c.[1210-12[5];1210-34TG[1<br>32 = 5T;TG12--- None ---c.[1210-12[5];1210-34TG[1<br>33 = 5T;TG13 ---None--- c.[1210-12[5];1210-34TG[1<br>34 = 5T;TG not specified--- None--- c.1210-12[5]<br>35 = 3905insT--- p.Leu1258PhefsX7 --- c.3773_3774<br>36 = Y1092X--- p.Tyr1092X --- c.3276C>A<br>37 = Y1092X ---p.Tyr1092X --- c.3276C>G<br>38 = Y1092X--- p.Tyr1092X --- cDNA variant not spe<br>39 = A455E--- p.Ala455Glu --- c.1364C>A<br>40 = 2184delA--- p.Lys684AsnfsX38 --- c.2052delA<br>41 = R1066C--- p.Arg1066Cys --- c.3196C>T<br>42 = 1078delT--- p.Phe316LeufsX12 --- c.948delT<br>43 = 1154insTC--- p.Phe342HisfsX28 --- c.1022_102<br>44 = R1158X--- p.Arg1158X ---c.3472C>T<br>45 = R347H --- p.Arg347His --- c.1040G>A<br>46 = S1251N --- p.Ser1251Asn --- c.3752G>A<br>47 = L206W--- p.Leu206Trp --- c.617T>G<br>48 = S549N--- p.Ser549Asn --- c.1646G>A<br>49 = M1101K--- p.Met1101Lys --- c.3302T>A<br>50 = 711+1G->T--- None---- c.579+1G>T<br>51 = Y122X--- p.Tyr122X --- c.366T>A<br>52 = 2143delT--- p.Leu671X--- c.2012delT<br>53 = S945L--- p.Ser945Leu --- c.2834C>T<br>54 = I148T--- p.Ile148Thr --- c.443T>C<br>55 = R117C--- p.Arg117Cys --- c.349C>T<br>56 = V520F ---p.Val520Phe --- c.1558G>T<br>57 = S1235R --- p.Ser1235Arg --- c.3705T>G<br>58 = T338I--- p.Thr338Ile --- c.1013C>T<br>59 = P67L--- p.Pro67Leu --- c.200C>T<br>60 = G1244E ---p.Gly1244Glu ---c.3731G>A<br>61 = G178R--- p.Gly178Arg --- c.532G>A<br>62 = 1677delTA --- p.Tyr515X --- c.1545_1546delTA<br>63 = R352Q --- p.Arg352Gln --- c.1055G>A<br>64 = 711+5G->A --- None --- c.579+5G>A<br>65 = R668C --- p.Arg668Cys --- c.2002C>T<br>66 = S549R --- p.Ser549Arg --- c.1645A>C<br>67 = S549R ---p.Ser549Arg --- c.1647T>G<br>68 = S549R ---p.Ser549Arg ---cDNA variant not spe<br>69 = A559T--- p.Ala559Thr --- c.1675G>A<br>70 = L1077P--- p.Leu1077Pro ---c.3230T>C<br>71 = W1089X--- p.Trp1089X --- c.3266G>A<br>72 = I1027T--- p.Ile1027Thr --- c.3080T>C<br>73 = G576A ---p.Gly576Ala --- c.1727G>C<br>74 = M470V ---p.Met470Val --- c.1408A>G<br>75 = 3120G->A--- None --- c.2988G>A<br>76 = R75X --- p.Arg75X --- c.223C>T<br>77 = W846X --- p.Trp846X --- c.2537G>A<br>78 = E585X --- p.Glu585X --- c.1753G>T<br>79 = 1811+1.6kbA->G--- None --- c.1679+1.6kbA>G<br>80 = 3876delA--- p.Lys1250ArgfsX9 --- c.3744delA<br>81 = D1270N --- p.Asp1270Asn ---c.3808G>A<br>82 = Q220X ---p.Gln220X ---c.658C>T<br>83 = 2307insA--- p.Glu726ArgfsX4 --- c.2175_2176in<br>84 = D110H ---p.Asp110His --- c.328G>C<br>85 = 4016insT--- p.Ser1297PhefsX5 --- c.3884_3885i<br>86 = 4382delA--- p.Glu1418ArgfsX14 --- c.4251delA<br>87 = I336K ---p.Ile336Lys ---c.1007T>A<br>88 = R1066H--- p.Arg1066His --- c.3197G>A<br>89 = 2347delG--- p.Val739TyrfsX16 --- c.2215delG<br>90 = L997F ---p.Leu997Phe --- c.2991G>C<br>91 = K710X--- p.Lys710X --- c.2128A>T<br>92 = E822X ---p.Glu822X --- c.2464G>T<br>93 = L1065P ---p.Leu1065Pro --- c.3194T>C<br>94 = Q552X ---p.Gln552X ---c.1654C>T<br>95 = R74W--- p.Arg74Trp --- c.220C>T<br>96 = 2622+1G->A--- None --- c.2490+1G>A<br>97 = 2789+2insA--- None--- c.2657+2_2657+3insA<br>98 = E92X--- p.Glu92X --- c.274G>T<br>99 = Q39X--- p.Gln39X --- c.115C>T<br>100 = R75Q--- p.Arg75Gln --- c.224G>A<br>101 = D579G--- p.Asp579Gly --- c.1736A>G<br>102 = E831X--- p.Glu831X --- c.2491G>T<br>103 = 3007delG --- p.Ala959HisfsX9 --- c.2875delG<br>104 = 405+1G->A --- None ---c.273+1G>A<br>105 = 406-1G->A--- None ---c.274-1G>A<br>106 = 711+3A->G--- None--- c.579+3A>G<br>107 = Q1313X--- p.Gln1313X --- c.3937C>T<br>108 = R709X--- p.Arg709X --- c.2125C>T<br>109 = 2711delT--- p.Phe861LeufsX3 --- c.2583delT<br>110 = 4005+1G->A--- None --- c.3873+1G>A<br>111 = 574delA--- p.Ile148LeufsX5 --- c.442delA<br>112 = 1525-1G->A--- None--- c.1393-1G>A<br>113 = 1812-1G->A--- None--- c.1680-1G>A<br>114 = I1234V ---p.Ile1234Val --- c.3700A>G<br>115 = R1070Q--- p.Arg1070Gln--- c.3209G>A<br>116 = S466X ---p.Ser466X --- c.1397C>A<br>117 = S466X--- p.Ser466X --- c.1397C>G<br>118 = S466X--- p.Ser466X --- cDNA variant not spec<br>119 = S489X--- p.Ser489X --- c.1466C>A<br>120 = L467P--- p.Leu467Pro --- c.1400T>C<br>121 = S492F--- p.Ser492Phe --- c.1475C>T<br>122 = 3791delC--- p.Thr1220LysfsX8 --- c.3659delC<br>123 = L927P--- p.Leu927Pro --- c.2780T>C<br>124 = 712-1G->T--- None--- c.580-1G>T<br>125 = E92K--- p.Glu92Lys --- c.274G>A<br>126 = Q359K/T360K--- p.[Gln359Lys;Thr360Lys] ---<br>127 = Q890X ---p.Gln890X --- c.2668C>T<br>128 = R764X ---p.Arg764X --- c.2290C>T<br>129 = S1196X ---p.Ser1196X --- c.3587C>G<br>130 = W401X--- p.Trp401X --- c.1202G>A<br>131 = W401X ---p.Trp401X --- c.1203G>A<br>132 = W401X ---p.Trp401X --- cDNA variant not spec<br>133 = 1248+1G->A--- None--- c.1116+1G>A<br>134 = L732X--- p.Leu732X ---c.2195T>G<br>135 = Q98X--- p.Gln98X --- c.292C>T<br>136 = R1070W--- p.Arg1070Trp--- c.3208C>T<br>137 = R31C--- p.Arg31Cys --- c.91C>T<br>138 = R851X ---p.Arg851X ---c.2551C>T<br>139 = W1204X--- p.Trp1204X --- c.3611G>A<br>140 = W1204X--- p.Trp1204X --- c.3612G>A<br>141 = W1204X--- p.Trp1204X --- cDNA variant not sp<br>142 = 663delT--- p.Ile177MetfsX12 --- c.531delT<br>143 = F1052V--- p.Phe1052Val--- c.3154T>G<br>144 = G330X ---p.Gly330X --- c.988G>T<br>145 = P205S--- p.Pro205Ser --- c.613C>T<br>146 = 1259insA--- p.Gln378AlafsX4 --- c.1127_1128i<br>147 = 2585delT--- p.Leu818TrpfsX3 --- c.2453delT<br>148 = 852del22--- p.Gly241GlufsX13 --- c.720_741d<br>149 = E1104X ---p.Glu1104X --- c.3310G>T<br>150 = H199Y ---p.His199Tyr --- c.595C>T<br>151 = Q525X ---p.Gln525X --- c.1573C>T<br>152 = 1461ins4--- p.Ile444ArgfsX3 --- c.1329_1330in<br>153 = 1898+3A->G--- None--- c.1766+3A>G<br>154 = 7T--- None--- c.1210-12[7]<br>155 = CFTRdele22 23 ---None ---c.3964-78_4242+5<br>156 = D614G--- p.Asp614Gly --- c.1841A>G<br>157 = L227R--- p.Leu227Arg ---c.680T>G<br>158 = L558S--- p.Leu558Ser --- c.1673T>C<br>159 = 1213delT--- p.Trp361GlyfsX8 --- c.1081delT<br>160 = 1341+1G->A--- None--- c.1209+1G>A<br>161 = 1548delG--- p.Gly473GlufsX54 --- c.1418delG<br>162 = 1717-8G->A--- None--- c.1585-8G>A<br>163 = 3121-1G->A--- None--- c.2989-1G>A<br>164 = 4209TGTT->AA--- None--- c.4077_4080delTG<br>165 = 457TAT->G--- p.Tyr109GlyfsX4 --- c.325_327d<br>166 = G1069R ---p.Gly1069Arg --- c.3205G>A<br>167 = G970R--- p.Gly970Arg--- c.2908G>C<br>168 = M1V--- p.Met1Val --- c.1A>G<br>169 = R1162L--- p.Arg1162Leu --- c.3485G>T<br>170 = R560K--- p.Arg560Lys --- c.1679G>A<br>171 = S341P--- p.Ser341Pro --- c.1021T>C<br>172 = S977F--- p.Ser977Phe --- c.2930C>T<br>173 = V754M ---p.Val754Met--- c.2260G>A<br>174 = Y569D ---p.Tyr569Asp ---c.1705T>G<br>175 = 2594delGT ---p.Ser821ArgfsX4 --- c.2462_246<br>176 = 4374+1G->T--- None--- c.4242+1G>T<br>177 = R785X--- p.Arg785X --- c.2353C>T<br>178 = 2055del9->A--- p.Ser641ArgfsX5--- c.1923_1<br>179 = 2105-2117del13insAGAAA--- p.Arg658LysfsX4<br>180 = G551S--- p.Gly551Ser --- c.1651G>A<br>181 = A561E--- p.Ala561Glu --- c.1682C>A<br>182 = H1054D--- p.His1054Asp --- c.3160C>G<br>183 = 1288insTA--- p.Asn386IlefsX3 --- c.1153_1154<br>184 = 1471delA--- p.Lys447ArgfsX2 --- c.1340delA<br>185 = 444delA--- p.Ile105SerfsX2--- c.313delA<br>186 = 3667ins4--- p.Thr1179IlefsX17 --- c.3535_353<br>187 = 3821delT--- p.Ser1231ProfsX4 --- c.3691delT<br>188 = 4326delTC--- p.Cys1400X --- c.4196_4197del<br>189 = R792X--- p.Arg792X --- c.2374C>T<br>190 = S912X--- p.Ser912X --- c.2735C>A<br>191 = C276X ---p.Cys276X --- c.828C>A<br>192 = 1811+1G->C--- None--- c.1679+1G>C<br>193 = 3850-1G->A ---None ---c.3718-1G>A<br>194 = 306insA ---p.Arg59LysfsX10 --- c.174_175insA<br>195 = A46D ---p.Ala46Asp --- c.137C>A<br>196 = 1782delA--- p.Gly551ValfsX8 --- c.1650delA<br>197 = 2118del4--- p.Thr663ArgfsX8 --- c.1986_1989<br>198 = 2869insG--- p.Tyr913X --- c.2737_2738insG<br>199 = 2896insAG--- p.Val922GlufsX2 --- c.2764_276<br>200 = 3132delTG--- p.Val1001AspfsX45 --- c.3002_3<br>201 = 3737delA--- p.Asp1202AlafsX9 --- c.3605delA<br>202 = Q414X--- p.Gln414X --- c.1240C>T<br>203 = S1255X--- p.Ser1255X --- c.3764C>A<br>204 = E56K--- p.Glu56Lys ---c.166G>A<br>205 = G1061R--- p.Gly1061Arg--- c.3181G>C<br>206 = F1074L ---p.Phe1074Leu --- c.3222T>A<br>207 = G1349D--- p.Gly1349Asp --- c.4046G>A<br>208 = 4428insGA--- p.Ser1435GlyfsX14 --- c.4296_4<br>209 = Y849X--- p.Tyr849X --- c.2547C>A<br>210 = E1371X ---p.Glu1371X--- c.4111G>T<br>211 = 1898+1G->C--- None ---c.1766+1G>C<br>212 = 2790-1G->C--- None--- c.2658-1G>C<br>213 = 297-1G->A--- None--- c.165-1G>A<br>214 = 1824delA--- p.Asp565MetfsX7 --- c.1692delA<br>215 = 2556insAT--- p.Ser809IlefsX13 --- c.2424_2425<br>216 = 3121-977_3499+248del2515--- None--- c.298<br>217 = 4015delA--- p.Ile1295PhefsX33--- c.3883delA<br>218 = 675del4--- p.Leu183PhefsX5 --- c.543_546del<br>219 = Y913X--- p.Tyr913X --- c.2739T>A<br>220 = Q1412X--- p.Gln1412X --- c.4234C>T<br>221 = S1255P--- p.Ser1255Pro --- c.3763T>C<br>222 = CFTRdele17a-18--- None--- c.(2988+1_2989-1<br>223 = CFTRdele2--- None--- c.(53+1_54-1)_(164+1_<br>224 = CFTRdele22-24 ---None--- c.(3963+1_3964-1)<br>225 = L1254X ---p.Leu1254X--- c.3761T>G<br>226 = 1138insG--- p.Ile336SerfsX28--- c.1006_1007i<br>227 = 935delA--- p.Asn268IlefsX17--- c.803delA<br>228 = 1161delC--- p.Cys343X--- c.1029delC<br>229 = 1609delCA ---p.Gln493ValfsX10--- c.1477_147<br>230 = CFTRdele14b-17b--- None ---c.(2619+1_2620<br>231 = CFTRdele17a 17b--- None--- c.(2988+1_2989-<br>232 = E193X ---p.Glu193X--- c.577G>T<br>[illegible]

**1**	Baseline	1.3.1	genospec3	Specify other Genotype		string			
**1**	Baseline	1.4	sweat_chloride_level	Sweat chloride level		integer	mmol/L		
**1**	Baseline	1.4	sweat_chloride_level_na	N/A		checkbox		0	1 = Yes<br>0 = No
**1**	Baseline	2.1	paninsuff	Does this patient have pancreatic insufficiency (e.g. faecal elastase < 200)?		boolean			1 = Yes<br>0 = No
**1**	Baseline	2.2	cfdiab	Does this patient have CF related diabetes (prescribed regular insulin)?		boolean			1 = Yes<br>0 = No
**1**	Baseline	2.3	height	Height	Height at presentation	decimal	cm		
**1**	Baseline	2.4	weight	Weight	Weight at presentation	decimal	kg		
**1**	Baseline	3.1	abxreact	Are there any antibiotics contraindicated for this participant because of previous reactions or a high susceptibility to toxicity e.g. renal impairment?		boolean			1 = Yes<br>0 = No
**1**	Baseline	3.1.1	abxreacttrt	Has the participant had a reaction relevant to the future safe use of any of the following antibiotics?		boolean			1 = Yes<br>0 = No
**2**	Clinic Visit	1.1	cvdat	Date of clinic visit		date			
**2**	Clinic Visit	1.2	cvpladmyn	Have any admissions for the near future been planned at this clinic visit?		boolean			1 = Yes<br>0 = No
**2**	Clinic Visit	1.2.1	cvpladmdat	Date of planned admission		date			
**2**	Clinic Visit	1.3	cvhgtupdyn	Previous height recorded is %{previous_height} cm, does this need to be updated?		boolean			1 = Yes<br>0 = No
**2**	Clinic Visit	1.3.1	cvhgt	Height		decimal	cm		
**2**	Clinic Visit	1.4	cvwgtupdyn	Previous weight recorded is %{previous_weight} kg, does this need to be updated?		boolean			1 = Yes<br>0 = No
**2**	Clinic Visit	1.4.1	cvwgt	Weight		decimal	kg		
**2**	Clinic Visit	1.5	cvpaninsuff	Does this patient have pancreatic insufficiency (faecal elastase < 200)?		boolean			1 = Yes<br>0 = No
**2**	Clinic Visit	1.6	cvcfdiab	Does this patient have CF related diabetes (prescribed regular insulin)?		boolean			1 = Yes<br>0 = No
**2**	Clinic Visit	2.1	cvfev1	Any FEV1 results since the last visit on %{previous_visit_date}, including this current visit?		boolean			1 = Yes<br>0 = No
**2**	Clinic Visit	3.1	cvmicrochg	Have any new airway samples for microbiology been taken and tested?		boolean			1 = Yes<br>0 = No
**2**	Clinic Visit	3.2	cvcmchg	Have there been any changes to Concomitant Medications?		boolean			1 = Yes<br>0 = No
**2**	Clinic Visit	3.3	cvcichg	Have there been any changes to Antibiotic Contraindications?		boolean			1 = Yes<br>0 = No
**3.1**	Airway Samples for Microbiology	1.1	sample_date	Date		date			
**3.1**	Airway Samples for Microbiology	1.2	sample_type	Type		option			1 = Expectorated sputum<br>2 = Oropharyngeal swab<br>3 = Bronchoalveolar lavage<br>4 = Induced sputum<br>5 = Laryngopharyngeal suction<br>6 = Nasopharyngeal swab
**3.1**	Airway Samples for Microbiology	1.3	sample_source	Source		string			
**3.1**	Airway Samples for Microbiology	1.4	growth_detected	Growth detected		boolean			1 = Yes<br>0 = No
**3.1**	Airway Samples for Microbiology	1.4.1	organism_count	No. of distinct organism types detected		integer			
**3.2**	Micro-organisms from Airway Samples	1.1	sample_id	Sample		linked_form			
**3.2**	Micro-organisms from Airway Samples	1.2	organism_type	Organism type		option			1 = Pseudomonas<br>2 = Staphylococcus<br>4 = Haemophilius<br>5 = Pneumococcus<br>6 = Stenotrophomonas<br>8 = Acinetobacter<br>9 = Burkholderia<br>10 = Mycobacterium<br>12 = Mixed oral flora/URTF<br>99 = Other bacteria<br>101 = Adenovirus<br>102 = Bocavirus<br>103 = Coronavirus (not SARS-CoV-2)<br>107 = COVID-19 (SARS-CoV-2)<br>104 = Enterovirus<br>105 = Influenza (type not specified)<br>106 = Influenza type A<br>108 = Influenza type B<br>109 = Metapneumovirus<br>110 = Parainfluenza (type not specified)<br>111 = Parainfluenza type 1<br>112 = Parainfluenza type 2<br>113 = Parainfluenza type 3<br>114 = Parainfluenza type 4<br>115 = Rhinovirus<br>116 = RSV (Respiratory syncytial virus) (type not spec<br>117 = RSV (Respiratory syncytial virus) type A<br>118 = RSV (Respiratory syncytial virus) type B<br>199 = Other virus<br>201 = mycoplasma<br>202 = legionella<br>203 = chlamydophila<br>7 = Aspergillus<br>11 = Candida<br>399 = Other fungi
**3.2**	Micro-organisms from Airway Samples	1.2a	organism_type_oth	Other		string			
**3.2**	Micro-organisms from Airway Samples	1.2b	organism_description	Description		string			
**3.2**	Micro-organisms from Airway Samples	1.2.1	organism_esbl_detected	ESBL detected for this organism?		boolean			1 = Yes<br>0 = No
**3.2**	Micro-organisms from Airway Samples	1.2.2	mucoid_status	Mucoid status		option			1 = Mucoid<br>2 = Not mucoid<br>3 = Not reported
**3.2**	Micro-organisms from Airway Samples	1.2.3	mrsa_status	MRSA		option			-1 = Not reported<br>0 = No<br>1 = Yes
**3.2**	Micro-organisms from Airway Samples	1.3	antibiotic_susceptibility_tested	Has any antibiotic susceptibility testing been done for this organism?		boolean			1 = Yes<br>0 = No
**3.2**	Micro-organisms from Airway Samples	1.3.1.1	antibiotic_type_1	Antibiotic type 1		option			1 = Amoxicillin<br>2 = Amoxicillin clavulanate<br>4 = Amikacin<br>5 = Amphotericin B<br>6 = Ampicillin<br>7 = Azithromycin<br>8 = Aztreonam<br>12 = Cefepime<br>13 = Cefotaxime<br>14 = Ceftriaxone<br>15 = Ceftazidime<br>59 = Ceftazidime/avibactam<br>16 = Cephalexin<br>19 = Ciprofloxacin<br>21 = Clindamycin<br>22 = Colistin<br>23 = Cotrimoxazole (Trimethoprim and sulfamethoxaz<br>28 = Erythromycin<br>29 = Flucloxacillin<br>32 = Fusidic acid<br>33 = Gentamicin<br>34 = Imipenem<br>35 = Itraconazole<br>38 = Meropenem<br>58 = Methicillin<br>41 = Moxifloxacin<br>45 = Ofloxacin<br>46 = Penicillin/benzylpenicillin<br>47 = Piperacillin tazobactam (Tazocin)<br>48 = Posaconazole<br>49 = Rifampicin<br>52 = Ticarcillin clavulanate (Timentin)<br>53 = Tigecycline<br>54 = Tobramycin<br>56 = Vancomycin<br>57 = Voriconazole<br>999 = Other
**3.2**	Micro-organisms from Airway Samples	1.3.1.1a	antibiotic_type_other_1	Antibiotic type other 1		string			
**3.2**	Micro-organisms from Airway Samples	1.3.1.2	antibiotic_susceptibility_1	Antibiotic susceptibility 1		option			2 = Susceptible<br>3 = Intermediate<br>4 = Resistant<br>5 = Susceptible/Intermediate<br>6 = Intermediate/Resistant
**3.2**	Micro-organisms from Airway Samples	1.3.2.1	antibiotic_type_2	Antibiotic type 2		option			1 = Amoxicillin<br>2 = Amoxicillin clavulanate<br>4 = Amikacin<br>5 = Amphotericin B<br>6 = Ampicillin<br>7 = Azithromycin<br>8 = Aztreonam<br>12 = Cefepime<br>13 = Cefotaxime<br>14 = Ceftriaxone<br>15 = Ceftazidime<br>59 = Ceftazidime/avibactam<br>16 = Cephalexin<br>19 = Ciprofloxacin<br>21 = Clindamycin<br>22 = Colistin<br>23 = Cotrimoxazole (Trimethoprim and sulfamethoxaz<br>28 = Erythromycin<br>29 = Flucloxacillin<br>32 = Fusidic acid<br>33 = Gentamicin<br>34 = Imipenem<br>35 = Itraconazole<br>38 = Meropenem<br>58 = Methicillin<br>41 = Moxifloxacin<br>45 = Ofloxacin<br>46 = Penicillin/benzylpenicillin<br>47 = Piperacillin tazobactam (Tazocin)<br>48 = Posaconazole<br>49 = Rifampicin<br>52 = Ticarcillin clavulanate (Timentin)<br>53 = Tigecycline<br>54 = Tobramycin<br>56 = Vancomycin<br>57 = Voriconazole<br>999 = Other
**3.2**	Micro-organisms from Airway Samples	1.3.2.1a	antibiotic_type_other_2	Antibiotic type other 2		string			
**3.2**	Micro-organisms from Airway Samples	1.3.2.2	antibiotic_susceptibility_2	Antibiotic susceptibility 2		option			2 = Susceptible<br>3 = Intermediate<br>4 = Resistant<br>5 = Susceptible/Intermediate<br>6 = Intermediate/Resistant

**3.2**	Micro-organisms from Airway Samples	1.3.3.1	antibiotic_type_3	Antibiotic type 3		option			1 = Amoxicillin<br>2 = Amoxicillin clavulanate<br>4 = Amikacin<br>5 = Amphotericin B<br>6 = Ampicillin<br>7 = Azithromycin<br>8 = Aztreonam<br>12 = Cefepime<br>13 = Cefotaxime<br>14 = Ceftriaxone<br>15 = Ceftazidime<br>59 = Ceftazidime/avibactam<br>16 = Cephalexin<br>19 = Ciprofloxacin<br>21 = Clindamycin<br>22 = Colistin<br>23 = Cotrimoxazole (Trimethoprim and sulfamethoxaz<br>28 = Erythromycin<br>29 = Flucloxacillin<br>32 = Fusidic acid<br>33 = Gentamicin<br>34 = Imipenem<br>35 = Itraconazole<br>38 = Meropenem<br>58 = Methicillin<br>41 = Moxifloxacin<br>45 = Ofloxacin<br>46 = Penicillin/benzylpenicillin<br>47 = Piperacillin tazobactam (Tazocin)<br>48 = Posaconazole<br>49 = Rifampicin<br>52 = Ticarcillin clavulanate (Timentin)<br>53 = Tigecycline<br>54 = Tobramycin<br>56 = Vancomycin<br>57 = Voriconazole<br>999 = Other
**3.2**	Micro-organisms from Airway Samples	1.3.3.1a	antibiotic_type_other_3	Antibiotic type other 3		string			
**3.2**	Micro-organisms from Airway Samples	1.3.3.2	antibiotic_susceptibility_3	Antibiotic susceptibility 3		option			2 = Susceptible<br>3 = Intermediate<br>4 = Resistant<br>5 = Susceptible/Intermediate<br>6 = Intermediate/Resistant
**3.2**	Micro-organisms from Airway Samples	1.3.4.1	antibiotic_type_4	Antibiotic type 4		option			1 = Amoxicillin<br>2 = Amoxicillin clavulanate<br>4 = Amikacin<br>5 = Amphotericin B<br>6 = Ampicillin<br>7 = Azithromycin<br>8 = Aztreonam<br>12 = Cefepime<br>13 = Cefotaxime<br>14 = Ceftriaxone<br>15 = Ceftazidime<br>59 = Ceftazidime/avibactam<br>16 = Cephalexin<br>19 = Ciprofloxacin<br>21 = Clindamycin<br>22 = Colistin<br>23 = Cotrimoxazole (Trimethoprim and sulfamethoxaz<br>28 = Erythromycin<br>29 = Flucloxacillin<br>32 = Fusidic acid<br>33 = Gentamicin<br>34 = Imipenem<br>35 = Itraconazole<br>38 = Meropenem<br>58 = Methicillin<br>41 = Moxifloxacin<br>45 = Ofloxacin<br>46 = Penicillin/benzylpenicillin<br>47 = Piperacillin tazobactam (Tazocin)<br>48 = Posaconazole<br>49 = Rifampicin<br>52 = Ticarcillin clavulanate (Timentin)<br>53 = Tigecycline<br>54 = Tobramycin<br>56 = Vancomycin<br>57 = Voriconazole<br>999 = Other
**3.2**	Micro-organisms from Airway Samples	1.3.4.1a	antibiotic_type_other_4	Antibiotic type other 4		string			
**3.2**	Micro-organisms from Airway Samples	1.3.4.2	antibiotic_susceptibility_4	Antibiotic susceptibility 4		option			2 = Susceptible<br>3 = Intermediate<br>4 = Resistant<br>5 = Susceptible/Intermediate<br>6 = Intermediate/Resistant
**3.2**	Micro-organisms from Airway Samples	1.3.5.1	antibiotic_type_5	Antibiotic type 5		option			1 = Amoxicillin<br>2 = Amoxicillin clavulanate<br>4 = Amikacin<br>5 = Amphotericin B<br>6 = Ampicillin<br>7 = Azithromycin<br>8 = Aztreonam<br>12 = Cefepime<br>13 = Cefotaxime<br>14 = Ceftriaxone<br>15 = Ceftazidime<br>59 = Ceftazidime/avibactam<br>16 = Cephalexin<br>19 = Ciprofloxacin<br>21 = Clindamycin<br>22 = Colistin<br>23 = Cotrimoxazole (Trimethoprim and sulfamethoxaz<br>28 = Erythromycin<br>29 = Flucloxacillin<br>32 = Fusidic acid<br>33 = Gentamicin<br>34 = Imipenem<br>35 = Itraconazole<br>38 = Meropenem<br>58 = Methicillin<br>41 = Moxifloxacin<br>45 = Ofloxacin<br>46 = Penicillin/benzylpenicillin<br>47 = Piperacillin tazobactam (Tazocin)<br>48 = Posaconazole<br>49 = Rifampicin<br>52 = Ticarcillin clavulanate (Timentin)<br>53 = Tigecycline<br>54 = Tobramycin<br>56 = Vancomycin<br>57 = Voriconazole<br>999 = Other
**3.2**	Micro-organisms from Airway Samples	1.3.5.1a	antibiotic_type_other_5	Antibiotic type other 5		string			
**3.2**	Micro-organisms from Airway Samples	1.3.5.2	antibiotic_susceptibility_5	Antibiotic susceptibility 5		option			2 = Susceptible<br>3 = Intermediate<br>4 = Resistant<br>5 = Susceptible/Intermediate<br>6 = Intermediate/Resistant
**3.2**	Micro-organisms from Airway Samples	1.3.6.1	antibiotic_type_6	Antibiotic type 6		option			1 = Amoxicillin<br>2 = Amoxicillin clavulanate<br>4 = Amikacin<br>5 = Amphotericin B<br>6 = Ampicillin<br>7 = Azithromycin<br>8 = Aztreonam<br>12 = Cefepime<br>13 = Cefotaxime<br>14 = Ceftriaxone<br>15 = Ceftazidime<br>59 = Ceftazidime/avibactam<br>16 = Cephalexin<br>19 = Ciprofloxacin<br>21 = Clindamycin<br>22 = Colistin<br>23 = Cotrimoxazole (Trimethoprim and sulfamethoxaz<br>28 = Erythromycin<br>29 = Flucloxacillin<br>32 = Fusidic acid<br>33 = Gentamicin<br>34 = Imipenem<br>35 = Itraconazole<br>38 = Meropenem<br>58 = Methicillin<br>41 = Moxifloxacin<br>45 = Ofloxacin<br>46 = Penicillin/benzylpenicillin<br>47 = Piperacillin tazobactam (Tazocin)<br>48 = Posaconazole<br>49 = Rifampicin<br>52 = Ticarcillin clavulanate (Timentin)<br>53 = Tigecycline<br>54 = Tobramycin<br>56 = Vancomycin<br>57 = Voriconazole<br>999 = Other
**3.2**	Micro-organisms from Airway Samples	1.3.6.1a	antibiotic_type_other_6	Antibiotic type other 6		string			
**3.2**	Micro-organisms from Airway Samples	1.3.6.2	antibiotic_susceptibility_6	Antibiotic susceptibility 6		option			2 = Susceptible<br>3 = Intermediate<br>4 = Resistant<br>5 = Susceptible/Intermediate<br>6 = Intermediate/Resistant
**3.2**	Micro-organisms from Airway Samples	1.3.7.1	antibiotic_type_7	Antibiotic type 7		option			1 = Amoxicillin<br>2 = Amoxicillin clavulanate<br>4 = Amikacin<br>5 = Amphotericin B<br>6 = Ampicillin<br>7 = Azithromycin<br>8 = Aztreonam<br>12 = Cefepime<br>13 = Cefotaxime<br>14 = Ceftriaxone<br>15 = Ceftazidime<br>59 = Ceftazidime/avibactam<br>16 = Cephalexin<br>19 = Ciprofloxacin<br>21 = Clindamycin<br>22 = Colistin<br>23 = Cotrimoxazole (Trimethoprim and sulfamethoxaz<br>28 = Erythromycin<br>29 = Flucloxacillin<br>32 = Fusidic acid<br>33 = Gentamicin<br>34 = Imipenem<br>35 = Itraconazole<br>38 = Meropenem<br>58 = Methicillin<br>41 = Moxifloxacin<br>45 = Ofloxacin<br>46 = Penicillin/benzylpenicillin<br>47 = Piperacillin tazobactam (Tazocin)<br>48 = Posaconazole<br>49 = Rifampicin<br>52 = Ticarcillin clavulanate (Timentin)<br>53 = Tigecycline<br>54 = Tobramycin<br>56 = Vancomycin<br>57 = Voriconazole<br>999 = Other
**3.2**	Micro-organisms from Airway Samples	1.3.7.1a	antibiotic_type_other_7	Antibiotic type other 7		string			
**3.2**	Micro-organisms from Airway Samples	1.3.7.2	antibiotic_susceptibility_7	Antibiotic susceptibility 7		option			2 = Susceptible<br>3 = Intermediate<br>4 = Resistant<br>5 = Susceptible/Intermediate<br>6 = Intermediate/Resistant

**3.2**	Micro-organisms from Airway Samples	1.3.8.1	antibiotic_type_8	Antibiotic type 8		option			1 = Amoxicillin<br>2 = Amoxicillin clavulanate<br>4 = Amikacin<br>5 = Amphotericin B<br>6 = Ampicillin<br>7 = Azithromycin<br>8 = Aztreonam<br>12 = Cefepime<br>13 = Cefotaxime<br>14 = Ceftriaxone<br>15 = Ceftazidime<br>59 = Ceftazidime/avibactam<br>16 = Cephalexin<br>19 = Ciprofloxacin<br>21 = Clindamycin<br>22 = Colistin<br>23 = Cotrimoxazole (Trimethoprim and sulfamethoxa<br>28 = Erythromycin<br>29 = Flucloxacillin<br>32 = Fusidic acid<br>33 = Gentamicin<br>34 = Imipenem<br>35 = Itraconazole<br>38 = Meropenem<br>58 = Methicillin<br>41 = Moxifloxacin<br>45 = Ofloxacin<br>46 = Penicillin/benzylpenicillin<br>47 = Piperacillin tazobactam (Tazocin)<br>48 = Posaconazole<br>49 = Rifampicin<br>52 = Ticarcillin clavulanate (Timentin)<br>53 = Tigecycline<br>54 = Tobramycin<br>56 = Vancomycin<br>57 = Voriconazole<br>999 = Other
**3.2**	Micro-organisms from Airway Samples	1.3.8.1a	antibiotic_type_other_8	Antibiotic type other 8		string			
**3.2**	Micro-organisms from Airway Samples	1.3.8.2	antibiotic_susceptibility_8	Antibiotic susceptibility 8		option			2 = Susceptible<br>3 = Intermediate<br>4 = Resistant<br>5 = Susceptible/Intermediate<br>6 = Intermediate/Resistant
**3.2**	Micro-organisms from Airway Samples	1.3.9.1	antibiotic_type_9	Antibiotic type 9		option			1 = Amoxicillin<br>2 = Amoxicillin clavulanate<br>4 = Amikacin<br>5 = Amphotericin B<br>6 = Ampicillin<br>7 = Azithromycin<br>8 = Aztreonam<br>12 = Cefepime<br>13 = Cefotaxime<br>14 = Ceftriaxone<br>15 = Ceftazidime<br>59 = Ceftazidime/avibactam<br>16 = Cephalexin<br>19 = Ciprofloxacin<br>21 = Clindamycin<br>22 = Colistin<br>23 = Cotrimoxazole (Trimethoprim and sulfamethoxa<br>28 = Erythromycin<br>29 = Flucloxacillin<br>32 = Fusidic acid<br>33 = Gentamicin<br>34 = Imipenem<br>35 = Itraconazole<br>38 = Meropenem<br>58 = Methicillin<br>41 = Moxifloxacin<br>45 = Ofloxacin<br>46 = Penicillin/benzylpenicillin<br>47 = Piperacillin tazobactam (Tazocin)<br>48 = Posaconazole<br>49 = Rifampicin<br>52 = Ticarcillin clavulanate (Timentin)<br>53 = Tigecycline<br>54 = Tobramycin<br>56 = Vancomycin<br>57 = Voriconazole<br>999 = Other
**3.2**	Micro-organisms from Airway Samples	1.3.9.1a	antibiotic_type_other_9	Antibiotic type other 9		string			
**3.2**	Micro-organisms from Airway Samples	1.3.9.2	antibiotic_susceptibility_9	Antibiotic susceptibility 9		option			2 = Susceptible<br>3 = Intermediate<br>4 = Resistant<br>5 = Susceptible/Intermediate<br>6 = Intermediate/Resistant
**3.2**	Micro-organisms from Airway Samples	1.3.10.1	antibiotic_type_10	Antibiotic type 10		option			1 = Amoxicillin<br>2 = Amoxicillin clavulanate<br>4 = Amikacin<br>5 = Amphotericin B<br>6 = Ampicillin<br>7 = Azithromycin<br>8 = Aztreonam<br>12 = Cefepime<br>13 = Cefotaxime<br>14 = Ceftriaxone<br>15 = Ceftazidime<br>59 = Ceftazidime/avibactam<br>16 = Cephalexin<br>19 = Ciprofloxacin<br>21 = Clindamycin<br>22 = Colistin<br>23 = Cotrimoxazole (Trimethoprim and sulfamethoxa<br>28 = Erythromycin<br>29 = Flucloxacillin<br>32 = Fusidic acid<br>33 = Gentamicin<br>34 = Imipenem<br>35 = Itraconazole<br>38 = Meropenem<br>58 = Methicillin<br>41 = Moxifloxacin<br>45 = Ofloxacin<br>46 = Penicillin/benzylpenicillin<br>47 = Piperacillin tazobactam (Tazocin)<br>48 = Posaconazole<br>49 = Rifampicin<br>52 = Ticarcillin clavulanate (Timentin)<br>53 = Tigecycline<br>54 = Tobramycin<br>56 = Vancomycin<br>57 = Voriconazole<br>999 = Other
**3.2**	Micro-organisms from Airway Samples	1.3.10.1a	antibiotic_type_other_10	Antibiotic type other 10		string			
**3.2**	Micro-organisms from Airway Samples	1.3.10.2	antibiotic_susceptibility_10	Antibiotic susceptibility 10		option			2 = Susceptible<br>3 = Intermediate<br>4 = Resistant<br>5 = Susceptible/Intermediate<br>6 = Intermediate/Resistant
**3.2**	Micro-organisms from Airway Samples	1.3.11.1	antibiotic_type_11	Antibiotic type 11		option			1 = Amoxicillin<br>2 = Amoxicillin clavulanate<br>4 = Amikacin<br>5 = Amphotericin B<br>6 = Ampicillin<br>7 = Azithromycin<br>8 = Aztreonam<br>12 = Cefepime<br>13 = Cefotaxime<br>14 = Ceftriaxone<br>15 = Ceftazidime<br>59 = Ceftazidime/avibactam<br>16 = Cephalexin<br>19 = Ciprofloxacin<br>21 = Clindamycin<br>22 = Colistin<br>23 = Cotrimoxazole (Trimethoprim and sulfamethoxa<br>28 = Erythromycin<br>29 = Flucloxacillin<br>32 = Fusidic acid<br>33 = Gentamicin<br>34 = Imipenem<br>35 = Itraconazole<br>38 = Meropenem<br>58 = Methicillin<br>41 = Moxifloxacin<br>45 = Ofloxacin<br>46 = Penicillin/benzylpenicillin<br>47 = Piperacillin tazobactam (Tazocin)<br>48 = Posaconazole<br>49 = Rifampicin<br>52 = Ticarcillin clavulanate (Timentin)<br>53 = Tigecycline<br>54 = Tobramycin<br>56 = Vancomycin<br>57 = Voriconazole<br>999 = Other
**3.2**	Micro-organisms from Airway Samples	1.3.11.1a	antibiotic_type_other_11	Antibiotic type other 11		string			
**3.2**	Micro-organisms from Airway Samples	1.3.11.2	antibiotic_susceptibility_11	Antibiotic susceptibility 11		option			2 = Susceptible<br>3 = Intermediate<br>4 = Resistant<br>5 = Susceptible/Intermediate<br>6 = Intermediate/Resistant
**3.2**	Micro-organisms from Airway Samples	1.3.12.1	antibiotic_type_12	Antibiotic type 12		option			1 = Amoxicillin<br>2 = Amoxicillin clavulanate<br>4 = Amikacin<br>5 = Amphotericin B<br>6 = Ampicillin<br>7 = Azithromycin<br>8 = Aztreonam<br>12 = Cefepime<br>13 = Cefotaxime<br>14 = Ceftriaxone<br>15 = Ceftazidime<br>59 = Ceftazidime/avibactam<br>16 = Cephalexin<br>19 = Ciprofloxacin<br>21 = Clindamycin<br>22 = Colistin<br>23 = Cotrimoxazole (Trimethoprim and sulfamethoxa<br>28 = Erythromycin<br>29 = Flucloxacillin<br>32 = Fusidic acid<br>33 = Gentamicin<br>34 = Imipenem<br>35 = Itraconazole<br>38 = Meropenem<br>58 = Methicillin<br>41 = Moxifloxacin<br>45 = Ofloxacin<br>46 = Penicillin/benzylpenicillin<br>47 = Piperacillin tazobactam (Tazocin)<br>48 = Posaconazole<br>49 = Rifampicin<br>52 = Ticarcillin clavulanate (Timentin)<br>53 = Tigecycline<br>54 = Tobramycin<br>56 = Vancomycin<br>57 = Voriconazole<br>999 = Other
**3.2**	Micro-organisms from Airway Samples	1.3.12.1a	antibiotic_type_other_12	Antibiotic type other 12		string			
**3.2**	Micro-organisms from Airway Samples	1.3.12.2	antibiotic_susceptibility_12	Antibiotic susceptibility 12		option			2 = Susceptible<br>3 = Intermediate<br>4 = Resistant<br>5 = Susceptible/Intermediate<br>6 = Intermediate/Resistant

**3.2**	Micro-organisms from Airway Samples	1.3.13.1	antibiotic_type_13	Antibiotic type 13		option			1 = Amoxicillin<br>2 = Amoxicillin clavulanate<br>4 = Amikacin<br>5 = Amphotericin B<br>6 = Ampicillin<br>7 = Azithromycin<br>8 = Aztreonam<br>12 = Cefepime<br>13 = Cefotaxime<br>14 = Ceftriaxone<br>15 = Ceftazidime<br>59 = Ceftazidime/avibactam<br>16 = Cephalexin<br>19 = Ciprofloxacin<br>21 = Clindamycin<br>22 = Colistin<br>23 = Cotrimoxazole (Trimethoprim and sulfamethoxaz<br>28 = Erythromycin<br>29 = Flucloxacillin<br>32 = Fusidic acid<br>33 = Gentamicin<br>34 = Imipenem<br>35 = Itraconazole<br>38 = Meropenem<br>58 = Methicillin<br>41 = Moxifloxacin<br>45 = Ofloxacin<br>46 = Penicillin/benzylpenicillin<br>47 = Piperacillin tazobactam (Tazocin)<br>48 = Posaconazole<br>49 = Rifampicin<br>52 = Ticarcillin clavulanate (Timentin)<br>53 = Tigecycline<br>54 = Tobramycin<br>56 = Vancomycin<br>57 = Voriconazole<br>999 = Other
**3.2**	Micro-organisms from Airway Samples	1.3.13.1a	antibiotic_type_other_13	Antibiotic type other 13		string			
**3.2**	Micro-organisms from Airway Samples	1.3.13.2	antibiotic_susceptibility_13	Antibiotic susceptibility 13		option			2 = Susceptible<br>3 = Intermediate<br>4 = Resistant<br>5 = Susceptible/Intermediate<br>6 = Intermediate/Resistant
**3.2**	Micro-organisms from Airway Samples	1.3.14.1	antibiotic_type_14	Antibiotic type 14		option			1 = Amoxicillin<br>2 = Amoxicillin clavulanate<br>4 = Amikacin<br>5 = Amphotericin B<br>6 = Ampicillin<br>7 = Azithromycin<br>8 = Aztreonam<br>12 = Cefepime<br>13 = Cefotaxime<br>14 = Ceftriaxone<br>15 = Ceftazidime<br>59 = Ceftazidime/avibactam<br>16 = Cephalexin<br>19 = Ciprofloxacin<br>21 = Clindamycin<br>22 = Colistin<br>23 = Cotrimoxazole (Trimethoprim and sulfamethoxaz<br>28 = Erythromycin<br>29 = Flucloxacillin<br>32 = Fusidic acid<br>33 = Gentamicin<br>34 = Imipenem<br>35 = Itraconazole<br>38 = Meropenem<br>58 = Methicillin<br>41 = Moxifloxacin<br>45 = Ofloxacin<br>46 = Penicillin/benzylpenicillin<br>47 = Piperacillin tazobactam (Tazocin)<br>48 = Posaconazole<br>49 = Rifampicin<br>52 = Ticarcillin clavulanate (Timentin)<br>53 = Tigecycline<br>54 = Tobramycin<br>56 = Vancomycin<br>57 = Voriconazole<br>999 = Other
**3.2**	Micro-organisms from Airway Samples	1.3.14.1a	antibiotic_type_other_14	Antibiotic type other 14		string			
**3.2**	Micro-organisms from Airway Samples	1.3.14.2	antibiotic_susceptibility_14	Antibiotic susceptibility 14		option			2 = Susceptible<br>3 = Intermediate<br>4 = Resistant<br>5 = Susceptible/Intermediate<br>6 = Intermediate/Resistant
**3.2**	Micro-organisms from Airway Samples	1.3.15.1	antibiotic_type_15	Antibiotic type 15		option			1 = Amoxicillin<br>2 = Amoxicillin clavulanate<br>4 = Amikacin<br>5 = Amphotericin B<br>6 = Ampicillin<br>7 = Azithromycin<br>8 = Aztreonam<br>12 = Cefepime<br>13 = Cefotaxime<br>14 = Ceftriaxone<br>15 = Ceftazidime<br>59 = Ceftazidime/avibactam<br>16 = Cephalexin<br>19 = Ciprofloxacin<br>21 = Clindamycin<br>22 = Colistin<br>23 = Cotrimoxazole (Trimethoprim and sulfamethoxaz<br>28 = Erythromycin<br>29 = Flucloxacillin<br>32 = Fusidic acid<br>33 = Gentamicin<br>34 = Imipenem<br>35 = Itraconazole<br>38 = Meropenem<br>58 = Methicillin<br>41 = Moxifloxacin<br>45 = Ofloxacin<br>46 = Penicillin/benzylpenicillin<br>47 = Piperacillin tazobactam (Tazocin)<br>48 = Posaconazole<br>49 = Rifampicin<br>52 = Ticarcillin clavulanate (Timentin)<br>53 = Tigecycline<br>54 = Tobramycin<br>56 = Vancomycin<br>57 = Voriconazole<br>999 = Other
**3.2**	Micro-organisms from Airway Samples	1.3.15.1a	antibiotic_type_other_15	Antibiotic type other 15		string			
**3.2**	Micro-organisms from Airway Samples	1.3.15.2	antibiotic_susceptibility_15	Antibiotic susceptibility 15		option			2 = Susceptible<br>3 = Intermediate<br>4 = Resistant<br>5 = Susceptible/Intermediate<br>6 = Intermediate/Resistant
**3.2**	Micro-organisms from Airway Samples	1.3.16.1	antibiotic_type_16	Antibiotic type 16		option			1 = Amoxicillin<br>2 = Amoxicillin clavulanate<br>4 = Amikacin<br>5 = Amphotericin B<br>6 = Ampicillin<br>7 = Azithromycin<br>8 = Aztreonam<br>12 = Cefepime<br>13 = Cefotaxime<br>14 = Ceftriaxone<br>15 = Ceftazidime<br>59 = Ceftazidime/avibactam<br>16 = Cephalexin<br>19 = Ciprofloxacin<br>21 = Clindamycin<br>22 = Colistin<br>23 = Cotrimoxazole (Trimethoprim and sulfamethoxaz<br>28 = Erythromycin<br>29 = Flucloxacillin<br>32 = Fusidic acid<br>33 = Gentamicin<br>34 = Imipenem<br>35 = Itraconazole<br>38 = Meropenem<br>58 = Methicillin<br>41 = Moxifloxacin<br>45 = Ofloxacin<br>46 = Penicillin/benzylpenicillin<br>47 = Piperacillin tazobactam (Tazocin)<br>48 = Posaconazole<br>49 = Rifampicin<br>52 = Ticarcillin clavulanate (Timentin)<br>53 = Tigecycline<br>54 = Tobramycin<br>56 = Vancomycin<br>57 = Voriconazole<br>999 = Other
**3.2**	Micro-organisms from Airway Samples	1.3.16.1a	antibiotic_type_other_16	Antibiotic type other 16		string			
**3.2**	Micro-organisms from Airway Samples	1.3.16.2	antibiotic_susceptibility_16	Antibiotic susceptibility 16		option			2 = Susceptible<br>3 = Intermediate<br>4 = Resistant<br>5 = Susceptible/Intermediate<br>6 = Intermediate/Resistant
**3.2**	Micro-organisms from Airway Samples	1.3.17.1	antibiotic_type_17	Antibiotic type 17		option			1 = Amoxicillin<br>2 = Amoxicillin clavulanate<br>4 = Amikacin<br>5 = Amphotericin B<br>6 = Ampicillin<br>7 = Azithromycin<br>8 = Aztreonam<br>12 = Cefepime<br>13 = Cefotaxime<br>14 = Ceftriaxone<br>15 = Ceftazidime<br>59 = Ceftazidime/avibactam<br>16 = Cephalexin<br>19 = Ciprofloxacin<br>21 = Clindamycin<br>22 = Colistin<br>23 = Cotrimoxazole (Trimethoprim and sulfamethoxaz<br>28 = Erythromycin<br>29 = Flucloxacillin<br>32 = Fusidic acid<br>33 = Gentamicin<br>34 = Imipenem<br>35 = Itraconazole<br>38 = Meropenem<br>58 = Methicillin<br>41 = Moxifloxacin<br>45 = Ofloxacin<br>46 = Penicillin/benzylpenicillin<br>47 = Piperacillin tazobactam (Tazocin)<br>48 = Posaconazole<br>49 = Rifampicin<br>52 = Ticarcillin clavulanate (Timentin)<br>53 = Tigecycline<br>54 = Tobramycin<br>56 = Vancomycin<br>57 = Voriconazole<br>999 = Other
**3.2**	Micro-organisms from Airway Samples	1.3.17.1a	antibiotic_type_other_17	Antibiotic type other 17		string			
**3.2**	Micro-organisms from Airway Samples	1.3.17.2	antibiotic_susceptibility_17	Antibiotic susceptibility 17		option			2 = Susceptible<br>3 = Intermediate<br>4 = Resistant<br>5 = Susceptible/Intermediate<br>6 = Intermediate/Resistant

**3.2**	Micro-organisms from Airway Samples	1.3.18.1	antibiotic_type_18	Antibiotic type 18		option			1 = Amoxicillin<br>2 = Amoxicillin clavulanate<br>4 = Amikacin<br>5 = Amphotericin B<br>6 = Ampicillin<br>7 = Azithromycin<br>8 = Aztreonam<br>12 = Cefepime<br>13 = Cefotaxime<br>14 = Ceftriaxone<br>15 = Ceftazidime<br>59 = Ceftazidime/avibactam<br>16 = Cephalexin<br>19 = Ciprofloxacin<br>21 = Clindamycin<br>22 = Colistin<br>23 = Cotrimoxazole (Trimethoprim and sulfamethoxa<br>28 = Erythromycin<br>29 = Flucloxacillin<br>32 = Fusidic acid<br>33 = Gentamicin<br>34 = Imipenem<br>35 = Itraconazole<br>38 = Meropenem<br>58 = Methicillin<br>41 = Moxifloxacin<br>45 = Ofloxacin<br>46 = Penicillin/benzylpenicillin<br>47 = Piperacillin tazobactam (Tazocin)<br>48 = Posaconazole<br>49 = Rifampicin<br>52 = Ticarcillin clavulanate (Timentin)<br>53 = Tigecycline<br>54 = Tobramycin<br>56 = Vancomycin<br>57 = Voriconazole<br>999 = Other
**3.2**	Micro-organisms from Airway Samples	1.3.18.1a	antibiotic_type_other_18	Antibiotic type other 18		string			
**3.2**	Micro-organisms from Airway Samples	1.3.18.2	antibiotic_susceptibility_18	Antibiotic susceptibility 18		option			2 = Susceptible<br>3 = Intermediate<br>4 = Resistant<br>5 = Susceptible/Intermediate<br>6 = Intermediate/Resistant
**3.2**	Micro-organisms from Airway Samples	1.3.19.1	antibiotic_type_19	Antibiotic type 19		option			1 = Amoxicillin<br>2 = Amoxicillin clavulanate<br>4 = Amikacin<br>5 = Amphotericin B<br>6 = Ampicillin<br>7 = Azithromycin<br>8 = Aztreonam<br>12 = Cefepime<br>13 = Cefotaxime<br>14 = Ceftriaxone<br>15 = Ceftazidime<br>59 = Ceftazidime/avibactam<br>16 = Cephalexin<br>19 = Ciprofloxacin<br>21 = Clindamycin<br>22 = Colistin<br>23 = Cotrimoxazole (Trimethoprim and sulfamethoxa<br>28 = Erythromycin<br>29 = Flucloxacillin<br>32 = Fusidic acid<br>33 = Gentamicin<br>34 = Imipenem<br>35 = Itraconazole<br>38 = Meropenem<br>58 = Methicillin<br>41 = Moxifloxacin<br>45 = Ofloxacin<br>46 = Penicillin/benzylpenicillin<br>47 = Piperacillin tazobactam (Tazocin)<br>48 = Posaconazole<br>49 = Rifampicin<br>52 = Ticarcillin clavulanate (Timentin)<br>53 = Tigecycline<br>54 = Tobramycin<br>56 = Vancomycin<br>57 = Voriconazole<br>999 = Other
**3.2**	Micro-organisms from Airway Samples	1.3.19.1a	antibiotic_type_other_19	Antibiotic type other 19		string			
**3.2**	Micro-organisms from Airway Samples	1.3.19.2	antibiotic_susceptibility_19	Antibiotic susceptibility 19		option			2 = Susceptible<br>3 = Intermediate<br>4 = Resistant<br>5 = Susceptible/Intermediate<br>6 = Intermediate/Resistant
**3.2**	Micro-organisms from Airway Samples	1.3.20.1	antibiotic_type_20	Antibiotic type 20		option			1 = Amoxicillin<br>2 = Amoxicillin clavulanate<br>4 = Amikacin<br>5 = Amphotericin B<br>6 = Ampicillin<br>7 = Azithromycin<br>8 = Aztreonam<br>12 = Cefepime<br>13 = Cefotaxime<br>14 = Ceftriaxone<br>15 = Ceftazidime<br>59 = Ceftazidime/avibactam<br>16 = Cephalexin<br>19 = Ciprofloxacin<br>21 = Clindamycin<br>22 = Colistin<br>23 = Cotrimoxazole (Trimethoprim and sulfamethoxa<br>28 = Erythromycin<br>29 = Flucloxacillin<br>32 = Fusidic acid<br>33 = Gentamicin<br>34 = Imipenem<br>35 = Itraconazole<br>38 = Meropenem<br>58 = Methicillin<br>41 = Moxifloxacin<br>45 = Ofloxacin<br>46 = Penicillin/benzylpenicillin<br>47 = Piperacillin tazobactam (Tazocin)<br>48 = Posaconazole<br>49 = Rifampicin<br>52 = Ticarcillin clavulanate (Timentin)<br>53 = Tigecycline<br>54 = Tobramycin<br>56 = Vancomycin<br>57 = Voriconazole<br>999 = Other
**3.2**	Micro-organisms from Airway Samples	1.3.20.1a	antibiotic_type_other_20	Antibiotic type other 20		string			
**3.2**	Micro-organisms from Airway Samples	1.3.20.2	antibiotic_susceptibility_20	Antibiotic susceptibility 20		option			2 = Susceptible<br>3 = Intermediate<br>4 = Resistant<br>5 = Susceptible/Intermediate<br>6 = Intermediate/Resistant
**4**	Contraindications to Study Antibiotics	1.1	contraabx	Name of potentially contraindicated study antibiotic		option			2 = Amoxicillin clavulanate<br>4 = Amikacin<br>5 = Amphotericin B<br>6 = Ampicillin<br>7 = Azithromycin<br>8 = Aztreonam<br>9 = Caspofungin<br>10 = Cefaclor<br>11 = Cefazolin<br>12 = Cefepime<br>13 = Cefotaxime<br>14 = Ceftriaxone<br>15 = Ceftazidime<br>59 = Ceftazidime/avibactam<br>16 = Cephalexin<br>17 = Cephalothin<br>18 = Chloramphenicol<br>19 = Ciprofloxacin<br>20 = Clarithromycin<br>21 = Clindamycin<br>22 = Colistin<br>23 = Cotrimoxazole (Trimethoprim and sulfamethoxa<br>24 = Daptomycin<br>25 = Dicloxacillin<br>26 = Doxycycline<br>27 = Ertapenem<br>28 = Erythromycin<br>29 = Flucloxacillin<br>30 = Fluconazole<br>31 = Fosfomycin<br>32 = Fusidic acid<br>33 = Gentamicin<br>34 = Imipenem<br>35 = Itraconazole<br>36 = Ketoconazole<br>37 = Linezolid<br>38 = Meropenem<br>39 = Metronidazole<br>41 = Moxifloxacin<br>42 = Mupirocin<br>43 = Nitrofurantoin<br>44 = Norfloxacin<br>45 = Ofloxacin<br>46 = Penicillin/benzylpenicillin<br>47 = Piperacillin tazobactam (Tazocin)<br>48 = Posaconazole<br>49 = Rifampicin<br>50 = Teicoplanin<br>51 = Tetracycline<br>52 = Ticarcillin clavulanate (Timentin)<br>53 = Tigecycline<br>54 = Tobramycin<br>55 = Trimethoprim<br>56 = Vancomycin<br>57 = Voriconazole
**4**	Contraindications to Study Antibiotics	1.1.1a	contrarouteiv	Intravenous		checkbox		0	1 = Yes<br>0 = No
**4**	Contraindications to Study Antibiotics	1.1.1b	contrarouteinh	Inhaled		checkbox		0	1 = Yes<br>0 = No
**4**	Contraindications to Study Antibiotics	1.2	contrarsn	What is the reason for the contraindication to the antibiotic named in 1.1?		option			1 = Previous immune-mediated reaction<br>2 = Susceptibility to toxicity
**4**	Contraindications to Study Antibiotics	1.2.1	contracause	Is the antibiotic named in 1.1 the specific antibiotic that the patient reacted to?		boolean			1 = Yes<br>0 = No

4	Contraindications to Study Antibiotics	1.2.1.1	contraparent	Which antibiotic did the patient react to that resulted in this contraindication?		option			1 = Amoxicillin<br>2 = Amoxicillin clavulanate<br>4 = Amikacin<br>5 = Amphotericin B<br>6 = Ampicillin<br>7 = Azithromycin<br>8 = Aztreonam<br>9 = Caspofungin<br>10 = Cefaclor<br>11 = Cefazolin<br>12 = Cefepime<br>13 = Cefotaxime<br>14 = Ceftriaxone<br>15 = Ceftazidime<br>59 = Ceftazidime/avibactam<br>16 = Cephalexin<br>17 = Cephalothin<br>18 = Chloramphenicol<br>19 = Ciprofloxacin<br>20 = Clarithromycin<br>21 = Clindamycin<br>22 = Colistin<br>23 = Cotrimoxazole (Trimethoprim and sulfamethoxaz<br>24 = Daptomycin<br>25 = Dicloxacillin<br>26 = Doxycycline<br>27 = Ertapenem<br>28 = Erythromycin<br>29 = Flucloxacillin<br>30 = Fluconazole<br>31 = Fosfomycin<br>32 = Fusidic acid<br>33 = Gentamicin<br>34 = Imipenem<br>35 = Itraconazole<br>36 = Ketoconazole<br>37 = Linezolid<br>38 = Meropenem<br>58 = Methicillin<br>39 = Metronidazole<br>40 = Minocycline<br>41 = Moxifloxacin<br>42 = Mupirocin<br>43 = Nitrofurantoin<br>44 = Norfloxacin<br>45 = Ofloxacin<br>46 = Penicillin/benzylpenicillin<br>47 = Piperacillin tazobactam (Tazocin)<br>48 = Posaconazole<br>49 = Rifampicin<br>50 = Teicoplanin<br>51 = Tetracycline<br>52 = Ticarcillin clavulanate (Timentin)<br>53 = Tigecycline<br>54 = Tobramycin<br>55 = Trimethoprim<br>56 = Vancomycin<br>57 = Voriconazole<br>999 = Other
4	Contraindications to Study Antibiotics	1.2.1.1a	contraparentoth	Specify		string			
4	Contraindications to Study Antibiotics	1.3a	contrasrcpat	Patient/parent		checkbox		0	1 = Yes<br>0 = No
4	Contraindications to Study Antibiotics	1.3b	contrasrcalrt	Hospital alert		checkbox		0	1 = Yes<br>0 = No
4	Contraindications to Study Antibiotics	1.3c	contrasrcclin	Clinician		checkbox		0	1 = Yes<br>0 = No
4	Contraindications to Study Antibiotics	1.3d	contrasrcbrc	Medical alert bracelet		checkbox		0	1 = Yes<br>0 = No
4	Contraindications to Study Antibiotics	1.3e	contrasrcrev	Medical record review		checkbox		0	1 = Yes<br>0 = No
4	Contraindications to Study Antibiotics	1.3f	contrasrcoth	Other		checkbox		0	1 = Yes<br>0 = No
4	Contraindications to Study Antibiotics	1.3.1	contrasrcothdesc	Other source		string			
4	Contraindications to Study Antibiotics	2.1	reactknown	Are details of this reaction known?		boolean			1 = Yes<br>0 = No
4	Contraindications to Study Antibiotics	2.1.1	reacttype	Type of reaction		option			1 = Anaphylaxis<br>2 = Hepatitis<br>3 = Acute interstitial nephritis<br>4 = Serum sickness<br>5 = Drug Reaction with Eosinophilia and Systemic Sy<br>6 = Stevens-Johnson-Syndrome<br>7 = Toxic Epidermal Necrolysis<br>8 = Acute Generalised Exanthematous Pustulosis<br>9 = Other serious reaction<br>10 = Less serious reaction
4	Contraindications to Study Antibiotics	2.1.1a	reacttypeoth	Specify		string			
4	Contraindications to Study Antibiotics	2.1.1.1	reactsimm	Immediacy of reaction		option			1 = Immediate (< 6hr after dose)<br>2 = Non-immediate<br>3 = Unknown
4	Contraindications to Study Antibiotics	2.1.1.2	reactallergic	Reaction considered to be		option			1 = Likely allergic<br>2 = Unlikely allergic and not a contraindication for fut
4	Contraindications to Study Antibiotics	2.1.2	reactdate	Reaction start date		date			
4	Contraindications to Study Antibiotics	2.1.3	reactassess	Has the reaction assessed by an immunologist?		boolean			1 = Yes<br>0 = No
4	Contraindications to Study Antibiotics	2.1.3.1	reactassessdat	Date of assessment		date			
4	Contraindications to Study Antibiotics	2.1.3.2	contrasafe	With regards to safety of %{contraabx}, what did the immunologist determine?		option			1 = Contraindicated<br>0 = Not contraindicated<br>-1 = Not determined
4	Contraindications to Study Antibiotics	3.1	contraactive	Does the responsible clinician consider this antibiotic to be currently contraindicated for this participant?		option			1 = Contraindicated<br>0 = Not contraindicated<br>-1 = To be confirmed
5	Concomitant Medications	1.1	cmtrt	Name of medicine		option			2 = Amoxicillin clavulanate<br>7 = Azithromycin<br>16 = Cephalexin<br>19 = Ciprofloxacin<br>21 = Clindamycin<br>23 = Cotrimoxazole (Trimethoprim and sulfamethoxaz<br>29 = Flucloxacillin<br>35 = Itraconazole<br>40 = Minocycline<br>54 = Tobramycin<br>57 = Voriconazole<br>1001 = Amphotericin B<br>1002 = Colecalciferol (Vitamin D)<br>1003 = Creon<br>1004 = Dornase Alfa (Pulmozyme®)<br>1005 = Esomeprazole<br>1006 = Mannitol (Bronchitol®)<br>1007 = Mometasone (Nasonex®)<br>1008 = Omeprazole<br>1009 = Salbutamol (Ventolin®)<br>1010 = Sodium chloride (salt)<br>1012 = Vitamin ABDECK capsules<br>1013 = Tezacaftor/ivacaftor and ivacaftor (symdeko®<br>1014 = Ivacaftor (Kalydeco®)<br>1015 = Lumacaftor/ivacaftor (Orkambi®)<br>1016 = Hypertonic saline [RETIRED]<br>1017 = Fluticasone propionate and salmeterol xinafoa<br>1018 = Fluticasone proprionate (Flixotide)<br>1019 = Ciclesonide (Alvesco)<br>1020 = Beclometasone diproprionate (QVAR)<br>1021 = Symbicort (Budesomide/Formoterol)<br>1022 = BREO (Fluticasone/Vilanterol)<br>1023 = Hypertonic saline 6%<br>1024 = Hypertonic saline 3%<br>1025 = Sodium chloride 0.9%<br>1026 = Elexacaftor/tezacaftor/ivacaftor (Trikafta)<br>9998 = Other study medication<br>9999 = Other
5	Concomitant Medications	1.1a	cmtrtoth	Name of other medicine		string			
5	Concomitant Medications	1.1.0	cmtrtname	Medication generic name as text		computed			
5	Concomitant Medications	1.2	cmstdat	Start date		date			
5	Concomitant Medications	1.3	cmongo	Ongoing		boolean			1 = Yes<br>0 = No
5	Concomitant Medications	1.4	cmendat	Stop date		date			
5	Concomitant Medications	1.5	cmdosefrq	Frequency		option			1 = Daily<br>2 = Twice a day<br>3 = Three times a day<br>4 = Four times a day<br>5 = Every second day<br>6 = Three times per week<br>7 = Weekly<br>8 = Every 2 weeks<br>9 = Monthly<br>10 = Alternate months<br>11 = As needed<br>12 = Continuous infusion<br>13 = Unknown<br>14 = Other
5	Concomitant Medications	1.2	cmroute	Route		option			1 = Inhalation<br>2 = Intramuscular<br>3 = Intravenous<br>4 = Oral (or gastrostomy)<br>5 = Subcutaneous<br>6 = Topical<br>7 = Other<br>99 = Not known
6	Intensive Therapy Commencement - Da	1.1	itherstdat	Admission date for intensive therapy		date			
6	Intensive Therapy Commencement - Day 0		itherenddat	Intensive therapy end date		computed			
6	Intensive Therapy Commencement - Da	1.2	itherloc	Admission location (where intensive therapy will begin)		option			1 = Hospital<br>2 = HITH
6	Intensive Therapy Commencement - Da	1.2.1	itherhosp	Hospital		option			1 = Current hospital<br>2 = Regional hospital
6	Intensive Therapy Commencement - Da	1.2.2	admitweight	Weight		decimal	kg		
6	Intensive Therapy Commencement - Da	1.3	itrand	Will the participant be randomised for this intensive therapy?		boolean			1 = Yes<br>0 = No
6	Intensive Therapy Commencement - Da	1.3.1	itnorandreas	Reason for the exacerbation not being randomised		option			1 = Patient choice<br>2 = Clinician choice<br>3 = Logistics<br>9 = Other
6	Intensive Therapy Commencement - Da	1.3.2	itnorandexp	Briefly explain the reason (this will help interpretation of the generalisability of any results from BEAT CF randomisations)		text			
6	Intensive Therapy Commencement - Da	2.1	admitcoughtp	Cough type		option			1 = Wet<br>2 = Dry
6	Intensive Therapy Commencement - Da	2.1a	admitcoughtpna	Not done		checkbox		0	1 = Yes<br>0 = No
6	Intensive Therapy Commencement - Da	2.2	admitcrepit	Crepitations		boolean			1 = Yes<br>0 = No
6	Intensive Therapy Commencement - Da	2.2a	admitcrepitna	Not done		checkbox		0	1 = Yes<br>0 = No
6	Intensive Therapy Commencement - Da	3.1	itplanabx	Antibiotics?		boolean			1 = Yes<br>0 = No
6	Intensive Therapy Commencement - Da	3.2	itplanit	Inhaled therapies?		boolean			1 = Yes<br>0 = No
6	Intensive Therapy Commencement - Da	3.3	itplanai	Anti-inflammatory treatments?		boolean			1 = Yes<br>0 = No
6	Intensive Therapy Commencement - Da	3.4	itplanph	Physiotherapy?		boolean			1 = Yes<br>0 = No
7.1	Antibiotic Treatment		intensive_therapy_id	Intensive therapy ID		linked_form			

**7.1**	Antibiotic Treatment	1.1	abxname	Generic name		option			2 = Amoxicillin clavulanate<br>4 = Amikacin<br>5 = Amphotericin B<br>6 = Ampicillin<br>7 = Azithromycin<br>8 = Aztreonam<br>9 = Caspofungin<br>10 = Cefaclor<br>11 = Cefazolin<br>12 = Cefepime<br>13 = Cefotaxime<br>14 = Ceftriaxone<br>15 = Ceftazidime<br>59 = Ceftazidime/avibactam<br>16 = Cephalexin<br>17 = Cephalothin<br>18 = Chloramphenicol<br>19 = Ciprofloxacin<br>20 = Clarithromycin<br>21 = Clindamycin<br>22 = Colistin<br>23 = Cotrimoxazole (Trimethoprim and sulfamethoxaz<br>24 = Daptomycin<br>25 = Dicloxacillin<br>26 = Doxycycline<br>27 = Ertapenem<br>28 = Erythromycin<br>29 = Flucloxacillin<br>30 = Fluconazole<br>31 = Fosfomycin<br>32 = Fusidic acid<br>33 = Gentamicin<br>34 = Imipenem<br>35 = Itraconazole<br>36 = Ketoconazole<br>37 = Linezolid<br>38 = Meropenem<br>39 = Metronidazole<br>41 = Moxifloxacin<br>42 = Mupirocin<br>43 = Nitrofurantoin<br>44 = Norfloxacin<br>45 = Ofloxacin<br>46 = Penicillin/benzylpenicillin<br>47 = Piperacillin tazobactam (Tazocin)<br>48 = Posaconazole<br>49 = Rifampicin<br>50 = Teicoplanin<br>51 = Tetracycline<br>52 = Ticarcillin clavulanate (Timentin)<br>53 = Tigecycline<br>54 = Tobramycin<br>55 = Trimethoprim<br>56 = Vancomycin<br>57 = Voriconazole
**7.1**	Antibiotic Treatment	1.2	abxtype	Type		option			1 = Backbone<br>2 = Adjunct
**7.1**	Antibiotic Treatment	1.3	abxstartdatm	Start date/time		datetime			
**7.1**	Antibiotic Treatment	1.4	abxdose	Dose		decimal			
**7.1**	Antibiotic Treatment	1.4a	abxunit	Unit		option			1 = milligrams (mg)<br>2 = millilitres (mL)<br>3 = micrograms (mcg)<br>4 = (International) units
**7.1**	Antibiotic Treatment	1.5	abxroute	Route		option			1 = Inhalation<br>2 = Intramuscular<br>3 = Intravenous<br>4 = Oral (or gastrostomy)<br>5 = Subcutaneous<br>6 = Topical
**7.1**	Antibiotic Treatment	1.6	abxfreq	Frequency		option			1 = Daily<br>2 = Twice a day<br>3 = Three times a day<br>4 = Four times a day<br>5 = Every second day<br>6 = Monthly<br>7 = As needed<br>8 = Continuous infusion<br>9 = Unknown<br>10 = Other
**7.1**	Antibiotic Treatment	1.6.1	abxdur	Duration		option			1 = Less than 1 hour<br>2 = 1 or more but less than 6 hours<br>3 = 6 or more hours
**7.1**	Antibiotic Treatment	1.7	abxstopdatm	Stop date/time		datetime			
**7.1**	Antibiotic Treatment	1.7.1	abxstoprsn	Reason antibiotic stopped		option			1 = Completed<br>2 = Unsatisfactory response<br>3 = Adverse reaction<br>4 = New micro results<br>5 = Not available<br>7 = Dose adjustment<br>6 = Other
**7.1**	Antibiotic Treatment	1.7.1a	abxstoprsnoth	Specify		string			
**7.1**	Antibiotic Treatment	1.7.2	abxmissdoses	Number of missed doses during this course of antibiotics		integer			
**7.1**	Antibiotic Treatment	1.7.2.1	abxmissdatm1	Date and time		datetime			
**7.1**	Antibiotic Treatment	1.7.2.2	abxmissdatm2	Date and time		datetime			
**7.1**	Antibiotic Treatment	1.7.2.3	abxmissdatm3	Date and time		datetime			
**7.1**	Antibiotic Treatment	1.7.2.4	abxmissdatm4	Date and time		datetime			
**7.1**	Antibiotic Treatment	1.7.2.5	abxmissdatm5	Date and time		datetime			
**7.1**	Antibiotic Treatment	1.7.2.6	abxmissdatm6	Date and time		datetime			
**7.2**	Inhaled Therapy Treatment		intensive_therapy_id	Intensive therapy ID		linked_form			
**7.2**	Inhaled Therapy Treatment	1.1	ittrt	Generic name		option			1 = Dornase alpha (Pulmozyme)<br>2 = Mannitol (bronchitol)<br>3 = Hypertonic saline<br>4 = Salbutamol<br>5 = Fluticasone propionate and salmeterol xinafoate (<br>6 = Fluticasone proprionate (Flixotide)<br>7 = Ciclesonide (Alvesco)<br>8 = Beclometasone dipropionate (QVAR)<br>9 = Symbicort (Budesomide/Formoterol)<br>10 = BREO (Fluticasone/Vilanterol)<br>99 = Other
**7.2**	Inhaled Therapy Treatment	1.1a	ittrtoth	Specify		string			
**7.2**	Inhaled Therapy Treatment	1.2	itstartdat	Start date		date			
**7.2**	Inhaled Therapy Treatment	1.3	itdose	Dose		decimal			
**7.2**	Inhaled Therapy Treatment	1.4	itdosu	Units		option			1 = milligrams (mg)<br>2 = millilitres (mL)<br>3 = micrograms (mcg)<br>4 = (International) units
**7.2**	Inhaled Therapy Treatment	1.5	itfreq	Frequency		option			1 = Daily<br>2 = Twice a day<br>3 = Three times a day<br>4 = Four times a day<br>5 = Every second day<br>6 = Three times per week<br>7 = Weekly<br>8 = Every 2 weeks<br>9 = Monthly<br>10 = Alternate months<br>11 = As needed<br>12 = Continuous infusion<br>13 = Unknown<br>14 = Other
**7.2**	Inhaled Therapy Treatment	1.6	itstopdat	Stop date		date			
**7.3**	Anti-inflammatory Treatment		intensive_therapy_id	Intensive therapy ID		linked_form			
**7.3**	Anti-inflammatory Treatment	1.1	aitrt	Generic name		option			1 = Azithromycin<br>2 = Prednisolone<br>3 = NSAID
**7.3**	Anti-inflammatory Treatment	1.1a	aitrtnsaid	Generic name		string			
**7.3**	Anti-inflammatory Treatment	1.2	aistartdat	Start date		date			
**7.3**	Anti-inflammatory Treatment	1.3	aidose	Dose		decimal			
**7.3**	Anti-inflammatory Treatment	1.4	aidosu	Dose Units		option			1 = milligrams (mg)<br>2 = millilitres (mL)<br>3 = micrograms (mcg)<br>4 = (International) units
**7.3**	Anti-inflammatory Treatment	1.5	aifreq	Frequency		option			1 = Daily<br>2 = Twice a day<br>3 = Three times a day<br>4 = Four times a day<br>5 = Every second day<br>6 = Three times per week<br>7 = Weekly<br>8 = Every 2 weeks<br>9 = Monthly<br>10 = Alternate months<br>11 = As needed<br>12 = Continuous infusion<br>13 = Unknown<br>14 = Other
**7.3**	Anti-inflammatory Treatment	1.6	aistopdat	Stop date		date			
**7.4**	Physiotherapy Treatment		intensive_therapy_id	Intensive therapy ID		linked_form			
**7.4**	Physiotherapy Treatment	1.1	phassdat	Date Assessed		date			
**7.4**	Physiotherapy Treatment	1.2	phasswk	Week of treatment		integer			
**7.4**	Physiotherapy Treatment	2.1	phactacbt	ACBT (Active Cycle of breathing technique)		boolean			1 = Yes<br>0 = No
**7.4**	Physiotherapy Treatment	2.1.1	phactacbtsess	Total number of physio sessions in past 7 days		integer			
**7.4**	Physiotherapy Treatment	2.1.2	phactacbtdur	Average duration of treatment per session		option	minutes		1 = 1-10<br>2 = 11-20<br>3 = 21-30<br>4 = 31-40<br>5 = > 40
**7.4**	Physiotherapy Treatment	2.1.3	phactacbtsup	Number of physio sessions supervised in the past 7 days		integer			
**7.4**	Physiotherapy Treatment	2.1.4	phactacbtinh	Was inhalation combined with this ACT (not before)?		boolean			1 = Yes<br>0 = No
**7.4**	Physiotherapy Treatment	2.2	phactaad	AD/AAD (Autogenic Drainage/Assisted)		boolean			1 = Yes<br>0 = No
**7.4**	Physiotherapy Treatment	2.2.1	phactaadsess	Total number of physio sessions in the past 7 days		integer			
**7.4**	Physiotherapy Treatment	2.2.2	phactaaddur	Average duration of treatment per session		option	minutes		1 = 1-10<br>2 = 11-20<br>3 = 21-30<br>4 = 31-40<br>5 = > 40

7.4	Physiotherapy Treatment	2.2.3	phactaadsup	Number of physio sessions supervised in the past 7 days		integer			
7.4	Physiotherapy Treatment	2.2.4	phactaadinh	Was inhalation combined with this ACT (not before)?		boolean			1 = Yes<br>0 = No
7.4	Physiotherapy Treatment	2.3	phactpep	PEP (Positive Expiratory Pressure)		boolean			1 = Yes<br>0 = No
7.4	Physiotherapy Treatment	2.3.1	phactpepsess	Total number of physio sessions in the past 7 days		integer			
7.4	Physiotherapy Treatment	2.3.2	phactpepdur	Average duration of treatment per session		option	minutes		1 = 1-10<br>2 = 11-20<br>3 = 21-30<br>4 = 31-40<br>5 = > 40
7.4	Physiotherapy Treatment	2.3.3	phactpepsup	Number of physio sessions supervised in the past 7 days		integer			
7.4	Physiotherapy Treatment	2.3.4	phactpepinh	Was inhalation combined with this ACT (not before)?		boolean			1 = Yes<br>0 = No
7.4	Physiotherapy Treatment	2.4	phactopep	Osc-PEP (Oscillating Positive Expiratory Pressure)		boolean			1 = Yes<br>0 = No
7.4	Physiotherapy Treatment	2.4.1	phactopepsess	Total number of physio sessions in the past 7 days		integer			
7.4	Physiotherapy Treatment	2.4.2	phactopepdur	Average duration of treatment per session		option	minutes		1 = 1-10<br>2 = 11-20<br>3 = 21-30<br>4 = 31-40<br>5 = > 40
7.4	Physiotherapy Treatment	2.4.3	phactopepsup	Number of physio sessions supervised in the past 7 days		integer			
7.4	Physiotherapy Treatment	2.4.4	phactopepinh	Was inhalation combined with this ACT (not before)?		boolean			1 = Yes<br>0 = No
7.4	Physiotherapy Treatment	2.5	phactbpep	Bubble/bottle PEP		boolean			1 = Yes<br>0 = No
7.4	Physiotherapy Treatment	2.5.1	phactbpepsess	Total number of physio sessions in the past 7 days		integer			
7.4	Physiotherapy Treatment	2.5.2	phactbpepdur	Average duration of treatment per session		option	minutes		1 = 1-10<br>2 = 11-20<br>3 = 21-30<br>4 = 31-40<br>5 = > 40
7.4	Physiotherapy Treatment	2.5.3	phactbpepsup	Number of physio sessions supervised in the past 7 days		integer			
7.4	Physiotherapy Treatment	2.5.4	phactbpepinh	Was inhalation combined with this ACT (not before)?		boolean			1 = Yes<br>0 = No
7.4	Physiotherapy Treatment	2.6	phactniv	Non-invasive ventilation		boolean			1 = Yes<br>0 = No
7.4	Physiotherapy Treatment	2.6.1	phactnivsess	Total number of physio sessions in the past 7 days		integer			
7.4	Physiotherapy Treatment	2.6.2	phactnivdur	Average duration of treatment per session		option	minutes		1 = 1-10<br>2 = 11-20<br>3 = 21-30<br>4 = 31-40<br>5 = > 40
7.4	Physiotherapy Treatment	2.6.3	phactnivsup	Number of physio sessions supervised in the past 7 days		integer			
7.4	Physiotherapy Treatment	2.6.4	phactnivinh	Was inhalation combined with this ACT (not before)?		boolean			1 = Yes<br>0 = No
7.4	Physiotherapy Treatment	2.7	phactman	Manual techniques		boolean			1 = Yes<br>0 = No
7.4	Physiotherapy Treatment	2.7.1	phactmansess	Total number of physio sessions in the past 7 days		integer			
7.4	Physiotherapy Treatment	2.7.2	phactmandur	Average duration of treatment per session		option	minutes		1 = 1-10<br>2 = 11-20<br>3 = 21-30<br>4 = 31-40<br>5 = > 40
7.4	Physiotherapy Treatment	2.7.3	phactmansup	Number of physio sessions supervised in the past 7 days		integer			
7.4	Physiotherapy Treatment	2.7.4	phactmaninh	Was inhalation combined with this ACT (not before)?		boolean			1 = Yes<br>0 = No
7.4	Physiotherapy Treatment	2.8	phactvest	Vest (High frequency chest wall oscillation)		boolean			1 = Yes<br>0 = No
7.4	Physiotherapy Treatment	2.8.1	phactvestsess	Total number of physio sessions in the past 7 days		integer			
7.4	Physiotherapy Treatment	2.8.2	phactvestdur	Average duration of treatment per session		option	minutes		1 = 1-10<br>2 = 11-20<br>3 = 21-30<br>4 = 31-40<br>5 = > 40
7.4	Physiotherapy Treatment	2.8.3	phactvestsup	Number of physio sessions supervised in the past 7 days		integer			
7.4	Physiotherapy Treatment	2.8.4	phactvestinh	Was inhalation combined with this ACT (not before)?		boolean			1 = Yes<br>0 = No
7.4	Physiotherapy Treatment	2.9	phactexec	Exercise		boolean			1 = Yes<br>0 = No
7.4	Physiotherapy Treatment	2.9.1	phactexecsess	Total number of physio sessions in the past 7 days		integer			
7.4	Physiotherapy Treatment	2.9.2	phactexecdur	Average duration of treatment per session		option	minutes		1 = 1-10<br>2 = 11-20<br>3 = 21-30<br>4 = 31-40<br>5 = > 40
7.4	Physiotherapy Treatment	2.9.3	phactexecsup	Number of physio sessions supervised in the past 7 days		integer			
7.4	Physiotherapy Treatment	2.9.4	phactexecinh	Was inhalation combined with this ACT (not before)?		boolean			1 = Yes<br>0 = No
7.4	Physiotherapy Treatment	2.10	phactmetaneb	Metaneb		boolean			1 = Yes<br>0 = No
7.4	Physiotherapy Treatment	2.10.1	phactmetanebsess	Total number of physio sessions in the past 7 days		integer			
7.4	Physiotherapy Treatment	2.10.2	phactmetanebdur	Average duration of treatment per session		option	minutes		1 = 1-10<br>2 = 11-20<br>3 = 21-30<br>4 = 31-40<br>5 = > 40
7.4	Physiotherapy Treatment	2.10.3	phactmetanebsup	Number of physio sessions supervised in the past 7 days		integer			
7.4	Physiotherapy Treatment	2.10.4	phactmetanebinh	Was inhalation combined with this ACT (not before)?		boolean			1 = Yes<br>0 = No
7.4	Physiotherapy Treatment	2.11	phactoth	Other		boolean			1 = Yes<br>0 = No
7.4	Physiotherapy Treatment	2.11.1	phactothspec	Specify		string			
7.4	Physiotherapy Treatment	2.11.2	phactothsess	Total number of physio sessions in the past 7 days		integer			
7.4	Physiotherapy Treatment	2.11.3	phactothdur	Average duration of treatment per session		option	minutes		1 = 1-10<br>2 = 11-20<br>3 = 21-30<br>4 = 31-40<br>5 = > 40
7.4	Physiotherapy Treatment	2.11.4	phactothsup	Number of physio sessions supervised in the past 7 days		integer			
7.4	Physiotherapy Treatment	2.11.5	phactothinh	Was inhalation combined with this ACT (not before)?		boolean			1 = Yes<br>0 = No
7.4	Physiotherapy Treatment	3.1	phexeaero	Aerobic		boolean			1 = Yes<br>0 = No
7.4	Physiotherapy Treatment	3.1.1	phexeaerosess	Total number of physio sessions in the past 7 days		integer			
7.4	Physiotherapy Treatment	3.1.2	phexeaerodur	Average duration of exercise per session		option	minutes		1 = 1-10<br>2 = 11-20<br>3 = 21-30<br>4 = 31-40<br>5 = > 40
7.4	Physiotherapy Treatment	3.2	phexeresist	Resistance		boolean			1 = Yes<br>0 = No
7.4	Physiotherapy Treatment	3.2.1	phexeresistsess	Total number of physio sessions in the past 7 days		integer			
7.4	Physiotherapy Treatment	3.2.2	phexeresistdur	Average duration of exercise per session		option	minutes		1 = 1-10<br>2 = 11-20<br>3 = 21-30<br>4 = 31-40<br>5 = > 40
7.4	Physiotherapy Treatment	3.3	phexehiit	High intensity interval training		boolean			1 = Yes<br>0 = No
7.4	Physiotherapy Treatment	3.3.1	phexehiitsess	Total number of physio sessions in the past 7 days		integer			
7.4	Physiotherapy Treatment	3.3.2	phexehiitdur	Average duration of exercise per session		option	minutes		1 = 1-10<br>2 = 11-20<br>3 = 21-30<br>4 = 31-40<br>5 = > 40
7.4	Physiotherapy Treatment	3.4	phexeplay	Facilitated activity/play		boolean			1 = Yes<br>0 = No
7.4	Physiotherapy Treatment	3.4.1	phexeplaysess	Total number of physio sessions in the past 7 days		integer			
7.4	Physiotherapy Treatment	3.4.2	phexeplaydur	Average duration of exercise per session		option	minutes		1 = 1-10<br>2 = 11-20<br>3 = 21-30<br>4 = 31-40<br>5 = > 40
7.4	Physiotherapy Treatment	3.5	phexeoth	Other		boolean			1 = Yes<br>0 = No
7.4	Physiotherapy Treatment	3.5.1	phexeothspec	Specify		string			
7.4	Physiotherapy Treatment	3.5.2	phexeothsess	Total number of physio sessions in the past 7 days		integer			
7.4	Physiotherapy Treatment	3.5.3	phexeothdur	Average duration of exercise per session		option	minutes		1 = 1-10<br>2 = 11-20<br>3 = 21-30<br>4 = 31-40<br>5 = > 40
8	Daily Treatment and Airways Review - Day 1 Onwa		intensive_therapy_id	Intensive therapy ID		linked_form			
8	Daily Treatment and Airways Review - D	1.1	naxdate	Assessment Date		date			
8	Daily Treatment and Airways Review - D	1.2	naxintrtyn	Was the participant still receiving intensive therapy (IV antibiotics) today (between 00:00-23:59)?		boolean			1 = Yes<br>0 = No
8	Daily Treatment and Airways Review - D	1.2.1	naxlasdosdat	Date of last dose of IV antibiotic treatment		date			
8	Daily Treatment and Airways Review - D	1.3	naxdischyn	Was the participant discharged today (between 00:00-23:59)?		boolean			1 = Yes<br>0 = No
8	Daily Treatment and Airways Review - D	1.3.1	naxloc	Location of admission on this day		option			1 = Hospital<br>2 = HITH
8	Daily Treatment and Airways Review - D	1.3.1.1	naxhosp	Hospital		option			1 = Current hospital<br>2 = Regional hospital
8	Daily Treatment and Airways Review - D	1.3.2	naxdischdat	Date of discharge		date			
8	Daily Treatment and Airways Review - D	2.1	caxcoughtp	Describe the type of cough on request		option			1 = Wet<br>2 = Dry
8	Daily Treatment and Airways Review - D	2.1a	caxcoughtpna	Not done		checkbox		0	1 = Yes<br>0 = No
8	Daily Treatment and Airways Review - D	2.1.1	caxcoughbest	Is the cough back to how dry it usually is when this patient is well?		option			1 = Yes<br>0 = No<br>-1 = Unknown
8	Daily Treatment and Airways Review - D	2.2	caxcrepit	Crepitations		boolean			1 = Yes<br>0 = No
8	Daily Treatment and Airways Review - D	2.2a	caxcrepitna	Not done		checkbox		0	1 = Yes<br>0 = No
8	Daily Treatment and Airways Review - D	3.1	naxabxbckchg	Backbone antibiotic changed?		boolean			1 = Yes<br>0 = No
8	Daily Treatment and Airways Review - D	3.2	naxabxadjchg	Adjunct antibiotic changed?		boolean			1 = Yes<br>0 = No
8	Daily Treatment and Airways Review - D	3.3	naxabxaddchg	Any changes to additional antibiotics?		boolean			1 = Yes<br>0 = No

8	Daily Treatment and Airways Review - D	3.3.1	naxabxaddchgrsn	Reason for change to additional antibiotic		option			1 = Completed<br>2 = Unsatisfactory response<br>3 = Adverse reaction<br>4 = New micro results<br>5 = Not available<br>7 = Dose adjustment<br>6 = Other
8	Daily Treatment and Airways Review - D	4.1	naxithchg	Any changes to inhaled therapy treatment?		boolean			1 = Yes<br>0 = No
8	Daily Treatment and Airways Review - D	4.2	naxainchg	Any changes to anti-inflammatory treatment?		boolean			1 = Yes<br>0 = No
8	Daily Treatment and Airways Review - D	5.1	naxconchg	Any new or changes to any current concomitant medications?		boolean			1 = Yes<br>0 = No
8	Daily Treatment and Airways Review - D	6.1	naxmicro	Were any new airway samples for microbiology taken?		boolean			1 = Yes<br>0 = No
9	Physiotherapy Outcomes - Completed at end of Ir		intensive_therapy_id	Intensive therapy ID		linked_form			
9	Physiotherapy Outcomes - Completed a	1.1	phoutdat	Date of completion of this form		date			
9	Physiotherapy Outcomes - Completed a	1.2	phout6min	Was 6 Minute Walk Test Performed?		boolean			1 = Yes<br>0 = No
9	Physiotherapy Outcomes - Completed a	1.2.1	phout6minres	Result		integer	m		
9	Physiotherapy Outcomes - Completed a	1.2.2	phout6mindat	Date		date			
9	Physiotherapy Outcomes - Completed a	1.3	phoutmodsh	Was Modified Shuttle Test Performed?		boolean			1 = Yes<br>0 = No
9	Physiotherapy Outcomes - Completed a	1.3.1	phoutmodshres	Result		decimal	level		
9	Physiotherapy Outcomes - Completed a	1.3.2	phoutmodshdat	Date		date			
9	Physiotherapy Outcomes - Completed a	1.3.3	phoutmodshtyp	Type		option			1 = 10 metres<br>2 = 20 metres
9	Physiotherapy Outcomes - Completed a	1.4	phoutstep	Was Step Test Performed?		boolean			1 = Yes<br>0 = No
9	Physiotherapy Outcomes - Completed a	1.4.1	phoutstepres	Result		integer	min		
9	Physiotherapy Outcomes - Completed a	1.4.2	phoutstepdat	Date		date			
9	Physiotherapy Outcomes - Completed a	1.5	phoutcpet	Was CPET Test Performed?		boolean			1 = Yes<br>0 = No
9	Physiotherapy Outcomes - Completed a	1.5.1	phoutcpetres	Result		integer	VO2 peak % predicted		
9	Physiotherapy Outcomes - Completed a	1.5.2	phoutcpetdat	Date		date			
9	Physiotherapy Outcomes - Completed a	1.6	phoutoth	Any other tests performed?		boolean			1 = Yes<br>0 = No
9	Physiotherapy Outcomes - Completed a	1.6.1.1	phoutoth1spec	Specify		string			
9	Physiotherapy Outcomes - Completed a	1.6.1.2	phoutoth1res	Result		decimal			
9	Physiotherapy Outcomes - Completed a	1.6.1.3	phoutoth1dat	Date		date			
10	Spirometry Log	1.1	fev1datetime	Date/time of measure		datetime			
10	Spirometry Log	1.2	fev1result	FEV1 result		decimal	litres		
10	Spirometry Log	1.3	fev1height	Height		decimal	cm		
10	Spirometry Log	1.4	fev1location	Location		option			1 = Patient's home<br>2 = Clinic/hospital<br>9 = Unknown
10	Spirometry Log	1.5	fev1operator	Performed by		option			1 = Patient with their own equipment<br>2 = Clinician/technician with their own equipment<br>9 = Unknown
10	Spirometry Log	1.6	fev1concerns	Technical concerns		checkbox		0	1 = Yes<br>0 = No
11	Faecal Clostridium Difficile Testing	1.1	cddat	Faecal Sample Date		date			
11	Faecal Clostridium Difficile Testing	1.2	cdres	Clostridium Difficile (toxin) result		option			1 = Positive<br>2 = Negative<br>3 = Equivocal
11	Faecal Clostridium Difficile Testing	1.2.1	cdsymp	Is the participant symptomatic (i.e. looser than normal stools)?		boolean			1 = Yes<br>0 = No
11	Faecal Clostridium Difficile Testing	1.2.2	cdprevpos	Has there been a previous POSITIVE Clostridium difficile result in the preceding 2 years?		boolean			1 = Yes<br>0 = No
11	Faecal Clostridium Difficile Testing	1.2.2.1	cdprevposdat	Sample date for the previous POSITIVE faecal sample		date			
12	Antibiotic Reaction Events	1.1	abrabx	Name of Antibiotic		option			1 = Amoxicillin<br>2 = Amoxicillin clavulanate<br>4 = Amikacin<br>5 = Amphotericin B<br>6 = Ampicillin<br>7 = Azithromycin<br>8 = Aztreonam<br>9 = Caspofungin<br>10 = Cefaclor<br>11 = Cefazolin<br>12 = Cefepime<br>13 = Cefotaxime<br>14 = Ceftriaxone<br>15 = Ceftazidime<br>59 = Ceftazidime/avibactam<br>16 = Cephalexin<br>17 = Cephalothin<br>18 = Chloramphenicol<br>19 = Ciprofloxacin<br>20 = Clarithromycin<br>21 = Clindamycin<br>22 = Colistin<br>23 = Cotrimoxazole (Trimethoprim and sulfamethoxa<br>24 = Daptomycin<br>25 = Dicloxacillin<br>26 = Doxycycline<br>27 = Ertapenem<br>28 = Erythromycin<br>29 = Flucloxacillin<br>30 = Fluconazole<br>31 = Fosfomycin<br>32 = Fusidic acid<br>33 = Gentamicin<br>34 = Imipenem<br>35 = Itraconazole<br>36 = Ketoconazole<br>37 = Linezolid<br>38 = Meropenem<br>58 = Methicillin<br>39 = Metronidazole<br>40 = Minocycline<br>41 = Moxifloxacin<br>42 = Mupirocin<br>43 = Nitrofurantoin<br>44 = Norfloxacin<br>45 = Ofloxacin<br>46 = Penicillin/benzylpenicillin<br>47 = Piperacillin tazobactam (Tazocin)<br>48 = Posaconazole<br>49 = Rifampicin<br>50 = Teicoplanin<br>51 = Tetracycline<br>52 = Ticarcillin clavulanate (Timentin)<br>53 = Tigecycline<br>54 = Tobramycin<br>55 = Trimethoprim<br>56 = Vancomycin<br>57 = Voriconazole<br>999 = Other
12	Antibiotic Reaction Events	1.1a	abrabxoth	Other, Please specify		string			
12	Antibiotic Reaction Events	1.2	abrecall	Patient recall of potential reaction		option			1 = Patient can describe reaction<br>2 = Patient cannot describe reaction
12	Antibiotic Reaction Events	1.3	abrstdat	Start date of antibiotic		date			
12	Antibiotic Reaction Events	1.3.a	abrstdatunk	Unknown		checkbox		0	1 = Yes<br>0 = No
12	Antibiotic Reaction Events	1.5	abrroute	Route of administration		option			1 = Inhalation<br>2 = Intramuscular<br>3 = Intravenous<br>4 = Oral (or gastrostomy)<br>5 = Subcutaneous<br>6 = Topical<br>7 = Other<br>99 = Not known
12	Antibiotic Reaction Events	2.1a	abrprssick	felt sick		checkbox		0	1 = Yes<br>0 = No
12	Antibiotic Reaction Events	2.1b	abrprsitchtong	itchy tongue		checkbox		0	1 = Yes<br>0 = No
12	Antibiotic Reaction Events	2.1c	abrprsitcheyes	itchy eyes		checkbox		0	1 = Yes<br>0 = No
12	Antibiotic Reaction Events	2.1d	abrprsitchthrt	itchy throat		checkbox		0	1 = Yes<br>0 = No
12	Antibiotic Reaction Events	2.1e	abrprsitchall	itching all over		checkbox		0	1 = Yes<br>0 = No
12	Antibiotic Reaction Events	2.1f	abrprsthrtsores	sores in throat		checkbox		0	1 = Yes<br>0 = No
12	Antibiotic Reaction Events	2.1g	abrprssnz	sneezing		checkbox		0	1 = Yes<br>0 = No
12	Antibiotic Reaction Events	2.1h	abrprscough	cough		checkbox		0	1 = Yes<br>0 = No
12	Antibiotic Reaction Events	2.1i	abrprsbreath	short of breath		checkbox		0	1 = Yes<br>0 = No
12	Antibiotic Reaction Events	2.1j	abrprseyesw	swelling around eyes		checkbox		0	1 = Yes<br>0 = No
12	Antibiotic Reaction Events	2.1k	abrprsconf	confused		checkbox		0	1 = Yes<br>0 = No
12	Antibiotic Reaction Events	2.1m	abrprshead	headache		checkbox		0	1 = Yes<br>0 = No
12	Antibiotic Reaction Events	2.1n	abrprsdizz	dizziness		checkbox		0	1 = Yes<br>0 = No
12	Antibiotic Reaction Events	2.1p	abrprsmsclpn	muscle pain		checkbox		0	1 = Yes<br>0 = No
12	Antibiotic Reaction Events	2.1q	abrprsafrd	afraid		checkbox		0	1 = Yes<br>0 = No
12	Antibiotic Reaction Events	2.1r	abrprssweat	sweating		checkbox		0	1 = Yes<br>0 = No
12	Antibiotic Reaction Events	2.1t	abrprsfaint	faint		checkbox		0	1 = Yes<br>0 = No
12	Antibiotic Reaction Events	2.1u	abrprsoth	other		checkbox		0	1 = Yes<br>0 = No
12	Antibiotic Reaction Events	2.2	abrprsothspec	Other symptoms described by the patient		text			
12	Antibiotic Reaction Events	3.1a	abrrashnon	Non-specific (eg macular/maculopapular)		checkbox		0	1 = Yes<br>0 = No
12	Antibiotic Reaction Events	3.1b	abrrashurti	Urticaria		checkbox		0	1 = Yes<br>0 = No
12	Antibiotic Reaction Events	3.1c	abrrashblist	Skin blistering		checkbox		0	1 = Yes<br>0 = No
12	Antibiotic Reaction Events	3.1d	abrrashmucos	Mucosal involvement		checkbox		0	1 = Yes<br>0 = No

12	Antibiotic Reaction Events	3.1e	abrrasheryth	Erythema		checkbox		0	1 = Yes<br>0 = No
12	Antibiotic Reaction Events	3.1f	abrrashitch	Itch (without rash)		checkbox		0	1 = Yes<br>0 = No
12	Antibiotic Reaction Events	3.1g	abrrashnone	None		checkbox		0	1 = Yes<br>0 = No
12	Antibiotic Reaction Events	3.2a	abredemaperiorb	Peri-orbital		checkbox		0	1 = Yes<br>0 = No
12	Antibiotic Reaction Events	3.2b	abredemafacial	Facial		checkbox		0	1 = Yes<br>0 = No
12	Antibiotic Reaction Events	3.2c	abredemalip	Lip		checkbox		0	1 = Yes<br>0 = No
12	Antibiotic Reaction Events	3.2d	abredemaperi	Peripheral		checkbox		0	1 = Yes<br>0 = No
12	Antibiotic Reaction Events	3.2e	abredemanosp	Site not specified		checkbox		0	1 = Yes<br>0 = No
12	Antibiotic Reaction Events	3.2f	abredemanone	None		checkbox		0	1 = Yes<br>0 = No
12	Antibiotic Reaction Events	3.3a	abrrespupstri	Stridor, hoarse/loss of voice		checkbox		0	1 = Yes<br>0 = No
12	Antibiotic Reaction Events	3.3b	abrrespupthrt	Tight throat		checkbox		0	1 = Yes<br>0 = No
12	Antibiotic Reaction Events	3.3c	abrrespuptong	Tongue swelling		checkbox		0	1 = Yes<br>0 = No
12	Antibiotic Reaction Events	3.3d	abrrespupdrool	Drooling		checkbox		0	1 = Yes<br>0 = No
12	Antibiotic Reaction Events	3.3e	abrrespupnone	None		checkbox		0	1 = Yes<br>0 = No
12	Antibiotic Reaction Events	3.4a	abrresplowhze	Wheeze		checkbox		0	1 = Yes<br>0 = No
12	Antibiotic Reaction Events	3.4b	abrresplocough	Persistent cough		checkbox		0	1 = Yes<br>0 = No
12	Antibiotic Reaction Events	3.4c	abrresplohyp	Hypoxia		checkbox		0	1 = Yes<br>0 = No
12	Antibiotic Reaction Events	3.4d	abrresplonone	None		checkbox		0	1 = Yes<br>0 = No
12	Antibiotic Reaction Events	3.5a	abrcardiotach	Tachycardia		checkbox		0	1 = Yes<br>0 = No
12	Antibiotic Reaction Events	3.5b	abrcardiohypo	Hypotension		checkbox		0	1 = Yes<br>0 = No
12	Antibiotic Reaction Events	3.5c	abrcardiopale	Pale/floppy		checkbox		0	1 = Yes<br>0 = No
12	Antibiotic Reaction Events	3.5d	abrcardioconsc	Loss of consciousness		checkbox		0	1 = Yes<br>0 = No
12	Antibiotic Reaction Events	3.5e	abrcardionone	None		checkbox		0	1 = Yes<br>0 = No
12	Antibiotic Reaction Events	3.6a	abrgastrodia	Diarrhoea		checkbox		0	1 = Yes<br>0 = No
12	Antibiotic Reaction Events	3.6b	abrgastrovom	Vomiting		checkbox		0	1 = Yes<br>0 = No
12	Antibiotic Reaction Events	3.6c	abrgastroabdo	Abdominal pain		checkbox		0	1 = Yes<br>0 = No
12	Antibiotic Reaction Events	3.6d	abrgastronone	None		checkbox		0	1 = Yes<br>0 = No
12	Antibiotic Reaction Events	3.7a	abrsysfever	Fever		checkbox		0	1 = Yes<br>0 = No
12	Antibiotic Reaction Events	3.7b	abrsysnone	None		checkbox		0	1 = Yes<br>0 = No
12	Antibiotic Reaction Events	3.8	abrsymoth	Any additional information regarding reaction		text			
12	Antibiotic Reaction Events	4.1	abrint	Interval between last dose and suspected reaction		option			1 = < 1 hour<br>2 = 1-6 hours<br>3 = 6-24 hours<br>4 = 24-48 hours<br>5 = > 48 hours<br>9 = Unknown
12	Antibiotic Reaction Events	4.2	abrday	On which day of the course did the reaction occur?		option			1 = Day 1<br>2 = Day 2<br>3 = Day 3-5<br>4 = Day 6-7<br>5 = Beyond Day 7<br>6 = Post completion<br>9 = Unknown
12	Antibiotic Reaction Events	4.3	abrdose	After which number dose since the commencement of this course of antibiotics did the reaction occur?		integer			
12	Antibiotic Reaction Events	4.3a	abrdoseunk	Unknown		checkbox		0	1 = Yes<br>0 = No
12	Antibiotic Reaction Events	4.4	abrprevex	Any previous exposure to this antibiotic?		option			1 = Yes<br>0 = No<br>-1 = Unknown
12	Antibiotic Reaction Events	4.5	abrprevclas	Any previous exposure to the same class of antibiotic?		option			1 = Yes<br>0 = No<br>-1 = Unknown
12	Antibiotic Reaction Events	4.5.1	abrprevclaspec	Specify antibiotic		string			
12	Antibiotic Reaction Events	4.6	abrceas	Was the antibiotic ceased as a result of this reaction?		option			1 = Yes<br>0 = No<br>-1 = Unknown
12	Antibiotic Reaction Events	4.7	abrsympers	For how long were the symptoms persisting?		option			1 = Hours<br>2 = Days<br>3 = Unknown
12	Antibiotic Reaction Events	4.7.1	abrpershrspec	Specify the number of hours		decimal	hours		
12	Antibiotic Reaction Events	4.7.2	abrpersdyspec	Specify the number of days		integer	days		
12	Antibiotic Reaction Events	4.8	abrtrt	Was any treatment given for the symptoms?		option			1 = Yes<br>0 = No<br>-1 = Unknown
12	Antibiotic Reaction Events	4.8.1a	abrtrtahiv	Antihistamines IV		checkbox		0	1 = Yes<br>0 = No
12	Antibiotic Reaction Events	4.8.1b	abrtrtahpo	Antihistamines PO		checkbox		0	1 = Yes<br>0 = No
12	Antibiotic Reaction Events	4.8.1c	abrtrtcsiv	Corticosteroids IV		checkbox		0	1 = Yes<br>0 = No
12	Antibiotic Reaction Events	4.8.1d	abrtrtcspo	Corticosteroids PO		checkbox		0	1 = Yes<br>0 = No
12	Antibiotic Reaction Events	4.8.1e	abrtrtcsto	Corticosteroids topically		checkbox		0	1 = Yes<br>0 = No
12	Antibiotic Reaction Events	4.8.1f	abrtrtadiv	Adrenaline IV		checkbox		0	1 = Yes<br>0 = No
12	Antibiotic Reaction Events	4.8.1g	abrtrtadim	Adrenaline IM (e.g. EpiPen)		checkbox		0	1 = Yes<br>0 = No
12	Antibiotic Reaction Events	4.8.1h	abrtrtsi	Salbutamol inhalation (e.g. ventolin)		checkbox		0	1 = Yes<br>0 = No
12	Antibiotic Reaction Events	4.8.1i	abrtrtoth	Other treatment		checkbox		0	1 = Yes<br>0 = No
12	Antibiotic Reaction Events	4.8.1i.1	abrtrtothspec	Specify		string			
12	Antibiotic Reaction Events	4.9	abrmed	Was medical attention sought for the reaction?		boolean			1 = Yes<br>0 = No
12	Antibiotic Reaction Events	4.9.1a	abrmedgp	GP		checkbox		0	1 = Yes<br>0 = No
12	Antibiotic Reaction Events	4.9.1b	abrmedhd	Health Direct		checkbox		0	1 = Yes<br>0 = No
12	Antibiotic Reaction Events	4.9.1c	abrmeded	ED		checkbox		0	1 = Yes<br>0 = No
12	Antibiotic Reaction Events	4.9.1d	abrmedinp	Inpatient at the time		checkbox		0	1 = Yes<br>0 = No
12	Antibiotic Reaction Events	4.9.1e	abrmedcp	Community pharmacy		checkbox		0	1 = Yes<br>0 = No
12	Antibiotic Reaction Events	4.9.1f	abrmedoth	Other		checkbox		0	1 = Yes<br>0 = No
12	Antibiotic Reaction Events	4.9.1g	abrmedothspec	Specify other type of medical attention		string			
12	Antibiotic Reaction Events	4.10	abrsymnodrug	Have similar symptoms been observed without intake of suspicious drug(s)?		option			1 = Yes<br>0 = No<br>-1 = Unknown
12	Antibiotic Reaction Events	4.10.1	abrsymcause	Please specify		string			
12	Antibiotic Reaction Events	4.11	abrothdrug	Was the patient taking any other drugs at the time the reaction occurred?		option			1 = Yes<br>0 = No<br>-1 = Unknown
12	Antibiotic Reaction Events	4.12	abrothab	Does this reaction create a contraindication to any of the following antibiotics?		boolean			1 = Yes<br>0 = No
12	Antibiotic Reaction Events	5.2	abrwcc	White cell count		decimal	x109/L		
12	Antibiotic Reaction Events	5.2a	abrwccna	N/A		checkbox		0	1 = Yes<br>0 = No
12	Antibiotic Reaction Events	5.3	abreosinophils	Eosinophils count		decimal	x109/L		
12	Antibiotic Reaction Events	5.3a	abreosinophilsna	N/A		checkbox		0	1 = Yes<br>0 = No
12	Antibiotic Reaction Events	5.4	abrneutrophils	Neutrophils count		decimal	x109/L		
12	Antibiotic Reaction Events	5.4a	abrneutrophilsna	N/A		checkbox		0	1 = Yes<br>0 = No
12	Antibiotic Reaction Events	5.5	abrhgb	Haemoglobin		decimal	g/dL		
12	Antibiotic Reaction Events	5.5a	abrhgbna	N/A		checkbox		0	1 = Yes<br>0 = No
12	Antibiotic Reaction Events	5.6	abrplt	Platelets		decimal	x109/L		
12	Antibiotic Reaction Events	5.6a	abrpltna	N/A		checkbox		0	1 = Yes<br>0 = No
12	Antibiotic Reaction Events	5.7	abralt	ALT		decimal	U/L		
12	Antibiotic Reaction Events	5.7a	abraltna	N/A		checkbox		0	1 = Yes<br>0 = No
12	Antibiotic Reaction Events	5.8	abrast	AST		decimal	U/L		
12	Antibiotic Reaction Events	5.8a	abrastna	N/A		checkbox		0	1 = Yes<br>0 = No
12	Antibiotic Reaction Events	5.9	abrtbr	Total bilirubin		decimal	µmol/L		
12	Antibiotic Reaction Events	5.9a	abrtbrna	N/A		checkbox		0	1 = Yes<br>0 = No
12	Antibiotic Reaction Events	5.10	abrurea	Urea		decimal	mmol/L		
12	Antibiotic Reaction Events	5.10a	abrureana	N/A		checkbox		0	1 = Yes<br>0 = No
12	Antibiotic Reaction Events	5.11	abrcreat	Creatinine		decimal	µmol/L		
12	Antibiotic Reaction Events	5.11a	abrcreatna	N/A		checkbox		0	1 = Yes<br>0 = No
12	Antibiotic Reaction Events	5.12	abrcrp	CRP		decimal	mg/L		
12	Antibiotic Reaction Events	5.12a	abrcrpna	N/A		checkbox		0	1 = Yes<br>0 = No
12	Antibiotic Reaction Events	5.13	abrtryp	Tryptase		decimal	U/mL		
12	Antibiotic Reaction Events	5.13a	abrtrypna	N/A		checkbox		0	1 = Yes<br>0 = No

**13**	Co-enrolment in Other Studies	1.1	coenrolment_name	Name of other study		string			
**13**	Co-enrolment in Other Studies	1.2	coenrolment_number	Participant number (ID)		string			
**13**	Co-enrolment in Other Studies	1.3	coenrolment_drug	Study involve taking specified medications?		boolean			1 = Yes<br>0 = No
**13**	Co-enrolment in Other Studies	1.4	coenrolment_start_date	Date of enrolment		date			
**13**	Co-enrolment in Other Studies	1.5	coenrolment_end_date	Date of completion		date			
**14.1**	CFQR - Parent/Caregiver		cfqr_parent_carer_datetime	Completion date/time		datetime			
**14.1**	CFQR - Parent/Caregiver	1	cfqr_parent_carer_q1	Performing vigorous activities such as running or playing sports		option			1 = A lot of difficulty<br>2 = Some difficulty<br>3 = A little difficulty<br>4 = No difficulty
**14.1**	CFQR - Parent/Caregiver	2	cfqr_parent_carer_q2	Walking as fast as others		option			1 = A lot of difficulty<br>2 = Some difficulty<br>3 = A little difficulty<br>4 = No difficulty
**14.1**	CFQR - Parent/Caregiver	3	cfqr_parent_carer_q3	Climbing stairs as fast as others		option			1 = A lot of difficulty<br>2 = Some difficulty<br>3 = A little difficulty<br>4 = No difficulty
**14.1**	CFQR - Parent/Caregiver	4	cfqr_parent_carer_q4	Carrying or lifting heavy objects such as books, school bag, or backpack		option			1 = A lot of difficulty<br>2 = Some difficulty<br>3 = A little difficulty<br>4 = No difficulty
**14.1**	CFQR - Parent/Caregiver	5	cfqr_parent_carer_q5	Climbing several flights of stairs		option			1 = A lot of difficulty<br>2 = Some difficulty<br>3 = A little difficulty<br>4 = No difficulty
**14.1**	CFQR - Parent/Caregiver	6	cfqr_parent_carer_q6	Seemed happy		option			4 = Always<br>3 = Often<br>2 = Sometimes<br>1 = Never
**14.1**	CFQR - Parent/Caregiver	7	cfqr_parent_carer_q7	Seemed worried		option			1 = Always<br>2 = Often<br>3 = Sometimes<br>4 = Never
**14.1**	CFQR - Parent/Caregiver	8	cfqr_parent_carer_q8	Seemed tired		option			1 = Always<br>2 = Often<br>3 = Sometimes<br>4 = Never
**14.1**	CFQR - Parent/Caregiver	9	cfqr_parent_carer_q9	Seemed short-tempered		option			1 = Always<br>2 = Often<br>3 = Sometimes<br>4 = Never
**14.1**	CFQR - Parent/Caregiver	10	cfqr_parent_carer_q10	Seemed well		option			4 = Always<br>3 = Often<br>2 = Sometimes<br>1 = Never
**14.1**	CFQR - Parent/Caregiver	11	cfqr_parent_carer_q11	Seemed grouchy		option			1 = Always<br>2 = Often<br>3 = Sometimes<br>4 = Never
**14.1**	CFQR - Parent/Caregiver	12	cfqr_parent_carer_q12	Seemed full of energy		option			4 = Always<br>3 = Often<br>2 = Sometimes<br>1 = Never
**14.1**	CFQR - Parent/Caregiver	13	cfqr_parent_carer_q13	Was absent or late for school or other activites because of his/her illness or treatments		option			1 = Always<br>2 = Often<br>3 = Sometimes<br>4 = Never
**14.1**	CFQR - Parent/Caregiver	14	cfqr_parent_carer_q14	The extent to which your child participated in sports and other physical activities, such as P.E. (physical education)		option			1 = Has not participated in physical activities<br>2 = Has participated less than usual in sports<br>3 = Has participated as much as usual but with some<br>4 = Has been able to participate in physical activities
**14.1**	CFQR - Parent/Caregiver	15	cfqr_parent_carer_q15	The extent to which your child has difficulty walking		option			4 = He or she can walk a long time without getting tir<br>3 = He or she can walk a long time but gets tired<br>2 = He or she cannot walk a long time, because he o<br>1 = He or she avoids walking whenever possible, bec
**14.1**	CFQR - Parent/Caregiver	16	cfqr_parent_carer_q16	My child has trouble recovering after physical effort		option			1 = Very true<br>2 = Somewhat true<br>3 = Somewhat false<br>4 = Very false
**14.1**	CFQR - Parent/Caregiver	17	cfqr_parent_carer_q17	Mealtimes are a struggle		option			1 = Very true<br>2 = Somewhat true<br>3 = Somewhat false<br>4 = Very false
**14.1**	CFQR - Parent/Caregiver	18	cfqr_parent_carer_q18	My child's treatments get in the way of his/her activities		option			1 = Very true<br>2 = Somewhat true<br>3 = Somewhat false<br>4 = Very false
**14.1**	CFQR - Parent/Caregiver	19	cfqr_parent_carer_q19	My child feels small compared to other kids the same age		option			1 = Very true<br>2 = Somewhat true<br>3 = Somewhat false<br>4 = Very false
**14.1**	CFQR - Parent/Caregiver	20	cfqr_parent_carer_q20	My child feels physically different from other kids the same age		option			1 = Very true<br>2 = Somewhat true<br>3 = Somewhat false<br>4 = Very false
**14.1**	CFQR - Parent/Caregiver	21	cfqr_parent_carer_q21	My child thinks that he/she is too thin		option			1 = Very true<br>2 = Somewhat true<br>3 = Somewhat false<br>4 = Very false
**14.1**	CFQR - Parent/Caregiver	22	cfqr_parent_carer_q22	My child feels healthy		option			4 = Very true<br>3 = Somewhat true<br>2 = Somewhat false<br>1 = Very false
**14.1**	CFQR - Parent/Caregiver	23	cfqr_parent_carer_q23	My child tends to be withdrawn		option			1 = Very true<br>2 = Somewhat true<br>3 = Somewhat false<br>4 = Very false
**14.1**	CFQR - Parent/Caregiver	24	cfqr_parent_carer_q24	My child leads a normal life		option			4 = Very true<br>3 = Somewhat true<br>2 = Somewhat false<br>1 = Very false
**14.1**	CFQR - Parent/Caregiver	25	cfqr_parent_carer_q25	My child has less fun than usual		option			1 = Very true<br>2 = Somewhat true<br>3 = Somewhat false<br>4 = Very false
**14.1**	CFQR - Parent/Caregiver	26	cfqr_parent_carer_q26	My child has trouble getting along with others		option			1 = Very true<br>2 = Somewhat true<br>3 = Somewhat false<br>4 = Very false
**14.1**	CFQR - Parent/Caregiver	27	cfqr_parent_carer_q27	My child has trouble concentrating		option			1 = Very true<br>2 = Somewhat true<br>3 = Somewhat false<br>4 = Very false
**14.1**	CFQR - Parent/Caregiver	28	cfqr_parent_carer_q28	My child is able to keep up with his/her school work or holiday activities		option			4 = Very true<br>3 = Somewhat true<br>2 = Somewhat false<br>1 = Very false
**14.1**	CFQR - Parent/Caregiver	29	cfqr_parent_carer_q29	My child is not doing as well as usual in school or holiday activities		option			1 = Very true<br>2 = Somewhat true<br>3 = Somewhat false<br>4 = Very false
**14.1**	CFQR - Parent/Caregiver	30	cfqr_parent_carer_q30	My child spends a lot of time on his/her treatments everyday		option			1 = Very true<br>2 = Somewhat true<br>3 = Somewhat false<br>4 = Very false
**14.1**	CFQR - Parent/Caregiver	31	cfqr_parent_carer_q31	How difficult is it for your child to do his/her treatments (including medications) each day?		option			4 = Not at all<br>3 = A little<br>2 = Moderately<br>1 = Very
**14.1**	CFQR - Parent/Caregiver	32	cfqr_parent_carer_q32	How do you think your child's health is now?		option			4 = Excellent<br>3 = Good<br>2 = Fair<br>1 = Poor
**14.1**	CFQR - Parent/Caregiver	33	cfqr_parent_carer_q33	My child had trouble gaining weight		option			1 = A great deal<br>2 = Somewhat<br>3 = A little<br>4 = Not at all
**14.1**	CFQR - Parent/Caregiver	34	cfqr_parent_carer_q34	My child was congested		option			1 = A great deal<br>2 = Somewhat<br>3 = A little<br>4 = Not at all
**14.1**	CFQR - Parent/Caregiver	35	cfqr_parent_carer_q35	My child coughed during the day		option			1 = A great deal<br>2 = Somewhat<br>3 = A little<br>4 = Not at all
**14.1**	CFQR - Parent/Caregiver	36	cfqr_parent_carer_q36	My child had to cough up mucus		option			1 = A great deal<br>2 = Somewhat<br>3 = A little<br>4 = Not at all
**14.1**	CFQR - Parent/Caregiver	37	cfqr_parent_carer_q37	My child's mucus has been mostly:		option			1 = Clear<br>2 = Clear to yellow<br>3 = Yellowish-green<br>4 = Green with traces of blood<br>5 = Don't know
**14.1**	CFQR - Parent/Caregiver	38	cfqr_parent_carer_q38	My child wheezed		option			1 = Always<br>2 = Often<br>3 = Sometimes<br>4 = Never
**14.1**	CFQR - Parent/Caregiver	39	cfqr_parent_carer_q39	My child had trouble breathing		option			1 = Always<br>2 = Often<br>3 = Sometimes<br>4 = Never
**14.1**	CFQR - Parent/Caregiver	40	cfqr_parent_carer_q40	My child woke up during the night because he/she was coughing		option			1 = Always<br>2 = Often<br>3 = Sometimes<br>4 = Never
**14.1**	CFQR - Parent/Caregiver	41	cfqr_parent_carer_q41	My child had wind		option			1 = Always<br>2 = Often<br>3 = Sometimes<br>4 = Never
**14.1**	CFQR - Parent/Caregiver	42	cfqr_parent_carer_q42	My child had diarrhea		option			1 = Always<br>2 = Often<br>3 = Sometimes<br>4 = Never
**14.1**	CFQR - Parent/Caregiver	43	cfqr_parent_carer_q43	My child had abdominal pain		option			1 = Always<br>2 = Often<br>3 = Sometimes<br>4 = Never
**14.1**	CFQR - Parent/Caregiver	44	cfqr_parent_carer_q44	My child has had eating problems		option			1 = Always<br>2 = Often<br>3 = Sometimes<br>4 = Never
**14.2**	CFQR - Ages 12 and 13		cfqr_ages_12_13_datetime	Completion date/time		datetime			
**14.2**	CFQR - Ages 12 and 13	1	cfqr_ages_12_13_q1	You were able to walk as fast as others		option			4 = Very True<br>3 = Mostly True<br>2 = Somewhat True<br>1 = Not at all True
**14.2**	CFQR - Ages 12 and 13	2	cfqr_ages_12_13_q2	You were able to climb stairs as fast as others		option			4 = Very True<br>3 = Mostly True<br>2 = Somewhat True<br>1 = Not at all True

**14.2**	CFQR - Ages 12 and 13	3	cfqr_ages_12_13_q3	You were able to run, jump, and climb as you wanted		option			4 = Very True<br>3 = Mostly True<br>2 = Somewhat True<br>1 = Not at all True
**14.2**	CFQR - Ages 12 and 13	4	cfqr_ages_12_13_q4	You were able to run as quickly and for as long as others		option			4 = Very True<br>3 = Mostly True<br>2 = Somewhat True<br>1 = Not at all True
**14.2**	CFQR - Ages 12 and 13	5	cfqr_ages_12_13_q5	You were able to participate in sports that you enjoy (e.g., swimming, football, dancing or others)		option			4 = Very True<br>3 = Mostly True<br>2 = Somewhat True<br>1 = Not at all True
**14.2**	CFQR - Ages 12 and 13	6	cfqr_ages_12_13_q6	You had difficulty carrying or lifting heavy things such as books, your school bag, or a backpack		option			1 = Very True<br>2 = Mostly True<br>3 = Somewhat True<br>4 = Not at all True
**14.2**	CFQR - Ages 12 and 13	7	cfqr_ages_12_13_q7	You felt tired		option			1 = Always<br>2 = Often<br>3 = Sometimes<br>4 = Never
**14.2**	CFQR - Ages 12 and 13	8	cfqr_ages_12_13_q8	You felt mad		option			1 = Always<br>2 = Often<br>3 = Sometimes<br>4 = Never
**14.2**	CFQR - Ages 12 and 13	9	cfqr_ages_12_13_q9	You felt grouchy		option			1 = Always<br>2 = Often<br>3 = Sometimes<br>4 = Never
**14.2**	CFQR - Ages 12 and 13	10	cfqr_ages_12_13_q10	You felt worried		option			1 = Always<br>2 = Often<br>3 = Sometimes<br>4 = Never
**14.2**	CFQR - Ages 12 and 13	11	cfqr_ages_12_13_q11	You felt sad		option			1 = Always<br>2 = Often<br>3 = Sometimes<br>4 = Never
**14.2**	CFQR - Ages 12 and 13	12	cfqr_ages_12_13_q12	You had trouble falling asleep		option			1 = Always<br>2 = Often<br>3 = Sometimes<br>4 = Never
**14.2**	CFQR - Ages 12 and 13	13	cfqr_ages_12_13_q13	You had bad dreams or nightmares		option			1 = Always<br>2 = Often<br>3 = Sometimes<br>4 = Never
**14.2**	CFQR - Ages 12 and 13	14	cfqr_ages_12_13_q14	You felt good about yourself		option			4 = Always<br>3 = Often<br>2 = Sometimes<br>1 = Never
**14.2**	CFQR - Ages 12 and 13	15	cfqr_ages_12_13_q15	You had trouble eating		option			1 = Always<br>2 = Often<br>3 = Sometimes<br>4 = Never
**14.2**	CFQR - Ages 12 and 13	16	cfqr_ages_12_13_q16	You had to stop fun activities to do your treatments		option			1 = Always<br>2 = Often<br>3 = Sometimes<br>4 = Never
**14.2**	CFQR - Ages 12 and 13	17	cfqr_ages_12_13_q17	You were forced to eat		option			1 = Always<br>2 = Often<br>3 = Sometimes<br>4 = Never
**14.2**	CFQR - Ages 12 and 13	18	cfqr_ages_12_13_q18	You were able to do all of your treatments		option			4 = Very True<br>3 = Mostly True<br>2 = Somewhat True<br>1 = Not at all True
**14.2**	CFQR - Ages 12 and 13	19	cfqr_ages_12_13_q19	You enjoyed eating		option			4 = Very True<br>3 = Mostly True<br>2 = Somewhat True<br>1 = Not at all True
**14.2**	CFQR - Ages 12 and 13	20	cfqr_ages_12_13_q20	You got together with friends a lot		option			4 = Very True<br>3 = Mostly True<br>2 = Somewhat True<br>1 = Not at all True
**14.2**	CFQR - Ages 12 and 13	21	cfqr_ages_12_13_q21	You stayed at home more often than you wanted to		option			1 = Very True<br>2 = Mostly True<br>3 = Somewhat True<br>4 = Not at all True
**14.2**	CFQR - Ages 12 and 13	22	cfqr_ages_12_13_q22	You felt comfortable sleeping away from home (at a friend of family member's house or elsewhere)		option			4 = Very True<br>3 = Mostly True<br>2 = Somewhat True<br>1 = Not at all True
**14.2**	CFQR - Ages 12 and 13	23	cfqr_ages_12_13_q23	You felt left out		option			1 = Very True<br>2 = Mostly True<br>3 = Somewhat True<br>4 = Not at all True
**14.2**	CFQR - Ages 12 and 13	24	cfqr_ages_12_13_q24	You often invited friends to your house		option			4 = Very True<br>3 = Mostly True<br>2 = Somewhat True<br>1 = Not at all True
**14.2**	CFQR - Ages 12 and 13	25	cfqr_ages_12_13_q25	You were teased by other children		option			1 = Very True<br>2 = Mostly True<br>3 = Somewhat True<br>4 = Not at all True
**14.2**	CFQR - Ages 12 and 13	26	cfqr_ages_12_13_q26	You felt comfortable discussion your illness with others (friends, teachers)		option			4 = Very True<br>3 = Mostly True<br>2 = Somewhat True<br>1 = Not at all True
**14.2**	CFQR - Ages 12 and 13	27	cfqr_ages_12_13_q27	You thought you were too short		option			1 = Very True<br>2 = Mostly True<br>3 = Somewhat True<br>4 = Not at all True
**14.2**	CFQR - Ages 12 and 13	28	cfqr_ages_12_13_q28	You thought you were too thin		option			1 = Very True<br>2 = Mostly True<br>3 = Somewhat True<br>4 = Not at all True
**14.2**	CFQR - Ages 12 and 13	29	cfqr_ages_12_13_q29	You thought you were physically different from others your age		option			1 = Very True<br>2 = Mostly True<br>3 = Somewhat True<br>4 = Not at all True
**14.2**	CFQR - Ages 12 and 13	30	cfqr_ages_12_13_q30	Doing your treatments bothered you		option			1 = Very True<br>2 = Mostly True<br>3 = Somewhat True<br>4 = Not at all True
**14.2**	CFQR - Ages 12 and 13	31	cfqr_ages_12_13_q31	You coughed during the day		option			1 = Always<br>2 = Often<br>3 = Sometimes<br>4 = Never
**14.2**	CFQR - Ages 12 and 13	32	cfqr_ages_12_13_q32	You woke up during the night because you were coughing		option			1 = Always<br>2 = Often<br>3 = Sometimes<br>4 = Never
**14.2**	CFQR - Ages 12 and 13	33	cfqr_ages_12_13_q33	You had to cough up mucus		option			1 = Always<br>2 = Often<br>3 = Sometimes<br>4 = Never
**14.2**	CFQR - Ages 12 and 13	34	cfqr_ages_12_13_q34	You had trouble breathing		option			1 = Always<br>2 = Often<br>3 = Sometimes<br>4 = Never
**14.2**	CFQR - Ages 12 and 13	35	cfqr_ages_12_13_q35	Your stomach hurt		option			1 = Always<br>2 = Often<br>3 = Sometimes<br>4 = Never
**14.3**	CFQR - Adolescent/Adult		cfqr_adol_adult_datetime	CFQR completion date/time		datetime			
**14.3**	CFQR - Adolescent/Adult	1	cfqr_adol_adult_q1	Performing vigorous activities such as running or playing sports		option			1 = A lot of difficulty<br>2 = Some difficulty<br>3 = A little difficulty<br>4 = No difficulty
**14.3**	CFQR - Adolescent/Adult	2	cfqr_adol_adult_q2	Walking as fast as others		option			1 = A lot of difficulty<br>2 = Some difficulty<br>3 = A little difficulty<br>4 = No difficulty
**14.3**	CFQR - Adolescent/Adult	3	cfqr_adol_adult_q3	Carrying or lifting heavy things such as books, shopping, or school bags		option			1 = A lot of difficulty<br>2 = Some difficulty<br>3 = A little difficulty<br>4 = No difficulty
**14.3**	CFQR - Adolescent/Adult	4	cfqr_adol_adult_q4	Climbing one flight of stairs		option			1 = A lot of difficulty<br>2 = Some difficulty<br>3 = A little difficulty<br>4 = No difficulty
**14.3**	CFQR - Adolescent/Adult	5	cfqr_adol_adult_q5	Climbing stairs as fast as others		option			1 = A lot of difficulty<br>2 = Some difficulty<br>3 = A little difficulty<br>4 = No difficulty
**14.3**	CFQR - Adolescent/Adult	6	cfqr_adol_adult_q6	You felt well		option			4 = Always<br>3 = Often<br>2 = Sometimes<br>1 = Never
**14.3**	CFQR - Adolescent/Adult	7	cfqr_adol_adult_q7	You felt worried		option			1 = Always<br>2 = Often<br>3 = Sometimes<br>4 = Never
**14.3**	CFQR - Adolescent/Adult	8	cfqr_adol_adult_q8	You felt useless		option			1 = Always<br>2 = Often<br>3 = Sometimes<br>4 = Never
**14.3**	CFQR - Adolescent/Adult	9	cfqr_adol_adult_q9	You felt tired		option			1 = Always<br>2 = Often<br>3 = Sometimes<br>4 = Never
**14.3**	CFQR - Adolescent/Adult	10	cfqr_adol_adult_q10	You felt full of energy		option			4 = Always<br>3 = Often<br>2 = Sometimes<br>1 = Never
**14.3**	CFQR - Adolescent/Adult	11	cfqr_adol_adult_q11	You felt exhausted		option			1 = Always<br>2 = Often<br>3 = Sometimes<br>4 = Never
**14.3**	CFQR - Adolescent/Adult	12	cfqr_adol_adult_q12	You felt sad		option			1 = Always<br>2 = Often<br>3 = Sometimes<br>4 = Never
**14.3**	CFQR - Adolescent/Adult	13	cfqr_adol_adult_q13	To what extent do you have difficulty walking?		option			4 = You can walk a long time without getting tired<br>3 = You can walk a long time but you get tired<br>2 = You cannot walk a long time because you get tire<br>1 = You avoid walking whenever possible because it'
**14.3**	CFQR - Adolescent/Adult	14	cfqr_adol_adult_q14	How do you feel about eating?		option			1 = Just thinking about food makes you feel sick<br>2 = You never enjoy eating<br>3 = You are sometimes able to enjoy eating<br>4 = You are always able to enjoy eating
**14.3**	CFQR - Adolescent/Adult	15	cfqr_adol_adult_q15	To what extent do your treatments make your daily life more difficult?		option			4 = Not at all<br>3 = A little<br>2 = Moderately<br>1 = A lot
**14.3**	CFQR - Adolescent/Adult	16	cfqr_adol_adult_q16	How much time do you currently spend each day on your treatments?		option			1 = A lot<br>2 = Some<br>3 = A little<br>4 = Not very much

**14.3**	CFQR - Adolescent/Adult	17	cfqr_adol_adult_q17	How difficult is it for you to do your treatments (including medications) each day?		option			4 = Not at all<br>3 = A little<br>2 = Moderately<br>1 = Very
**14.3**	CFQR - Adolescent/Adult	18	cfqr_adol_adult_q18	How do you think your health is now?		option			4 = Excellent<br>3 = Good<br>2 = Fair<br>1 = Poor
**14.3**	CFQR - Adolescent/Adult	19	cfqr_adol_adult_q19	I have trouble recovering after physical effort		option			1 = Very true<br>2 = Somewhat true<br>3 = Somewhat false<br>4 = Very false
**14.3**	CFQR - Adolescent/Adult	20	cfqr_adol_adult_q20	I have to limit vigorous activities such as running or playing sports		option			1 = Very true<br>2 = Somewhat true<br>3 = Somewhat false<br>4 = Very false
**14.3**	CFQR - Adolescent/Adult	21	cfqr_adol_adult_q21	I have to force myself to eat		option			1 = Very true<br>2 = Somewhat true<br>3 = Somewhat false<br>4 = Very false
**14.3**	CFQR - Adolescent/Adult	22	cfqr_adol_adult_q22	I have to stay at home more than I want to		option			1 = Very true<br>2 = Somewhat true<br>3 = Somewhat false<br>4 = Very false
**14.3**	CFQR - Adolescent/Adult	23	cfqr_adol_adult_q23	I feel comfortable discussing my illness with others		option			4 = Very true<br>3 = Somewhat true<br>2 = Somewhat false<br>1 = Very false
**14.3**	CFQR - Adolescent/Adult	24	cfqr_adol_adult_q24	I think I am too thin		option			1 = Very true<br>2 = Somewhat true<br>3 = Somewhat false<br>4 = Very false
**14.3**	CFQR - Adolescent/Adult	25	cfqr_adol_adult_q25	I think I look different from others my age		option			1 = Very true<br>2 = Somewhat true<br>3 = Somewhat false<br>4 = Very false
**14.3**	CFQR - Adolescent/Adult	26	cfqr_adol_adult_q26	I feel bad about my physical appearance		option			1 = Very true<br>2 = Somewhat true<br>3 = Somewhat false<br>4 = Very false
**14.3**	CFQR - Adolescent/Adult	27	cfqr_adol_adult_q27	People are afraid that I may be contagious		option			1 = Very true<br>2 = Somewhat true<br>3 = Somewhat false<br>4 = Very false
**14.3**	CFQR - Adolescent/Adult	28	cfqr_adol_adult_q28	I get together with my friends a lot		option			4 = Very true<br>3 = Somewhat true<br>2 = Somewhat false<br>1 = Very false
**14.3**	CFQR - Adolescent/Adult	29	cfqr_adol_adult_q29	I think my coughing bothers others		option			1 = Very true<br>2 = Somewhat true<br>3 = Somewhat false<br>4 = Very false
**14.3**	CFQR - Adolescent/Adult	30	cfqr_adol_adult_q30	I feel comfortable going out at night		option			4 = Very true<br>3 = Somewhat true<br>2 = Somewhat false<br>1 = Very false
**14.3**	CFQR - Adolescent/Adult	31	cfqr_adol_adult_q31	I often feel lonely		option			1 = Very true<br>2 = Somewhat true<br>3 = Somewhat false<br>4 = Very false
**14.3**	CFQR - Adolescent/Adult	32	cfqr_adol_adult_q32	I feel healthy		option			4 = Very true<br>3 = Somewhat true<br>2 = Somewhat false<br>1 = Very false
**14.3**	CFQR - Adolescent/Adult	33	cfqr_adol_adult_q33	It is difficult to make plans for the future (for example, going to college, getting married, getting promoted at work, etc.)		option			1 = Very true<br>2 = Somewhat true<br>3 = Somewhat false<br>4 = Very false
**14.3**	CFQR - Adolescent/Adult	34	cfqr_adol_adult_q34	I lead a normal life		option			4 = Very true<br>3 = Somewhat true<br>2 = Somewhat false<br>1 = Very false
**14.3**	CFQR - Adolescent/Adult	35	cfqr_adol_adult_q35	To what extent did you have trouble keeping up with your schoolwork, professional work, or other daily activities during the past two weeks?		option			4 = You have had no trouble keeping up<br>3 = You have managed to keep up but it's been diffic<br>2 = You have been behind<br>1 = You have not been able to do these activities at a
**14.3**	CFQR - Adolescent/Adult	36	cfqr_adol_adult_q36	How often were you absent from school, work, or unable to complete daily activities during the last two weeks because of your illness or treatments?		option			1 = Always<br>2 = Often<br>3 = Sometimes<br>4 = Never
**14.3**	CFQR - Adolescent/Adult	37	cfqr_adol_adult_q37	How often does CF get in the way of meeting your school, work, or personal goals?		option			1 = Always<br>2 = Often<br>3 = Sometimes<br>4 = Never
**14.3**	CFQR - Adolescent/Adult	38	cfqr_adol_adult_q38	How often does CF interfere with getting out of the house to run errands such as shopping or going to the bank?		option			1 = Always<br>2 = Often<br>3 = Sometimes<br>4 = Never
**14.3**	CFQR - Adolescent/Adult	39	cfqr_adol_adult_q39	Have you had trouble gaining weight?		option			1 = A great deal<br>2 = Somewhat<br>3 = A little<br>4 = Not at all
**14.3**	CFQR - Adolescent/Adult	40	cfqr_adol_adult_q40	Have you been congested?		option			1 = A great deal<br>2 = Somewhat<br>3 = A little<br>4 = Not at all
**14.3**	CFQR - Adolescent/Adult	41	cfqr_adol_adult_q41	Have you been coughing during the day?		option			1 = A great deal<br>2 = Somewhat<br>3 = A little<br>4 = Not at all
**14.3**	CFQR - Adolescent/Adult	42	cfqr_adol_adult_q42	Have you had to cough up mucus?		option			1 = A great deal<br>2 = Somewhat<br>3 = A little<br>4 = Not at all
**14.3**	CFQR - Adolescent/Adult	43	cfqr_adol_adult_q43	Has your mucus been mostly:		option			1 = Clear<br>2 = Clear to yellow<br>3 = Yellowish-green<br>4 = Green with traces of blood<br>5 = Don't know
**14.3**	CFQR - Adolescent/Adult	44	cfqr_adol_adult_q44	Have you been wheezing?		option			1 = Always<br>2 = Often<br>3 = Sometimes<br>4 = Never
**14.3**	CFQR - Adolescent/Adult	45	cfqr_adol_adult_q45	Have you had trouble breathing?		option			1 = Always<br>2 = Often<br>3 = Sometimes<br>4 = Never
**14.3**	CFQR - Adolescent/Adult	46	cfqr_adol_adult_q46	Have you woken up during the night because you were coughing?		option			1 = Always<br>2 = Often<br>3 = Sometimes<br>4 = Never
**14.3**	CFQR - Adolescent/Adult	47	cfqr_adol_adult_q47	Have you had problems with wind?		option			1 = Always<br>2 = Often<br>3 = Sometimes<br>4 = Never
**14.3**	CFQR - Adolescent/Adult	48	cfqr_adol_adult_q48	Have you had diarrhea?		option			1 = Always<br>2 = Often<br>3 = Sometimes<br>4 = Never
**14.3**	CFQR - Adolescent/Adult	49	cfqr_adol_adult_q49	Have you had abdominal pain?		option			1 = Always<br>2 = Often<br>3 = Sometimes<br>4 = Never
**14.3**	CFQR - Adolescent/Adult	50	cfqr_adol_adult_q50	Have you had eating problems?		option			1 = Always<br>2 = Often<br>3 = Sometimes<br>4 = Never
**14.4**	CRISS		criss_datetime	Completion date/time		datetime			
**14.4**	CRISS	1	criss_q1	How difficult was it to breathe?		option			0 = Not difficult<br>1 = A little difficult<br>2 = Somewhat difficult<br>3 = A good deal difficult<br>4 = A great deal difficult
**14.4**	CRISS	2	criss_q2	How feverish did you feel (have a temperature)?		option			0 = Not feverish<br>1 = A little feverish<br>2 = Somewhat feverish<br>3 = A good deal feverish<br>4 = A great deal feverish
**14.4**	CRISS	3	criss_q3	How tired did you feel?		option			0 = Not tired<br>1 = A little tired<br>2 = Somewhat tired<br>3 = A good deal tired<br>4 = A great deal tired
**14.4**	CRISS	4	criss_q4	How bad were your chills or sweats		option			0 = No chills or sweat<br>1 = Slightly Bad<br>2 = Moderately Bad<br>3 = Very Bad<br>4 = Extremely Bad
**14.4**	CRISS	5	criss_q5	How bad was your cough?		option			0 = No cough<br>1 = Slightly Bad<br>2 = Moderately Bad<br>3 = Very Bad<br>4 = Extremely Bad
**14.4**	CRISS	6	criss_q6	How much mucus did you cough up?		option			0 = No mucus<br>1 = A little mucus<br>2 = Some mucus<br>3 = A good deal of mucus<br>4 = A great deal of mucus
**14.4**	CRISS	7	criss_q7	How much tightness in the chest did you have?		option			0 = No tightness<br>1 = A little tightness<br>2 = Some tightness<br>3 = A good deal of tightness<br>4 = A great deal of tightness
**14.4**	CRISS	8	criss_q8	How bad was your wheezing?		option			0 = No wheezing<br>1 = Slightly Bad<br>2 = Moderately Bad<br>3 = Very Bad<br>4 = Extremely Bad
**15**	Total IgE	1.1	total_ige_date	Date of sample		date			
**15**	Total IgE	1.2	total_ige_result	Result		decimal	IU/ml		
**16**	Aspergillus Specific RAST	1.1	rast_date	Date of sample		date			
**16**	Aspergillus Specific RAST	1.2	rast_result	Result		decimal	IU/ml		
**17**	Adverse Events		intensive_therapy_id	Intensive therapy ID		linked_form			
**17**	Adverse Events	1.1	aedesc	Event description (please give diagnosis if possible)		string			
**17**	Adverse Events	1.2	aeserious	Serious		boolean			1 = Yes<br>0 = No
**17**	Adverse Events	1.2.1	aeseriousrsn	What is the reason this event is considered serious?		option			1 = Occurred during the period of Intensive Therapy a<br>2 = Occurred during the period of Intensive Therapy a<br>3 = Occurred during the period of Intensive Therapy a<br>4 = Related to Assigned Interventions (at any time)
**17**	Adverse Events	1.3	aeonsetdt	Onset date		date			
**17**	Adverse Events	1.4	aetrt	Any treatments given for this event?		boolean			1 = Yes<br>0 = No

**17**	Adverse Events	1.5	aesev	Severity		option			1 = Mild<br>2 = Moderate<br>3 = Severe
**17**	Adverse Events	1.6.1.1	aebkabxrel	Relationship to assigned intervention in Backbone Antibiotic		option			1 = Related<br>2 = Probably related<br>3 = Possibly related<br>4 = Not related
**17**	Adverse Events	1.6.1.2	aebkabxadj	Were there any adjustments to the assigned intervention in Backbone Antibiotic attributed to this event?		option			0 = No<br>1 = Dose change<br>2 = Temporarily stopped<br>3 = Discontinued completely
**17**	Adverse Events	1.6.2.1	aeadabxrel	Relationship to assigned intervention in Adjunct Antibiotic		option			1 = Related<br>2 = Probably related<br>3 = Possibly related<br>4 = Not related
**17**	Adverse Events	1.6.2.2	aeadabxadj	Were there any adjustments to the assigned intervention in Adjunct Antibiotic attributed to this event?		option			0 = No<br>1 = Dose change<br>2 = Temporarily stopped<br>3 = Discontinued completely
**17**	Adverse Events	1.7	aeoutcome	Outcome		option			1 = Resolved<br>2 = Resolved with sequelea<br>3 = Not resolved
**17**	Adverse Events	1.8	aeresdt	Resolution date		date			
**18**	End Of Study	1.1	eosdat	Date participation in study completed		date			
**18**	End Of Study	1.2	eosreas	Reason for end of study		option			1 = Withdrawal<br>2 = Lung Transplant<br>3 = Participant deceased<br>4 = Other
**18**	End Of Study	1.2.1	eoswithreas	Reason for withdrawal		option			1 = Participant decision<br>2 = Investigator decision<br>3 = Sponsor decision<br>4 = Other
**18**	End Of Study	1.2.2	eostransdat	Date of Transplant		date			
**18**	End Of Study	1.2.3.1	eosdthdat	Date of Death		date			
**18**	End Of Study	1.2.3.2	eosdthcaus	Primary cause of Death		string			
**18**	End Of Study	1.2.3.3	eosdthawdat	Date of study staff awareness of death		date			
**18**	End Of Study	1.2.4	eosspec	Specify other reason		string			

required	rules
Yes	

If [genotype1] = 995	

If [genotype1] is entered, Unless [genotype2] = nil	

If [genotype2] = 995	

If [genotype2] is entered, Unless [genotype3] = nil	

If [genotype3] = 999	
Unless [sweat_chloride_level_na] is entered	between 0 and 500
No	
Yes	
Yes	
Yes	<embedded code>
Yes	<embedded code>
Yes	
If [abxreact] is Yes	
Yes	after date of 1st consent<br>on or before today's date<br>already exists for this participant
Yes	
If [cvpladmyn] is Yes	on or after clinic visit date<br>no more than 1 month in the future
Yes	
If [cvhgtupdyn] is Yes	<embedded code>
Yes	
If [cvwgtupdyn] is Yes	<embedded code>
Yes	
Yes	
Yes	
Yes	
Yes	
Yes	
Yes	no more than 2 years prior to BEAT CF date of 1st consent<br>on or before today's date
Yes	
If [sample_source] is entered	
Yes	
If [growth_detected] is Yes	
Yes	
Yes	already exists for this microbiology sample
If <embedded code>	
No	
If <embedded code>	
If [organism_type] = 1	
If [organism_type] = 2	
If <embedded code>	
If [antibiotic_susceptibility_tested] is Yes	
If [antibiotic_type_1] = 999	
If [antibiotic_type_1] is entered	
If [antibiotic_susceptibility_tested] = Yes, Unless [antibiotic_type_2] = nil Or [antibiotic_type_1] = nil	
If [antibiotic_type_2] = 999	
If [antibiotic_type_2] is entered	

If [antibiotic_susceptibility_tested] = Yes, Unless [antibiotic_type_3] = nil Or [antibiotic_type_2] = nil	
If [antibiotic_type_3] = 999	
If [antibiotic_type_3] is entered	
If [antibiotic_susceptibility_tested] = Yes, Unless [antibiotic_type_4] = nil Or [antibiotic_type_3] = nil	
If [antibiotic_type_4] = 999	
If [antibiotic_type_4] is entered	
If [antibiotic_susceptibility_tested] = Yes, Unless [antibiotic_type_5] = nil Or [antibiotic_type_4] = nil	
If [antibiotic_type_5] = 999	
If [antibiotic_type_5] is entered	
If [antibiotic_susceptibility_tested] = Yes, Unless [antibiotic_type_6] = nil Or [antibiotic_type_5] = nil	
If [antibiotic_type_6] = 999	
If [antibiotic_type_6] is entered	
If [antibiotic_susceptibility_tested] = Yes, Unless [antibiotic_type_7] = nil Or [antibiotic_type_6] = nil	
If [antibiotic_type_7] = 999	
If [antibiotic_type_7] is entered	

If [antibiotic_susceptibility_tested] = Yes, Unless [antibiotic_type_8] = nil Or [antibiotic_type_7] = nil	
If [antibiotic_type_8] = 999	
If [antibiotic_type_8] is entered	
If [antibiotic_susceptibility_tested] = Yes, Unless [antibiotic_type_9] = nil Or [antibiotic_type_8] = nil	
If [antibiotic_type_9] = 999	
If [antibiotic_type_9] is entered	
If [antibiotic_susceptibility_tested] = Yes, Unless [antibiotic_type_10] = nil Or [antibiotic_type_9] = nil	
If [antibiotic_type_10] = 999	
If [antibiotic_type_10] is entered	
If [antibiotic_susceptibility_tested] = Yes, Unless [antibiotic_type_11] = nil Or [antibiotic_type_10] = nil	
If [antibiotic_type_11] = 999	
If [antibiotic_type_11] is entered	
If [antibiotic_susceptibility_tested] = Yes, Unless [antibiotic_type_12] = nil Or [antibiotic_type_11] = nil	
If [antibiotic_type_12] = 999	
If [antibiotic_type_12] is entered	

If [antibiotic_susceptibility_tested] = Yes, Unless [antibiotic_type_13] = nil Or [antibiotic_type_12] = nil	
If [antibiotic_type_13] = 999	
If [antibiotic_type_13] is entered	
If [antibiotic_susceptibility_tested] = Yes, Unless [antibiotic_type_14] = nil Or [antibiotic_type_13] = nil	
If [antibiotic_type_14] = 999	
If [antibiotic_type_14] is entered	
If [antibiotic_susceptibility_tested] = Yes, Unless [antibiotic_type_15] = nil Or [antibiotic_type_14] = nil	
If [antibiotic_type_15] = 999	
If [antibiotic_type_15] is entered	
If [antibiotic_susceptibility_tested] = Yes, Unless [antibiotic_type_16] = nil Or [antibiotic_type_15] = nil	
If [antibiotic_type_16] = 999	
If [antibiotic_type_16] is entered	
If [antibiotic_susceptibility_tested] = Yes, Unless [antibiotic_type_17] = nil Or [antibiotic_type_16] = nil	
If [antibiotic_type_17] = 999	
If [antibiotic_type_17] is entered	

If [antibiotic_susceptibility_tested] = Yes, Unless [antibiotic_type_18] = nil Or [antibiotic_type_17] = nil	
If [antibiotic_type_18] = 999	
If [antibiotic_type_18] is entered	
If [antibiotic_susceptibility_tested] = Yes, Unless [antibiotic_type_19] = nil Or [antibiotic_type_18] = nil	
If [antibiotic_type_19] = 999	
If [antibiotic_type_19] is entered	
If [antibiotic_susceptibility_tested] = Yes, Unless [antibiotic_type_20] = nil Or [antibiotic_type_19] = nil	
If [antibiotic_type_20] = 999	
If [antibiotic_type_20] is entered	
Yes	already exists for this participant
No	
No	
Yes	
If [contrarsn] = 1	

If [contracause] = No	
If [contraparent] = 9	
No	
No	
No	
No	
No	
No	
If [contrasrcoth] is entered	
If [contrarsn] = 1	
If [reactknown] is Yes	
If [reacttype] is one of 9, 10	
If [reacttype] = 10	
If [reacttype] = 10	
If [reactknown] is Yes	after date of birth<br>confirmed with data query if more than 10 years before BEA<br>on or before today's date
If [reactknown] is Yes	
If [reactassess] is Yes	after date reaction first recorded<br>on or before today's date
If [reactassess] is Yes	
Yes	
Yes	
If <embedded code>	
No	
No	on or before today's date
Yes	
Unless [cmongo] is Yes	on or after the start date<br>no more than 7 days in the future
Yes	
Yes	
Yes	no more than 14 days prior to date of 1st consent<br>no more than 5 days in the future
No	
No	
If [itherloc] = 1, Unless [itherhosp] = nil	
If [itherloc] = 1, Unless [admitweight] = nil	<embedded code>
If <embedded code>	
If [itrand] = No	
If [itrand] = No	
If [admitcoughtp] is entered, Unless [admitcoughtpna] is entered	
No	
If [admitcrepit] is Yes, Unless [admitcrepitna] is entered	
No	
No	
No	
No	
No	
Yes	

Yes	
Yes	already entered the same active antibiotic during this intens
Yes	on or after the start date of the intensive therapy admission<br>on or before the end date of the intensive therapy admissior<br>must not be in the future
Yes	
Yes	
Yes	
Yes	
If [abxfreq] = 8	
No	on or after the start date/time<br>on or before the end date of the intensive therapy admissior<br>must not be in the future
If [abxstopdatm] is entered	
If [abxstoprsn] = 6	
If [abxstopdatm] is entered	between 0 and 6
If <embedded code>	on or after the start date of the intensive therapy admission<br>on or before the end date of the intensive therapy admissior
If <embedded code>	on or after the start date of the intensive therapy admission<br>on or before the end date of the intensive therapy admissior
If <embedded code>	on or after the start date of the intensive therapy admission<br>on or before the end date of the intensive therapy admissior
If <embedded code>	on or after the start date of the intensive therapy admission<br>on or before the end date of the intensive therapy admissior
If <embedded code>	on or after the start date of the intensive therapy admission<br>on or before the end date of the intensive therapy admissior
If <embedded code>	on or after the start date of the intensive therapy admission<br>on or before the end date of the intensive therapy admissior
Yes	
Yes	
If [ittrt] = 99	
Yes	on or after the start date of the intensive therapy admission<br>on or before the end date of the intensive therapy admissior<br>on or before today's date
Yes	between 1 and 1000
Yes	
Yes	
No	on or after the prescription start date<br>on or before the end date of the intensive therapy admissior<br>on or before today's date
Yes	
Yes	
If [aitrt] = 3	
Yes	on or after the start date of the intensive therapy admission<br>on or before the end date of the intensive therapy admissior<br>on or before today's date
Yes	between 1 and 1000
Yes	
Yes	
Unless [aistopdat] = nil	on or after the prescription start date<br>on or before the end date of the intensive therapy admissior<br>on or before today's date
Yes	
Yes	on or after the start date of the intensive therapy admission<br>on or before the end date of the intensive therapy admissior<br>on or before today's date<br>already exists for this date
Yes	between 1 and 4
Yes	
If [phactacbt] is Yes	between 1 and 21
If [phactacbt] is Yes	
If [phactacbt] is Yes	
If [phactacbt] is Yes	
Yes	
If [phactaad] is Yes	between 1 and 21
If [phactaad] is Yes	

If [phactaad] is Yes	
If [phactaad] is Yes	
Yes	
If [phactpep] is Yes	between 1 and 21
If [phactpep] is Yes	
If [phactpep] is Yes	
If [phactpep] is Yes	
Yes	
If [phactopep] is Yes	between 1 and 21
If [phactopep] is Yes	
If [phactopep] is Yes	
If [phactopep] is Yes	
Yes	
If [phactbpep] is Yes	between 1 and 21
If [phactbpep] is Yes	
If [phactbpep] is Yes	
If [phactbpep] is Yes	
Yes	
If [phactniv] is Yes	between 1 and 21
If [phactniv] is Yes	
If [phactniv] is Yes	
If [phactniv] is Yes	
Yes	
If [phactman] is Yes	between 1 and 21
If [phactman] is Yes	
If [phactman] is Yes	
If [phactman] is Yes	
Yes	
If [phactvest] is Yes	between 1 and 21
If [phactvest] is Yes	
If [phactvest] is Yes	
If [phactvest] is Yes	
Yes	
If [phactexec] is Yes	between 1 and 21
If [phactexec] is Yes	
If [phactexec] is Yes	
If [phactexec] is Yes	
Yes	
If [phactmetaneb] is Yes	between 1 and 21
If [phactmetaneb] is Yes	
If [phactmetaneb] is Yes	
If [phactmetaneb] is Yes	
Yes	
If [phactoth] is Yes	
If [phactoth] is Yes	
If [phactoth] is Yes	
If [phactoth] is Yes	
If [phactoth] is Yes	
Yes	
If [phexeaero] is Yes	between 1 and 21
If [phexeaero] is Yes	
Yes	
If [phexeresist] is Yes	between 1 and 21
If [phexeresist] is Yes	
Yes	
If [phexehiit] is Yes	between 1 and 21
If [phexehiit] is Yes	
Yes	
If [phexeplay] is Yes	between 1 and 21
If [phexeplay] is Yes	
Yes	
If [phexeoth] is Yes	
If [phexeoth] is Yes	between 1 and 21
If [phexeoth] is Yes	
Yes	
Yes	after the start date of the intensive therapy admission<br>on or before today's date<br>already exists for this date
Unless [treatment_stopped_previously?]	
If [naxintrtyn] = No	the day of or day before Daily date<br>on or before Daily date
Yes	
If [naxdischyn] = No	
If [naxloc] = 1	
If [naxdischyn] = Yes	the day of or day before Daily date<br>no more than 7 days after last dose<br>on or before Daily date
Unless [caxcoughtpna] is entered	
No	
If [caxcoughtp] = 1	
Unless [caxcrepitna] is entered	
No	
Yes	
Yes	
Yes	

If [naxabxaddchg] is Yes	
Yes	
Yes	
Yes	
Yes	
Yes	
Yes	on or after the start date of the intensive therapy admission<br>on or before the end date of the intensive therapy admissio<br>on or before today's date
Yes	
If [phout6min] is Yes	between 0 and 1000
If [phout6min] is Yes	on or after the start date of the intensive therapy admission<br>on or before assessment date
Yes	
If [phoutmodsh] is Yes	between 1 and 25
If [phoutmodsh] is Yes	on or after the start date of the intensive therapy admission<br>on or before assessment date
If [phoutmodsh] is Yes	
Yes	
If [phoutstep] is Yes	between 0 and 15
If [phoutstep] is Yes	on or after the start date of the intensive therapy admission<br>on or before assessment date
Yes	
If [phoutcpet] is Yes	between 0 and 15
If [phoutcpet] is Yes	on or after the start date of the intensive therapy admission<br>on or before assessment date
Yes	
If [phoutoth] is Yes	
If [phoutoth] is Yes	
If [phoutoth] is Yes	on or after the start date of the intensive therapy admission<br>on or before assessment date
Yes	no more than 12 months prior to BEAT CF date of 1st conse<br>on or before the current time
Yes	between 0.2 and 12
Yes	<embedded code>
Yes	
Yes	
No	
Yes	on or after date of 1st consent<br>on or before today's date
Yes	
If [cdres] = 1	
If [cdres] = 1, Unless [previous_positive?]	
If [cdprevpos] is Yes	no more than 2 years before BEAT CF date of 1st consent<br>must not be in the future
Yes	
If [abrabx] = 999	
Yes	
Unless [abrstdatunk] is entered	on or after date of 1st consent<br>cannot be in the future
No	
Yes	
No	
No	
No	
No	
No	
No	
No	
No	
No	
No	
No	
No	
No	
No	
No	
No	
No	
No	
No	
No	
No	
No	
No	

No	
No	
No	
No	
No	
No	
No	
No	
No	
No	
No	
No	
No	
No	
No	
No	
No	
No	
No	
No	
No	
No	
No	
No	
No	
No	
No	
No	
No	
No	
Yes	
Yes	
Unless [abrdoseunk] is entered	between 1 and 100
No	
Yes	
Yes	
If [abrprevclas] = 1	
Yes	
Yes	
If [abrsympers] = 1	between 0.1 and 23.9
If [abrsympers] = 2	between 1 and 30
Yes	
No	
No	
No	
No	
No	
No	
No	
No	
No	
If [abrtrtoth] is entered	
Yes	
No	
No	
No	
No	
No	
No	
If [abrmedoth] is entered	
Yes	
If [abrsymnodrug] = 1	
Yes	
Yes	
Unless [abrwccna] is entered	between 4.5 and 11.0
No	
Unless [abreosinophilsna] is entered	between 0.04 and 2
No	
Unless [abrneutrophilsna] is entered	between 1.4 and 12
No	
Unless [abrhgbna] is entered	between 9 and 18
No	
Unless [abrpltna] is entered	between 150 and 400
No	
Unless [abraltna] is entered	between 5 and 40
No	
Unless [abrastna] is entered	between 5 and 35
No	
Unless [abrtbrna] is entered	between 1 and 20
No	
Unless [abrureana] is entered	between 2.5 and 7.1
No	
Unless [abrcreatna] is entered	between 10 and 110
No	
Unless [abrcrpna] is entered	between 0 and 40
No	
Unless [abrtrypna] is entered	between 0.1 and 13
No	

Yes	
No	
Yes	
Yes	confirm more than 10 years prior to date of 1st consent in B<br>on or before today's date
No	enrolled in other study during enrolment in BEAT CF<br>on or after enrolment date<br>on or before today's date
No	
No	
No	
No	
No	
No	
No	
No	
No	
No	
No	
No	
No	
No	
No	
No	
No	
No	
No	
No	
No	
No	
No	
No	
No	
No	
No	
No	
No	
No	
No	
No	
No	
No	
No	
No	
No	
No	
No	
No	
No	
No	
No	
No	
No	
No	
No	
No	

No	
No	
No	
No	
No	
No	
No	
No	
No	
No	
No	
No	
No	
No	
No	
No	
No	
No	
No	
No	
No	
No	
No	
No	
No	
No	
No	
No	
No	
No	
No	
No	
No	
No	
No	
No	
No	
No	
No	
No	
No	
No	
No	
No	
No	
No	
No	
No	
No	
No	

No	
No	
No	
No	
No	
No	
No	
No	
No	
No	
No	
No	
No	
No	
No	
No	
No	
No	
No	
No	
No	
No	
No	
No	
No	
No	
No	
No	
No	
No	
No	
No	
No	
No	
No	
No	
No	
No	
No	
No	
No	
No	
No	
Yes	no more than 2 years prior to BEAT CF date of 1st consent<br>on or before today's date
Yes	between 0 and 5000
Yes	no more than 2 years prior to BEAT CF date of 1st consent<br>on or before today's date
Yes	between 0 and 1000
Yes	
Yes	
Yes	
If [aeserious] is Yes	
Yes	on or after intensive therapy admission date<br>on or before intensive therapy end date<br>cannot be in the future
Yes	

Yes	
Yes	
Yes	
Yes	
Yes	
Yes	
If [resolved?]	on or after onset date<br>cannot be in the future
Yes	on or after date of 1st consent<br>on or before today's date
Yes	
If [eosreas] = 1	
If [eosreas] = 2	on or after date of 1st consent<br>on or before study completion date
If [eosreas] = 3	on or after date of 1st consent<br>on or before study completion date
If [eosreas] = 3	
If [eosreas] = 3	on or after death date<br>on or before the study completion date
If [eosreas] = 4	
